# Alternative splicing patterns reveal prognostic indicator in muscle-invasive bladder cancer

**DOI:** 10.1186/s12957-022-02685-0

**Published:** 2022-07-12

**Authors:** BaiHeTiYa AZhaTi, Gaoliang Wu, Hailun Zhan, Wei Liang, Zhijian Song, Leilei Lu, Qichao Xie

**Affiliations:** 1 Department of Urology, The First Affiliated Hospital of Xinjiang Medical University, No.137 South Carp Hill Road, Xinjiang Uygur Autonomous Region, Urumqi, Xinjiang Province People’s Republic of China; 2 Department of Urology at Cancer Hospital of Jiangxi Province, No.519 East Beijing Road, Qingshan Lake District, Nanchang, Jiangxi Province People’s Republic of China; 3 Department of Urology Third Affiliated Hospital, Sun Yat-Sen University, No.600 Tianhe Road, Guangzhou, Guangzhou Province People’s Republic of China; 4 Department of Oncology, The Third Affiliated Hospital of Chongqing Medical University, Yubei District, No. 1 Shuanghu Branch Road, Chongqing, 401120 People’s Republic of China; 5 OrigiMed, 5th Floor, Building 3, No.115 Xin Jun Huan Road, Minhang District, Shanghai, People’s Republic of China

**Keywords:** Alternative splicing, Muscle-invasive bladder cancer, Splicing factor, Percent spliced in, TCGA, LASSO Cox regression

## Abstract

**Background:**

Bladder cancer is one of the most lethal malignancy in urological system, and 20–25% of bladder cancer patients are muscle invasive with unfavorable prognosis. However, the role of alternative splicing (AS) in muscle-invasive bladder cancer (MIBC) remains to be elucidated.

**Methods:**

Percent spliced in (PSI) data obtained from the Cancer Genome Atlas (TCGA) SpliceSeq database (*n* = 394) were utilized to evaluate the AS events in MIBC. Prognosis-associated AS events were screened out by univariate Cox regression. LASSO Cox regression was used to identify reliable prognostic patterns in a training set and further validated in a test set. Splicing regulatory networks were constructed by correlations between PSI of AS events and RNA expression of splicing factors.

**Results:**

As a result, a total of 2589 prognosis-related AS events in MIBC were identified. Pathways of spliceosomal complex (*FDR* = 0.017), DNA-directed RNA polymerase II, core complex (*FDR* = 0.032), and base excision repair (*FDR* = 0.038) were observed to be significantly enriched. Additionally, we noticed that most of the prognosis-related AS events were favorable factors. According to the LASSO and multivariate Cox regression analyses, 15-AS-based signature was established with the area under curve (AUC) of 0.709, 0.823, and 0.857 at 1-, 3-, and 5- years, respectively. The MIBC patients were further divided into high- and low-risk groups based on median risk sores. Interestingly, we observed that the prevalence of FGFR3 with mutations and focal amplification was significantly higher in low-risk group. Functional and immune infiltration analysis suggested potential signaling pathways and distinct immune states between these two groups. Moreover, splicing correlation network displayed a regulatory mode of prognostic splicing factors (SF) in MIBC patients.

**Conclusions:**

This study not only provided novel insights into deciphering the possible mechanism of tumorgenesis and pathogenesis but also help refine risk stratification systems and potential treatment of decision-making for MIBC.

**Supplementary Information:**

The online version contains supplementary material available at 10.1186/s12957-022-02685-0.

## Introduction

Bladder cancer is one of the most lethal malignancy in urological system which leads to an estimate of 80,500 new cases per year in China [1]. As was reported, approximately 20–25% of patients with bladder cancer were muscle-invasive bladder cancer (MIBC) [2], and most of them progress, invade, and metastasize rapidly, resulting in a poor prognosis. Currently, radical cystectomy (RC) is considered as the golden standard treatments for MIBC. Although chemoradiotherapy is another suitable alternative, almost 50% of MIBC patients develop recurrence and metastasis during the 2 years of initial diagnosis and lastly succumb to relapses. Hence, it is especially important to clarify the mechanism involved in tumor progression and develop novel prognostic biomarkers for MIBC.

Recently, considerable efforts have been made to cover the underlying pathogenesis mechanisms and the discovery of molecular biomarkers and therapeutic targets in MIBC. For example, it was widely accepted that dysregulations of DNA genomic variants, copy number alternation, gene expressions, and DNA methylation were involved in the progression and metastasis in MIBC [3, 4]. However, although these studies have achieved progresses, most of them relied on mutational and gene expression levels while largely ignoring other contributors such as alternative splicing.

Alternative splicing (AS) is a posttranscriptional regulatory mechanism that generates transcriptomic and proteomic complexity by producing a diversity of alternative transcripts and encoding multiple protein isoforms. Notably, almost 95% of multi-exon genes suffer alternative splicing process in human beings [5–7]. Seven types of AS events are categorized as follows: exon skip (ES), alternate acceptor site (AA), alternate donor site (AD), alternate promoter (AP), alternate terminator (AT), retained intron (RI), and mutually exclusive exons (ME). Currently, with the development of next-generation sequencing technology, sophisticated computational tools have been developed for identifying AS events more precisely by application of short-read RNA-seq data. More importantly, several new trends of association between AS events and carcinogenesis hallmarks have been gradually emerging [8–11]. Despite the crucial role of AS in carcinogenesis, there is little known about the clinical associations and underlying regulatory mechanism of AS in muscle-invasive bladder cancer. Hence, identification of specific AS events for MIBC as prognostic or predictive biomarkers is promising and urgently needed.

In our study, systematic analyses of genome-wide alternative splicing events were performed in the MIBC cohort from The Cancer Genome Atlas (TCGA) project. Next, AS events associated with overall survival (OS) were identified, and reliable prognostic signatures based on a LASSO Cox regression model were constructed. Subsequently, gene set enrichment analysis (GSEA) and immune infiltration analysis were conducted to compare the perturbed functional pathways and immune cell infiltrations between the high- and low-risk groups in MIBC. Finally, prognosis-associated splicing factors and alternative splicing events (SF-AS) network were constructed to reveal the potential mechanism for MIBC. Our findings not only aim to uncover tumor-associated AS events but also intent to provide us great insights into predicting and evaluating the clinical outcomes, along with the underlying regulatory mechanisms of bladder cancer.

## Materials and methods

### TCGA RNAseq and alternative splicing event data process

In this study, the RNA-seq data (level 3) and clinical data of primary MIBC and adjacent non-tumor tissues were obtained from the TCGA database (https://tcga-data.nci.nih.gov/tcga/). Only patients (*n* = 394) with complete clinicopathological information and at least 30 days of OS were included in this study. To generate the AS profiling of MIBC cohorts, TCGA SpliceSeq, a web-based resource which generated mRNA splicing patterns of 33 types cancers (http://bioinformatics.mdanderson.org/TCGASpliceSeq), were used [12]. An index of percent splice in (PSI) ranged from 0 to 1 was calculated for each splice event across samples, which measures the ratio of inclusive reads normalized by length over both inclusive and exclusive reads for that event. In order to generate a reliable set of AS events, the criteria with a PSI value of ≥ 0.75 and a standard deviation of > 0.05 for all samples were required. The intersections of protein-coding genes among seven types of AS events in MIBC were analyzed with UpSet R package in R.

### Functional and pathway enrichment and PPI networks analysis

The Kyoto Encyclopedia of Genes and Genomes (KEGG) [13] and gene ontology [14] analysis for biological process (BP), cellular component (CC), and molecular function (MF) were investigated with clusterProfiler package [15] in R software at the significant level (adjust *P* < 0.05). The parent genes of the prognostic AS events(*P* < 0.001) were selected and mapped to a STRING database (version 10) using a score > 0.4, and Cytoscape [16] (version 3.7.1) was applied to visualize the PPI network.

### Survival analysis and the establishment of prognostic model

To explore the association between AS events and the prognosis of MIBC patients, Univariate Cox proportional hazard regression analysis was performed to identify significant OS-associated AS events with *P* < 0.05. In this study, Wilcoxon rank test was employed to compare the PSI values between tumor and normal tissues to identify differentially alternative splicing events, with an absolute median *PSI* > 0.2 and *FDR* < 0.05. To make our prognostic model more robust, a total of 394 MIBC patients was randomly divided into a training set (*n* = 197) and a test set (*n* = 197) at 1:1 ratio. The training set was constructed for the AS prognostic risk signature, and the test was applied for validation. The least absolute shrinkage and selection operator (LASSO) Cox analysis was used to select critical AS events using R package “glmnet.” Only AS with nonzero coefficients in the LASSO cox model were put into the multivariate Cox regression [17] to calculate the risk score. We used AS PSI levels weighted by a corresponding regression coefficient (β) from the multivariate Cox regression analysis, and the risk score formula for each patient was as follows: risk score = β_1_ × AS_1_ + β_2_ × AS_2_ + ··· + β_n_ × AS_n_. In order to assess the accuracy of prognostic model, Kaplan–Meier analysis with log-rank test was used to compare the patients’ survival difference between high- and low-risk subgroups. The area under the curve (AUC) of receiver operating characteristic (ROC)[18] was calculated using the survival ROC package for each splicing events type based on the prognostic model. All analyses were carried out using R software (version 3.5.3).

### Gene set enrichment analysis (GSEA)

To identify 15-AS-related pathways and biological processes, GSEA was conducted between the high- and low-risk groups, by using GSEA (version 3.0, http://www.broadinstitute.org/gsea/index.jsp) software. Canonical pathways (c2.all.v7.0.symbols.gmt) obtained from the Molecular Signatures Database (http://software.broadinstitute.org/gsea/msigdb/index.jsp) were used. For each analysis, a permutation test of 1000 times was required. Enriched gene sets with a nominal (NOM) *P*-value < 0.05 and |NES|> 1.5 were considered as significantly different.

### Immune infiltration analysis

Based on normalized expression profiles, CIBERSORT [19] algorithm was employed to calculate the relative abundance of immune infiltrating cell composition in tumors with normalized expression profiles. The gene expression dataset of 394 MIBC was uploaded to CIBERSORT website portal (http://cibersort.stanford.edu/) using LM22 signature matrix (a “signature matrix” of 22 immune cell types including 547 genes) at 1000 permutations. For each sample, the percentage of 22 immune cells from CIBERSORT was defined as immune infiltration fraction. Furthermore, we conducted a Wilcoxon rank-sum test to compare immune infiltration fractions between low- and high-risk groups. Finally, we used the “ComplexHeatmap” package in R software to illustrate the differential immune cells in MIBC, where the colors from blue to red represent the infiltrating levels from low to high. “ESTIMATE” package [20] in R software was used to assess the estimate score, stromal score, and immune score in tumors.

### Establishment of the nomogram and validation

A clinical application model was constructed to predict the OS of patients with MIBC. Clinical information (e.g., American Joint Committee on Cancer (AJCC) stage, patient age) and patient risk scores were enrolled as covariates in the Cox univariate and multivariate regression analyses to evaluate independent risk predictors for MIBC. In addition, we constructed a nomogram to predict survival with 15-AS risk score and other clinicopathological factors by using “rms” R package (https://cran.r-project.org/web/packages/rms/). To evaluate the accuracy of the nomogram, calibration curves were used to compare the concordance between predicted survival and observed survival. Additionally, the performance of the nomogram was assessed by C-index.

### Construction of the splicing correlation network splicing factor networks

To explore the prognostic AS events regulated by splicing factors, splicing factors were extracted from the SpliceAid2 database [21] (www.introni.it/spliceaid.html). The mRNA expressions of splicing factors were downloaded from TCGA portal (https://tcga-data.nci.nih.gov/tcga/). To find potential regulatory network between the prognosis-associated SFs and AS events, the Spearman test was performed to analyze the correlation between them. Pairs of splicing factor (SF) genes associated with PSI values were selected as the candidates with the criteria of (*r* > 0.5, *P* < 0.01) in the splicing regulatory network. Cytoscape (version 3.7.1) was used to establish the interaction network between the significant SFs and AS events.

## Results

### Overview of AS events profiling in MIBC cohort

**Fig. 1:**
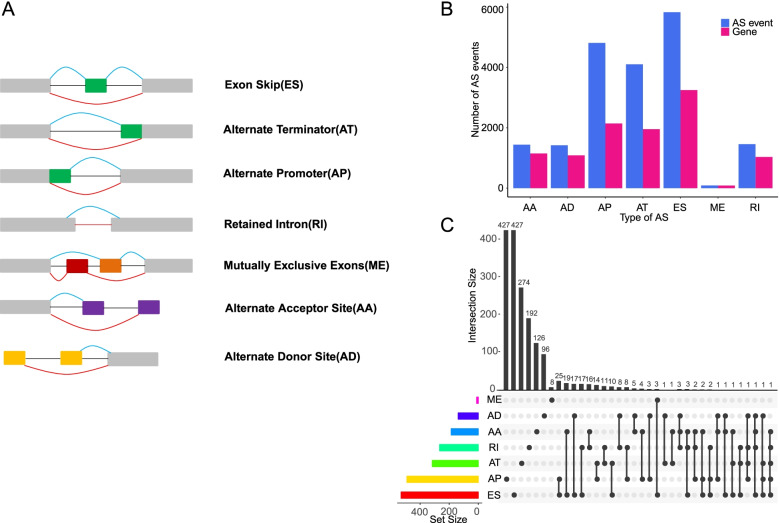
Overall characteristics of seven types of aberrant alternative splicing (AS) in MIBC. **A** Illustrations for 7 types of AS events. ES, exon skip; AT, alternate terminator; AP, alternate promoter; RI, retained intron; ME, mutually exclusive exons; AA, alternate acceptor site; AD, alternate donor site. **B** Number of AS events and involved parent genes from the 394 MIBC patients. **C** The UpSet intersection plot of AS events in MIBC

The comprehensive AS events profiling of 394 MIBC patients combined with clinical data was curated from TCGA SpliceSeq data and median follow-up OS among which was 18 months (range from 1 to 168 months). AS events of seven types were depicted in Fig. 1A. In total, 19,159 AS events from 6968 genes were detected, comprised of 5834 ESs in 3249 genes, 1459 RIs in 1032 genes, 4814 APs in 2144 genes, 4104 ATs in 1955 genes, 1420 ADs in 1085 genes, 1441 AAs in 1149 genes, and 87 MEs in 85 genes, respectively (Fig. 1B). These data revealed that one single gene harbored almost four AS events on average, which implied that the different arrangements and reconstructions of splicing types might play a vital role in the transcriptomic isoform diversity. Besides, ES events were the predominant frequent type, which account for nearly a third of all of the AS types, followed by AP and AT types. Moreover, we analyzed the variation of seven common splice events in different age groups and gender. For the age group, we divided the patients into two groups with the cutoff of 60 years old. Interestingly, we observed that alternative splicing events were significantly higher in age < 60 group than in age >  = 60 group at the AS patterns of AA, AD, AP, ES, and RI (Supplementary Fig. 1). While compared between gender groups, only AA pattern was found to be significantly higher in male than in female group (Supplementary Fig. 2).

### Survival-associated AS events in MIBC cohort

**Fig. 2:**
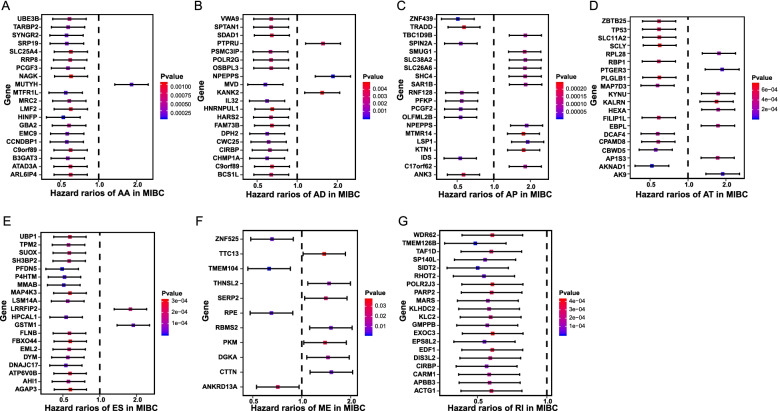
Forest plots for subgroup analyses of the top 20 survival-associated AS events in MIBC. **A**–**G** Forest plots of top 20 survival-associated AS in AA subgroup (**A**), AD subgroup (**B**), AP subgroup (**C**), AT subgroup (**D**), ES subgroup (**E**), ME subgroup (**F**), and RI subgroup (**G**) events. The circles represent hazard ratios, and horizontal bars represent 95% confidence intervals

To assess the AS events related to OS in MIBC cohort, Cox univariate analyses were conducted to identify the prognostic factors for each type of AS events. MIBC patients were divided into low- and high-PSI groups according to the median cutoff of PSI value. Consequently, a total of 2589 AS events were identified as the most significantly prognosis-associated AS events in MIBC (*P* < 0.05, Supplementary Table 1), which accounted for 13.5% of all AS events and 24.8% of their patent genes in MIBC. As illustrated by UpSet plot (Fig. 1C), we found that most prognosis-associated AS gene could possess at least two AS event types, and some of them even had five AS event types. For instance, AT and ES of TP53 were found to be significantly associated with OS. Notably, we observed that most of these significantly prognosis-associated AS events (1697 ASs in 2589 ASs) were favorable prognostic factors (*HR* < 1). Hazard ratios (HRs) with 95% confidence intervals (95% CI) of the top 20 most significant prognosis-associated AS events (if available) would be visualized in forest plots (Fig. 2). Obviously, most of the AS events in AA, AD, ES, and RI were favorable prognostic factors.

### Identification and functional enrichment analysis of prognosis-associated AS events

**Fig. 3:**
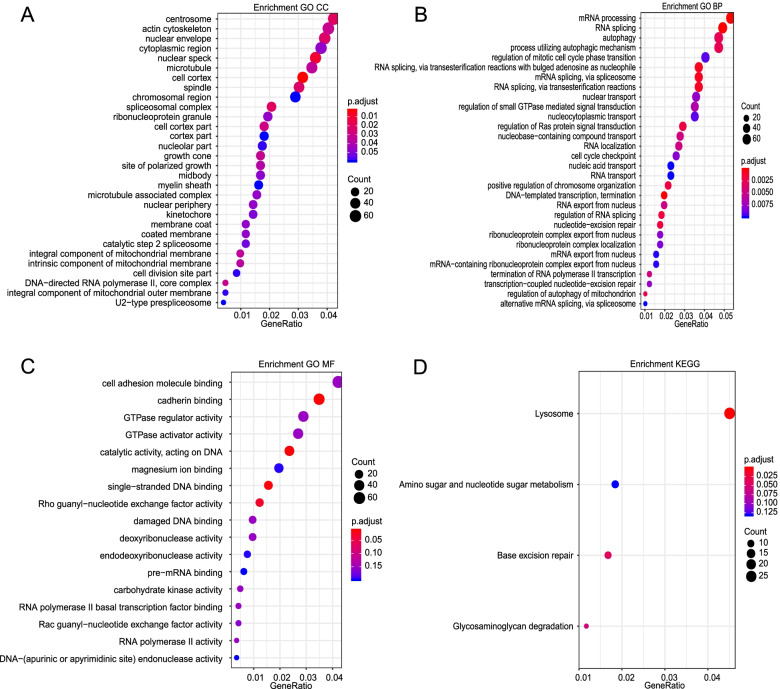
Functional KEGG and GO enrichment analyses for parent genes of survival-associated AS events. The vertical axis represents GO or KEGG pathway annotations. The horizontal axis represents the number of genes assigned to the corresponding annotation. **A** The top 30 significant terms of CC. **B** The top 30 significant terms of BP. **C** The top 30 significant terms of MF. **D** The top 4 significant terms of KEGG pathways. BP, biological process; CC, cellular component; MF, molecular function

**Fig. 4:**
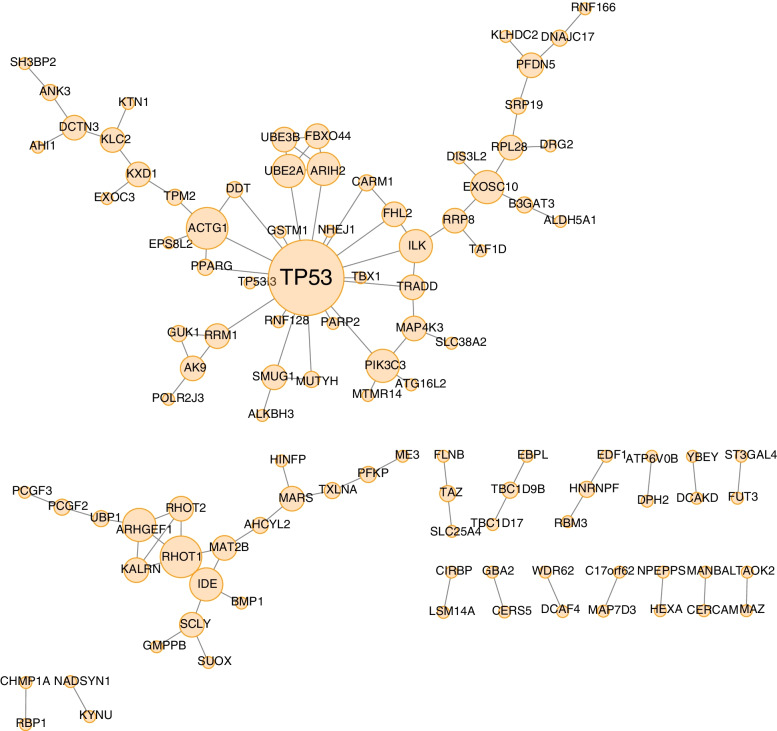
Protein–protein interaction network for survival associated AS events. In this plot, nodes stand for genes, and lines denote their interactions, and the size of the nodes represents degree values

In addition, to evaluate the potential impact of prognosis-associated AS events on their corresponding genes, GO and KEGG pathway enrichment analyses were conducted to their parent genes of prognosis-associated AS events in MIBC. A total of 23 significant terms in cellular component (CC) were enriched, including related pathways of spliceosomal complex (*FDR* = 0.017) and DNA-directed RNA polymerase II and core complex (*FDR* = 0.032) (CC, Fig. 3A). Besides, 80 pathways in biological process (BP, Fig. 3B) and 4 pathways in molecular function (MF, Fig. 3C) were also highlighted, indicating significant enrichment in terms of RNA splicing (*FDR* < 0.001), DNA-templated transcription, termination (*FDR* < 0.001), and mRNA processing (*FDR* < 0.001). Additionally, 3 specific KEGG pathways with FDR value < 0.05 were considered to be significantly enriched in MIBC, including lysosome (*FDR* = 0.001), base excision repair (*FDR* = 0.038), and glycosaminoglycan degradation (*FDR* = 0.054). Top significant enriched KEGG pathway terms were shown in Fig. 3D. And the detailed information of functional enrichment analysis of prognosis-associated AS events was listed in Supplementary Table 2. Taken together, these results strongly implied that the genes of prognosis-associated AS events played pivotal roles in cancer-related biological processes, which could help to uncover potential regulatory mechanisms of AS events toward protein function in MIBC.

### Gene network for survival-associated AS events

To explore the interactions among parent genes of prognosis-associated AS events, a gene network was constructed in MIBC. Parent genes of prognosis-associated AS events (*P* < 0.001) in MIBC were mapped to the string protein–protein interaction database with a score > 0.4. A PPI network visualized by Cytoscape illustrated the key genes (TP53, IDE, and PIK3C3, etc.) and their interactions in the survival-associated network (Fig. 4). According to the results, we observed that most of the parent genes in prognosis-associated AS events exhibited direct protein–protein interactions to each other, which implied that they were jointly involved in diverse biological functions in MIBC.

### Construction of a 15-AS-based signature in MIBC

**Fig. 5:**
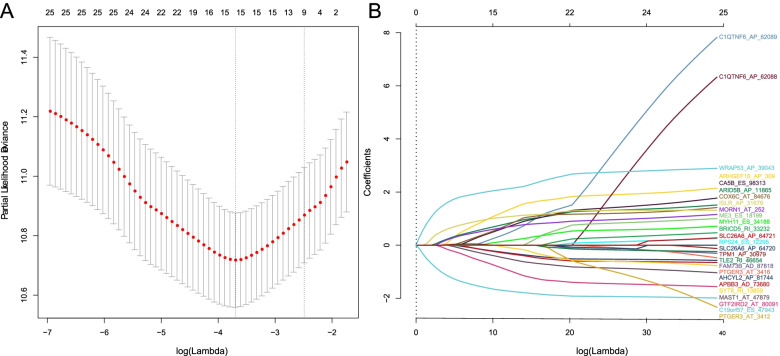
Identification of 15-AS signature related to overall survival (OS) by LASSO regression analysis. **A** Partial likelihood deviance with corresponding log(λ) at the minimal deviance. **B** LASSO coefficient for selecting 15 prognostic AS by tenfold cross-validation

To explore the prognostic values of AS patterns in MIBC, a total of 394 MIBC patients were randomly divided into a training set (*n* = 197) for construction and a test set (*n* = 197) for validation. Clinical characteristics of 394 MIBC patients were shown in Table 1. Differential alternative splicing (DAS) analysis between tumors and normal tissues identified that 1079 AS events were differentially expressed, interestingly, of which 175 DAS events were associated with OS. Next, to reduce the bias caused by a given type of AS event, we conducted LASSO Cox regression (Fig. 5) that considered all types of AS events in the training set and identified a survival-associated AS signature of 15 AS events. Finally, the AS risk score was generated by PSI values and coefficients from multivariate Cox regression. The formula was obtained as follows:

**Table 1:** Summary of TCGA MIBC patient demographics and characteristics

Character	Training set (*n* = 197)	Testing set (*n* = 197)
	No. of patients %	No. of patients %
**Age** (mean, range)	67.9 (45–88)	68.1 (34–90)
**Gender**		
Male	141 (71.6)	149 (75.6)
Female	56 (28.4)	48 (24.4)
**Stage**		
II	62 (31.5)	62 (31.5)
III	57 (28.9)	79 (40.1)
IV	76 (38.6)	56 (28.4)
NA	2 (1.0)	0 (0)

$$\mathrm{AS risk score}=1.2118 \times (\mathrm{PSI of ISLR}\_\mathrm{AP}\_31676)+1.4092\times (\mathrm{PSI of C}1\mathrm{QTNF}6\_\mathrm{AP}\_62089)+1.6065\times (\mathrm{PSI of COX}6\mathrm{C}\_\mathrm{AT}\_84676)+2.3912\times (\mathrm{PSI of WRAP}53\_\mathrm{AP}\_39043)+0.9139\times (\mathrm{PSI of MORN}1\_\mathrm{AT}\_252)+1.9795\times (\mathrm{PSI of ARHGEF}16\_\mathrm{AP}\_309)+1.2453\times (\mathrm{PSI of ARID}5\mathrm{B}\_\mathrm{AP}\_11865)+0.3885\times (\mathrm{PSI of MYH}11\_\mathrm{ES}\_34188)+(-1.5430)\times (\mathrm{PSI of GTF}2\mathrm{IRD}2\_\mathrm{AT}\_80091)+1.2702\times (\mathrm{PSI of CA}5\mathrm{B}\_\mathrm{ES}\_98313)+(-0.8304)\times (\mathrm{PSI of MAST}1\_\mathrm{AT}\_47879)+(-0.4760)\times (\mathrm{PSI of AHCYL}2\_\mathrm{AP}\_81744)+(-0.5180)\times (\mathrm{PSI of APBB}3\_\mathrm{AD}\_73680)+(-1.8810)\times (\mathrm{PSI of C}19\mathrm{ or of }57\_\mathrm{ES}\_47943)+(-0.2698)\times (\mathrm{PSI of SYT}8\_\mathrm{RI}\_13859)$$

**Fig. 6:**
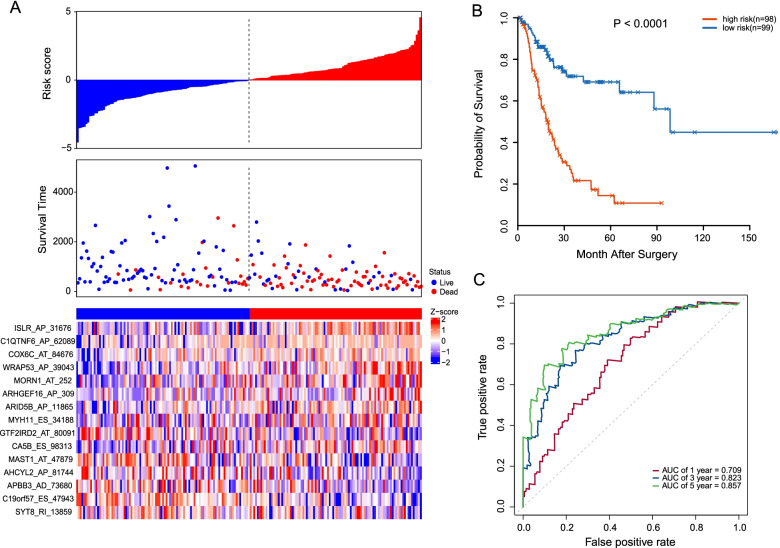
Identification of a 15-AS signature significantly associated with the OS of MIBC patients in TCGA training set. **A** The distribution of risk score, survival status, and the PSI pattern of 15-AS signature. The upper panel indicates the curve of risk score distribution, middle panel indicates the survival time and status of patients with MIBC, and the lower panel is a heatmap which shows the normalized z-score of PSI values for 15-AS signature. Red indicates higher PSI, and blue indicates lower PSI. **D** Kaplan–Meier plots of the prognostic predictors for high- and low-risk groups with MIBC. **E** The time-dependent ROC curves analyses for evaluating the accuracy of the risk scores

The patients with MIBC in the training set were stratified into high- (*n* = 98) and low-risk groups (*n* = 99) according to the median value of risk score. The risk score distribution, OS status, and the PSI value of 15 AS events were shown in Fig. 6A. Obviously, the high PSI levels of ISLR_AP_31676, C1QTNF6_AP_62089, COX6C_AT_84676, WRAP53_AP_39043, MORN1_AT_252, ARHGEF16_AP_309, ARID5B_AP_11865, and MYH11_ES_34188 were associated with the high-risk group. On the other hand, the levels of GTF2IRD2_AT_80091, CA5B_ES_98313, MAST1_AT_47879, AHCYL2_AP_81744, APBB3_AD_73680, C19orf57_ES_47943, and SYT8_RI_13859 were related to a low-risk group. Kaplan–Meier curve revealed that the patients in the low-risk group had a remarkably overall survival benefit than that in the high-risk group (median time = 1.64 years vs. 1.27 years, *P* < 0.0001, Fig. 6B). The area under the ROC curves (AUCs) for 1-, 3-, and 5-year OS predictions were 0.709, 0.823, and 0.857 (Fig. 6C). All these results indicated that the 15 prognostic AS events have a highly predictive performance.

### Validation of AS-15 signature in the test set

**Fig. 7:**
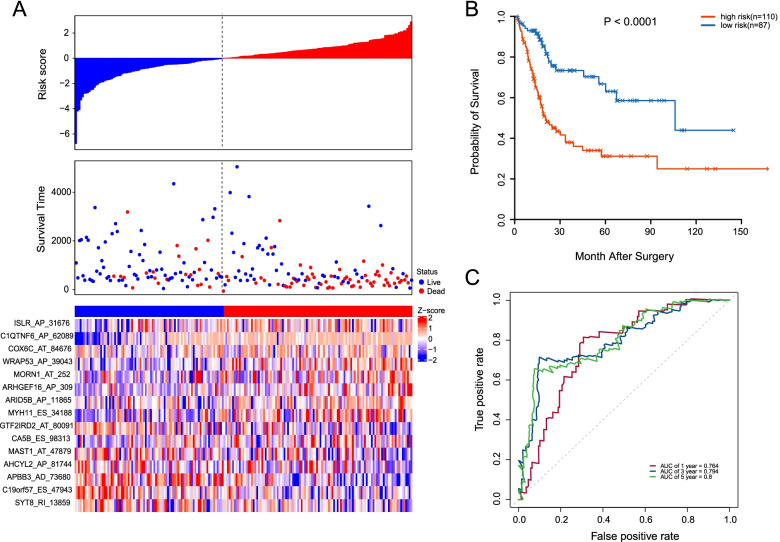
Identification of a 15-AS signature significantly associated with the OS of MIBC patients in TCGA testing set. **A** The distribution of risk score, survival status, and the PSI pattern of 15-AS signature. The upper panel indicates the curve of risk score distribution, middle panel indicates the survival time and status of patients with MIBC, and the lower panel is a heatmap which shows the normalized z-score of PSI values for 15-AS signature. Red indicates higher PSI, and blue indicates lower PSI. **D** Kaplan–Meier plots of the prognostic predictors for high- and low-risk groups with MIBC. **E** The time-dependent ROC curves analyses for evaluating the accuracy of the risk scores

Furthermore, an independent test set (*n* = 197) was employed to validate the predictive efficacy of the 15-AS signature. With the same formula and risk score cutoff in the training set, patients in the test set could be classified into high- (*n* = 110) and low-risk (*n* = 87) groups. The risk score distribution, OS status, and the PSI value of AS events were analogous to those in the training set (Fig. 7A). Moreover, patients with low-risk scores had a notable longer OS than that in the high-risk scores group (median time = 1.78 years vs. 1.31 years, *P* < 0.0001, Fig. 7B). The AUCs for 1-, 3-, and 5-year OS predictions for the risk scores were 0.764, 0.794, and 0.8, respectively, which yielded similar results as in the training set (Fig. 7C). Collectively, the validation in test set confirmed that the 15-AS signature could be a robust and reliable prognostic predictor for MIBC patients.

### The AS-15 signature is an independent prognostic predictor for MIBC

A multivariable Cox regression analysis was further conducted to determine whether the AS-15 signature was an independent prognostic factor for MIBC. In the total TCGA set (*n* = 394), after adjusted for well-established factors, such as the AJCC stage and patient age, the AS-15 marker remained significantly associated with OS (adjusted *P*-value < 0.0001, hazard ratio (HR) = 1.36). These results demonstrated that the AS-15 signature was a highly reliable independent prognostic factor for MIBC.

### Gene set enrichment analysis (GSEA) identifies 15-AS signature-related signaling pathways

**Fig. 8:**
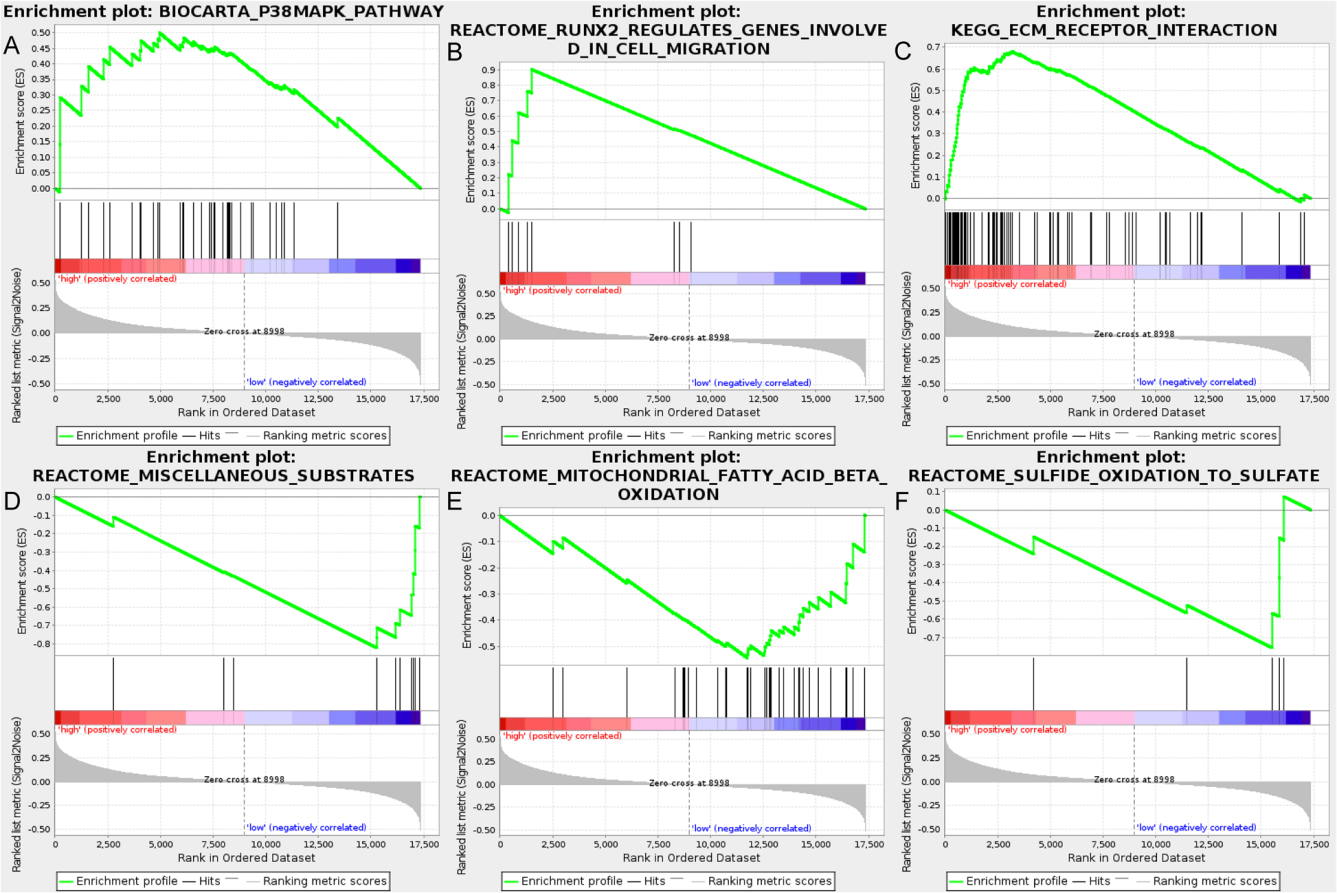
The GSEA analysis results in TCGA MIBC. **A** “P38-MAPK pathway.” **B** “Runx2 regulates genes involved in cell migration.” **C** “ECM receptor interaction.” **D** “Miscellaneous substrates.” **E** “Mitochondrial fatty acid beta oxidation.” **F** “Sulfide oxidation to sulfate”

To investigate the signaling pathways associated with the 15-AS signature, GSEA was employed to explore the gene sets that were differentially enriched between the high- and low-risk groups. GSEA results revealed that the gene sets in the high-risk group were closely related to the process that stimulates tumor proliferation and metastasis including P38-MAPK pathway (*NES* = 1.81, *P* = 0), Runx2 regulates genes involved in cell migration (*NES* = 1.72, *P* = 0), and ECM receptor interaction (*NES* = 1.70, *P* = 0.002), while gene sets in the low-risk group were associated with metabolism such as mitochondrial fatty acid beta oxidation (*NES* =  − 1.70, *P* = 0.010), miscellaneous substrates (*NES* =  − 1.51, *P* = 0.014), and sulfide oxidation to sulfate (*NES* =  − 1.51, *P* = 0.49). The results of the GSEA are shown in Fig. 8.

### Immune landscape characterizing high-risk and low-risk MIBC patients

**Fig. 9:**
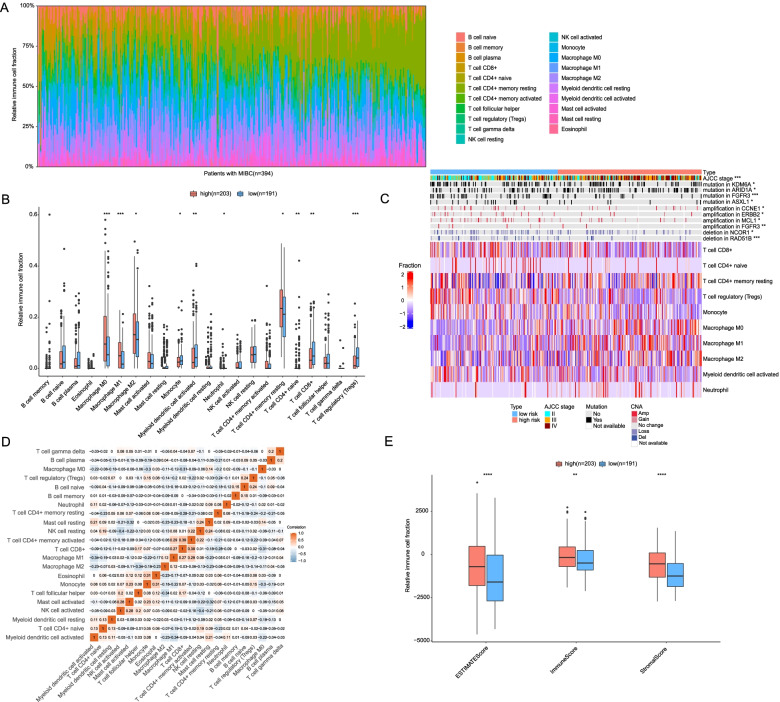
Relationship between 15-AS signature and immune characterization of patients with MIBC in high-risk and low-risk patients. **A** Relative abundances of 22 immune cell types inferred by CIBERSORT algorithm in patients with MIBC. **B** Box plot indicates significantly differential immune cell infiltration status between high- and low-risk patients. **C** Heatmap of significant immune cell types and associated mutations or copy number alternations between high- and low-risk patients. **D** Correlation matrix of relative abundances among the 22 immune cell types. **E** Box plots show differential immune scores/stromal scores/ESTIMATE scores between high- and low-risk patients. *P < 0.05, **P < 0.01, ***P < 0.001, ****P < 0.0001

To further analyze the tumor immune microenvironment was related to 15-AS signature, CIBERSORT was conducted to estimate the composition of 22 distinct infiltrating immune-cell types in the patients with MIBC. The distribution of 22 immune-cell types in each individual was shown in Fig. 9A. Next, the immune infiltrations between the low- and high-risk groups were carefully compared. We observed that low-risk groups had a higher proportion of myeloid dendritic cell activated (*P* = 0.0095), T-cell CD8 + (*P* = 0.0073), T-cell CD4 + naive (*P* = 0.0019), and T-cell regulatory (Tregs) (*P* < 0.001), and monocyte (*P* = 0.027), while high-risk groups had a relatively higher fraction of T-cell CD4 + memory resting (*P* = 0.014), M0 macrophages (*P* < 0.001), M1 macrophages (*P* < 0.001), and macrophage M2 (*P* = 0.044) (Fig. 9B). Moreover, a heatmap of significant infiltrating immune-cell types and driver mutations or copy number alternations that are associated with high- or low-risk groups were investigated. Interestingly, we observed that the prevalence of FGFR3 with both mutations and focal amplification was significantly higher in low-risk group (Fig. 9C). Moreover, the relative abundance of 22 immune-cell types was found to be weakly to moderately correlated (Fig. 9D). The population of M0 macrophages was positively correlated with that of T-cell CD8 + (*r* = 0.31) but inversely correlated monocyte (*r* = 0.3) and myeloid dendritic cell activated (*r* = 0.22). According to the ESTIMATE analysis, a higher estimate score was observed in the low-risk group. Similarly, immune and stromal scores are significantly associated with the low-risk group (Fig. 9E). Collectively, these data indicated that our 15-AS signature could serve as an indicator to evaluate immune status as well as potential targeted therapies in MIBC.

### Construction and validation of nomogram with AS signature

**Fig. 10:**
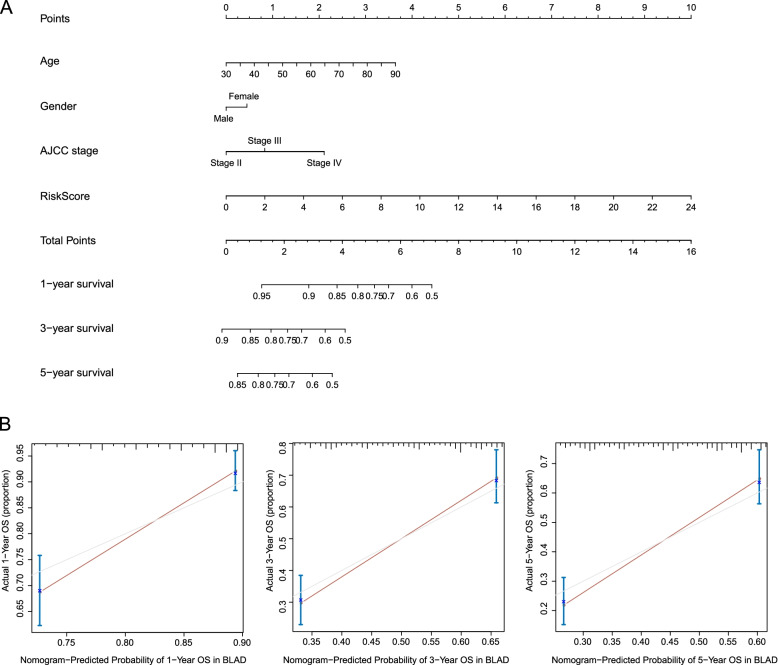
Construction of a nomogram to predict 1-, 3-, or 5-year OS in entire MIBC cohort. **A** A nomogram based on the 15-AS signature risk score and clinical factors. **B** Calibration plot for evaluating the predictive accuracy of the nomogram at 1-, 3-, and 5-year survival

To develop a model to quantitatively predict the individual’s prognosis by intaking AS risk scores and clinicopathologic features, a nomogram was constructed to predict the probability of 1-, 3-, and 5-year survival in the total TCGA MIBC cohorts. Factors such as age, gender, AJCC stage, and AS signature risk scores were included in the nomogram model. As shown in Fig. 10A, each variable was assigned points in proportion to its risk contribution to OS, and the C-index of nomogram model was 0.715. The calibration plots achieved a good consistency between predicted and actual observation probabilities of 1-, 3-, and 5- year survival in Fig. 10B.

### Construction of potential SF-AS regulatory network

**Fig. 11:**
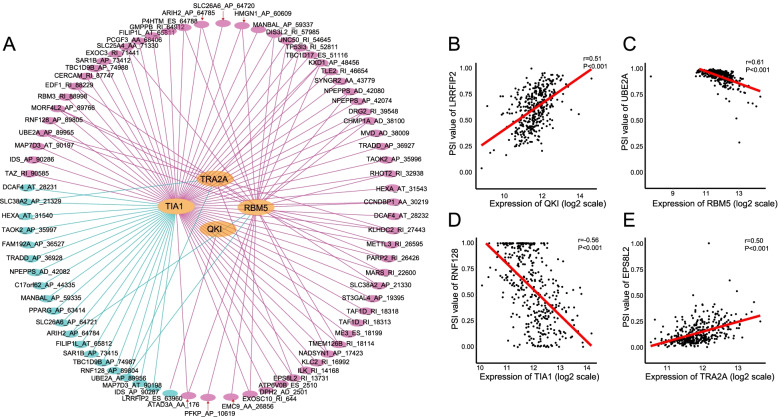
Prognostic SFs and the splicing correlation network of MIBC. **A** Splicing correlation network in patients with MIBC constructed by Cytoscape. AS factors (orange dots) were positively (pink edges) or negatively (green edges) associated with AS events, which predicted favorable (pink dots, *HR* < 1) or adverse (green dots, *HR* > 1) outcomes in patients with MIBC. **B**–**E** A correlation between the expression of AS factor and the PSI value of survival-associated splicing event

It is generally acknowledged that widespread AS events might be extensively regulated by their splicing factors in cancer process; we therefore focused on several key AS factors associated with prognosis and their perturbable regulatory network. Herein, a total of 71 splicing factors (SF) from the SpliceAid2 database were applied to identify prognostic splicing factors. As a result, we obtained 10 SFs whose expression level was strongly associated with OS in MIBC (Supplementary Table 3). In addition, Spearman correlation coefficient was used to assess the association between prognostic SFs and AS events. Only the significant associations with the *P*-value < 0.001 were included in the regulatory network (Fig. 11A). As depicted in the SF-AS regulatory network, AS events of four splicing factors, including transformer 2 alpha homolog (TRA2A), RNA-binding motif 5 (RBM5), quaking (QKI), and TIA1 cytotoxic granule-associated RNA-binding protein (TIA1), were significantly associated with OS (*P* < 0.001). Interestingly, we observed that the majority of critical AS factors (orange dots) were associated with more than one AS event, and a part of them may play opposite roles in regulating different AS events. In addition, we also observed that different splicing factors were strongly associated with the same AS event, for example, splicing factors TIA1 and RBM5 were both significantly correlated with RI of METTL3, which indicated that different splicing factors might regulate the splicing process of one gene. Besides, correlations between splicing factors and their AS events were shown in the scatter plots representatively (Fig. 11 B–E). For example, expression of QKI was positively correlated with ES of LRRFIP2 with *r* = 0.51, *P* < 0.001 (Fig. 11B), whereas RBM5 negatively correlated with AP of UBE2A with *r* =  − 0.61, *P* < 0.001 (Fig. 11C).

## Discussion

AS is a posttranscriptional regulatory process that could generate tremendous transcriptomic diversity and modulate various proteins in human and other eukaryotes. It has become clear that aberrant AS is a major contributor to cancer initiation, progression, and metastasis, especially in colorectal and breast cancer. Tumorigenesis of AS in bladder cancer is a complex multistep process that involves a procedure of aberrant pre-mRNA splicing, assembling into the incorrect protein products, and consequently leads to malignant progression. Several studies have confirmed that the AS events in cancer tissues brought us great promising insights into the cancer-specific mechanisms of non-muscle-invasive bladder cancer. For instance, Kasper et al. identified seven genes with tumor-specific splice variants in bladder cancer, which could represent general cancer-related splicing events [22]. Pamela et al. [23] found that splicing factor PTBP1 could promote expression of oncogenic splice events and result in adverse prognosis. Nevertheless, these studies only focused on single genes, and comprehensive analyses of AS signatures in MIBC lag far behind.

Our study aims to perform systematic analyses of prognostic alternative splicing events in 394 MIBC patients from The Cancer Genome Atlas (TCGA) project and provide a comprehensive picture of RNA splicing patterns in MIBC. We detected a total of 2589 alternative splicing events from 1732 parent genes which were significantly associated with OS in MIBC. Of these, ES events were the predominant frequent type, accounted for approximately one-third of all of the AS types, followed by AP and AT events, which was consistent with other previous studies. Additionally, the parent genes of AS events we identified include CDK10, TP53, MAP4K3, and ERBB2IP, which played important role in cancer initial and progress [24, 25]. Particularly, the biological function of AT in TP53 has not been investigated so far. TP53-AT splicing mode might be helpful to unravel its oncogenesis mechanism in MIBC. Besides, to explore the underlying biological functions for prognostic AS events in MIBC, GO pathway analyses suggested that prognostic AS events can be mediated by RNA spliceosomal complex and DNA-directed RNA polymerase II, core complex pathways, which was regarded as the key regulator in RNA splicing process [26, 27]. The KEGG enrichment analyses also revealed several significant perturbed pathways, including lysosome, and base excision repair, which played pivotal roles in multiple cancer-related processes [28].

Then, we established a 15-AS prognostic signature in training set by LASSO and multivariate Cox regression analysis. We divided MIBC patients into high- and low-risk groups by the median risk score and observed that patients with higher risk scores had significantly shorter OS. Moreover, the 15-AS signature was also validated in test set, indicating a good reproducibility in MIBC. Meanwhile, we found that 15-AS signature is an independent prognostic predictor which considered other clinicopathological factors in multivariate Cox regression model. Collectively, these results suggested that this 15-AS signature served as a prognostic biomarker that could be applied in clinical in the further. Besides, GSEA analysis revealed that high-risk group tended to be participated in several processes of stimulating tumor proliferation and metastasis. For instance, P38-MAPK pathway and Runx2 regulate genes involved in cell migration pathway affecting cancer progression and migration. However, metabolism pathways were enriched in low-risk group, like mitochondrial fatty acid beta oxidation and sulfide oxidation to sulfate. Another interesting finding was that those groups have distinct tumor immune environment. High-risk group might be considered into immunologically “hot” tumors, from which T-cell CD8 + , T-cell CD4 + naïve, and myeloid dendritic cell activated were significantly elevated; however, the abundance of T-cell CD4 + memory resting, M0 macrophages, and M2 macrophages was obviously decreased when compared with the high-risk group. Moreover, we observed that the presence of FGFR3 mutations and high-level focal amplification was strongly enriched in the low-risk group. A pan-FGFR inhibitor named erdafitinib [29] received accelerated approval from the US Food and Drug Administration (FDA) for the treatment of adult patients who have aberrant FGFR3 or FGFR2 alterations in locally advanced or metastatic urothelial carcinoma (mUC). A subgroup of patients based on 15-AS signature could help to refine risk stratification systems and associate with potential molecular characteristics of MIBC which might guide clinical treatment decision-making. Additionally, we have established a nomogram integrating age, gender, AJCC stage, and risk scores to predict individual prognosis. This nomogram model could achieve an accurate predictive value for the patients when considering the C-index of 0.715 in the TCGA MIBC set.

The SF-AS regulatory network between prognostic SFs and the most significantly prognostic-associated AS events was further investigated. With the convincing data, we observed that the expression of TIA1 was negatively correlated with adverse prognosis AS events, whereas TRA2A was positively correlated with favorable AS events, which was concordant with OS of these splicing factors. Moreover, we found that expression of TIA1 had a strong correlation with RNF128 AP (*r* =  − 0.56, *P* < 0.001, Fig. 11D). It was reported that RNF128 functions as an E3 ligase and involved in promoting invasion and metastasis in several types of cancer [30, 31]. Hence, it might be reasonable that TIA1 could affect survival via the regulation of aberrant alternative splicing of RNF128 pre-mRNA. Additionally, we also noticed that TRA2A was significantly correlated with EPS8L2 RI (*r* = 0.50, *P* < 0.001, Fig. 11E). EPS8L2, which belongs to EPS8 protein family, is thought to link growth factor stimulation to actin cytoskeletal remodeling. Accumulating studies suggested that EPS8L2 is an exosome-secreted protein and could be used as a potential diagnostic biomarker for bladder cancer [32–34]. Our SF-AS network revealed the association of aberrant alternative splicing of RI in EPS8L2 with splicing factors and provided invaluable clues to uncover potential AS prognostic biomarker for MIBC. Altogether, our findings present a better understanding of the underlying mechanism of splicing factors, which could eventually help to elucidate the pathogenesis of alternative splicing in MIBC.

In summary, our study provides a comprehensive understanding of aberrant alternative splice events to evaluate the prognostic outcomes in MIBC. Although our study was well analyzed, there are several limitations need to be considered. Firstly, patients collected in our cohort were only from TCGA database; validation by other independent cohort was lacking. Second, due to the incomplete clinical information, the relationship between splice events and clinical features in MIBC was not well addressed. Thirdly, our alternative splicing events analysis is based on silico analysis; validation of biomarkers needs to be further explored.

## Conclusions

In conclusion, we presented a systematic approach for prognostic splicing variants in MIBC with 15-AS events signature. In addition, the candidate AS events identified in our study, especially the 15-AS events, were taken into the final signature, which consisted of the most valuable AS events in deciphering the underlying mechanism in MIBC and possessed great potential in clinical implications as biomarkers and therapeutic targets for MIBC patients.

## Supplementary Information

**Additional file 1:** **Supplementary Figure 1.**

**Additional file 2:** **Supplementary Figure 2.**

**Additional file 3:** **Supplementary Table 1.** 2589 prognosis-associated AS events in MIBC. **Supplementary Table 2.** GO and KEGG pathway analysis of prognosis-related AS events by DAVID (https://david.ncifcrf.gov/) website. **Supplementary Table 3.** Prognosis-associated splicing factors.

## Data Availability

The data used to support the findings of this study were obtained from and can be found at the TCGA (https://tcga-data.nci.nih.gov/tcga/) and the SpliceSeq (http://bioinformatics.mdanderson.org/TCGASpliceSeq).
