# Supplementary figures and images for: Alternative splicing patterns reveal prognostic indicator in muscle-invasive bladder cancer
Source: World J Surg Oncol. 2022 Jul 12;20:231. doi: 10.1186/s12957-022-02685-0 (PMC9277948; doi:10.1186/s12957-022-02685-0)

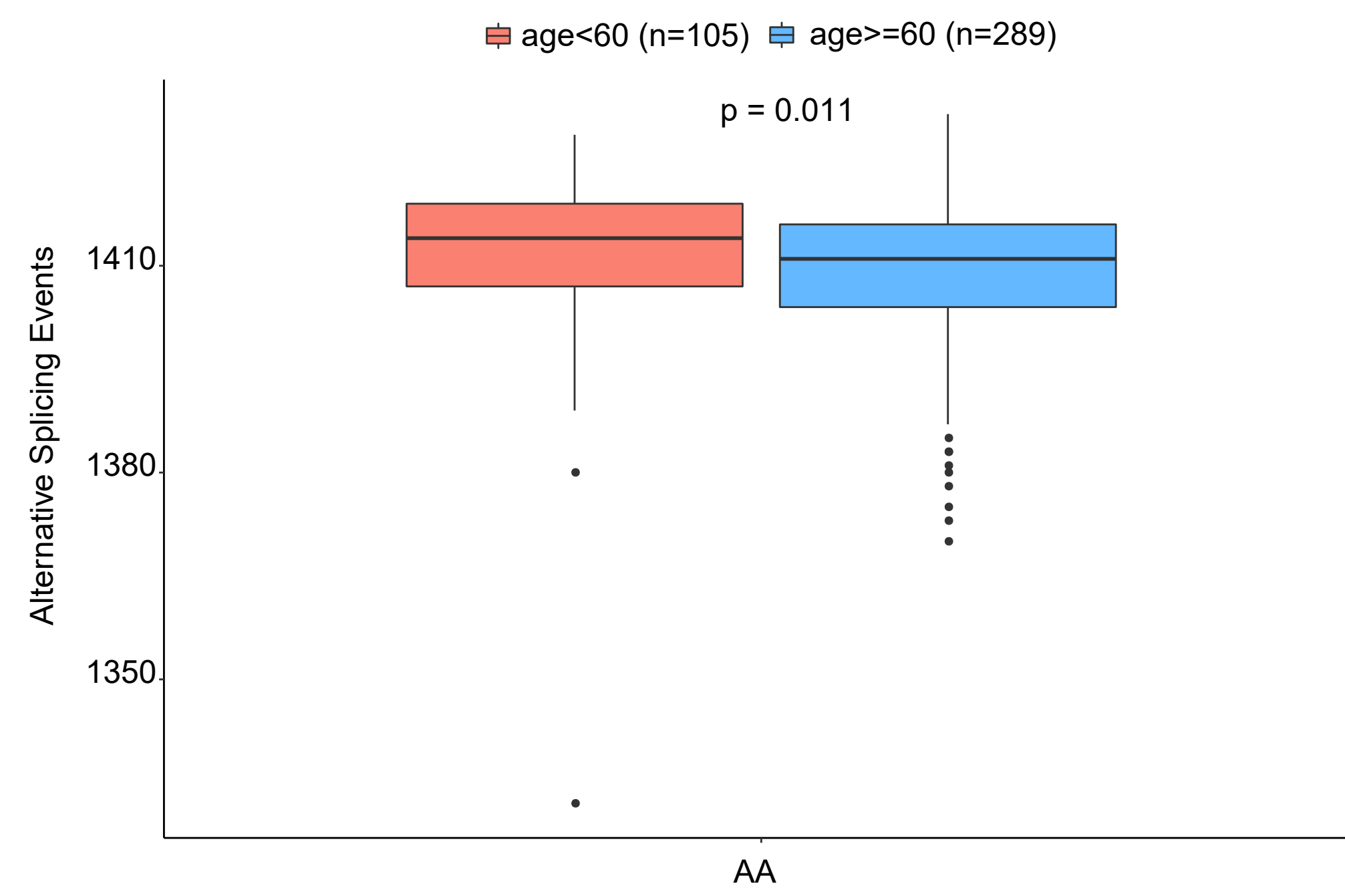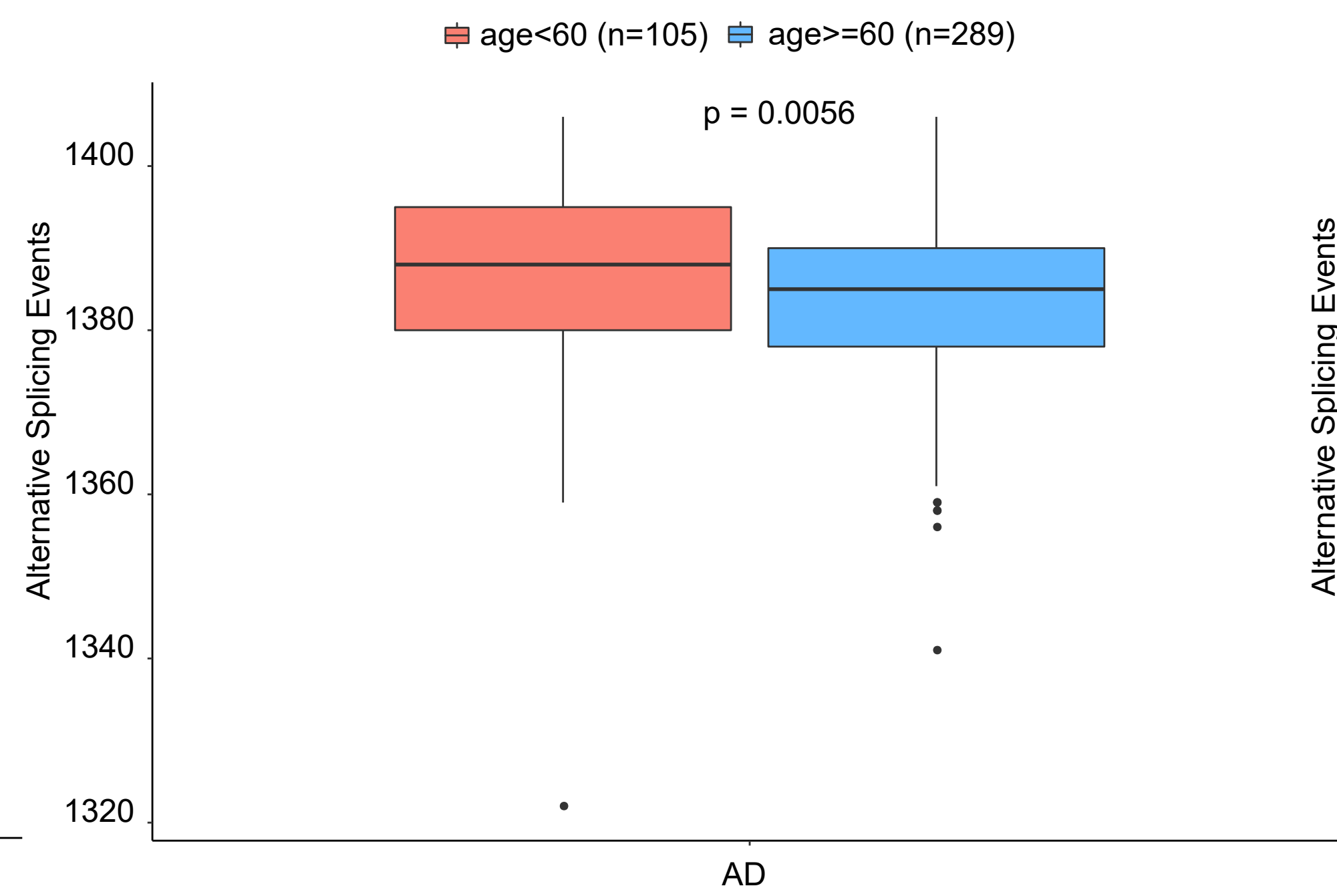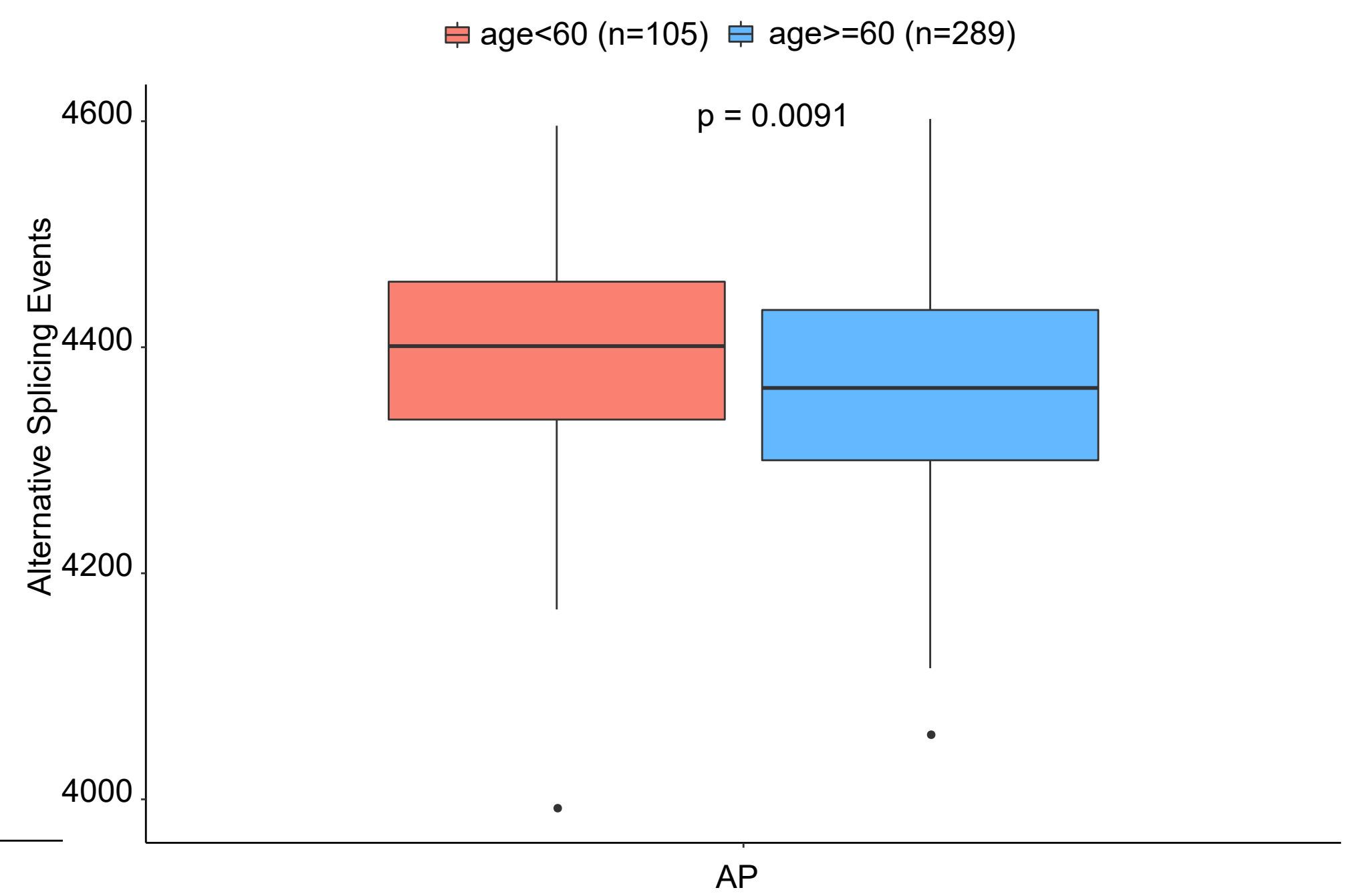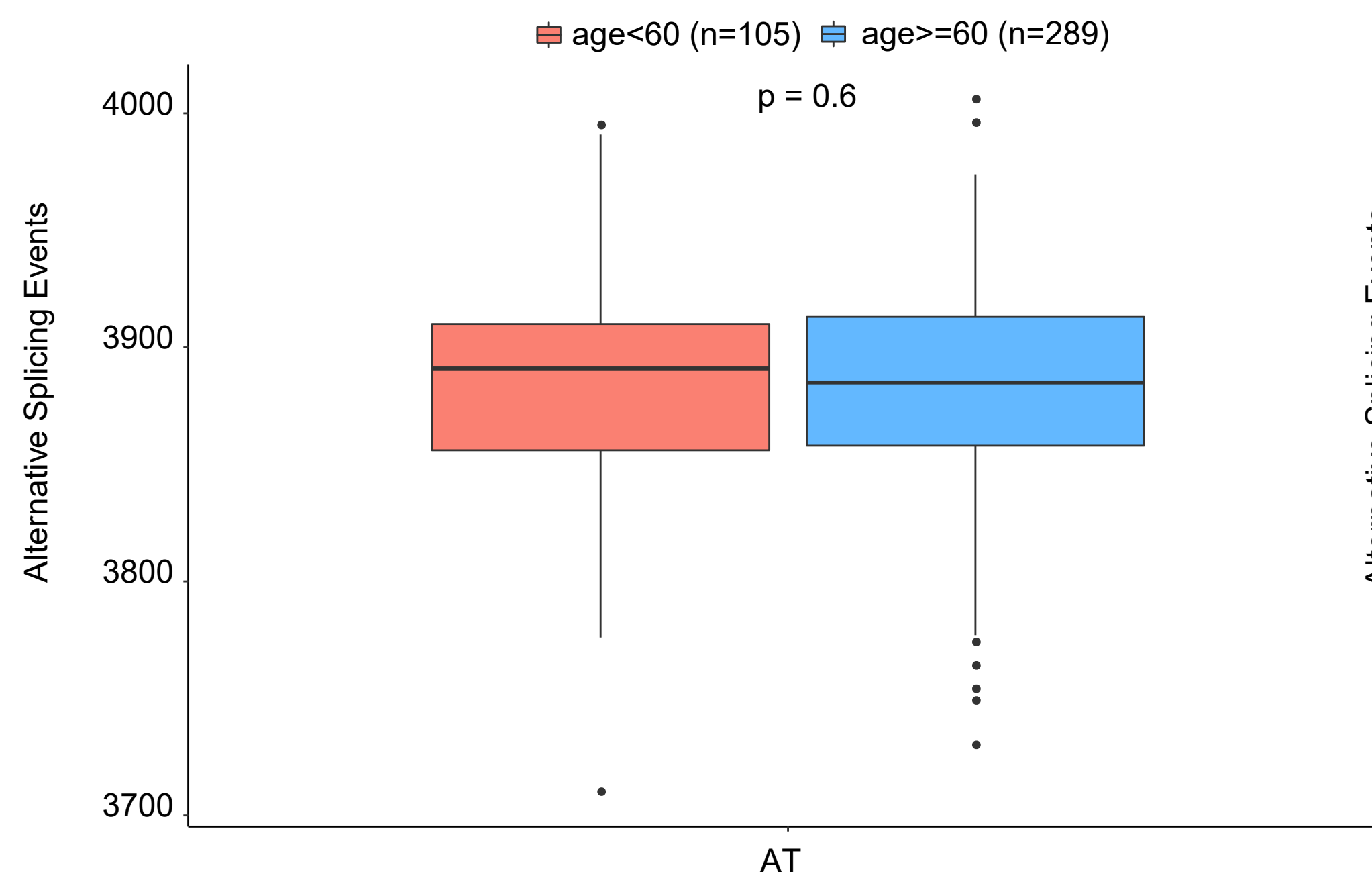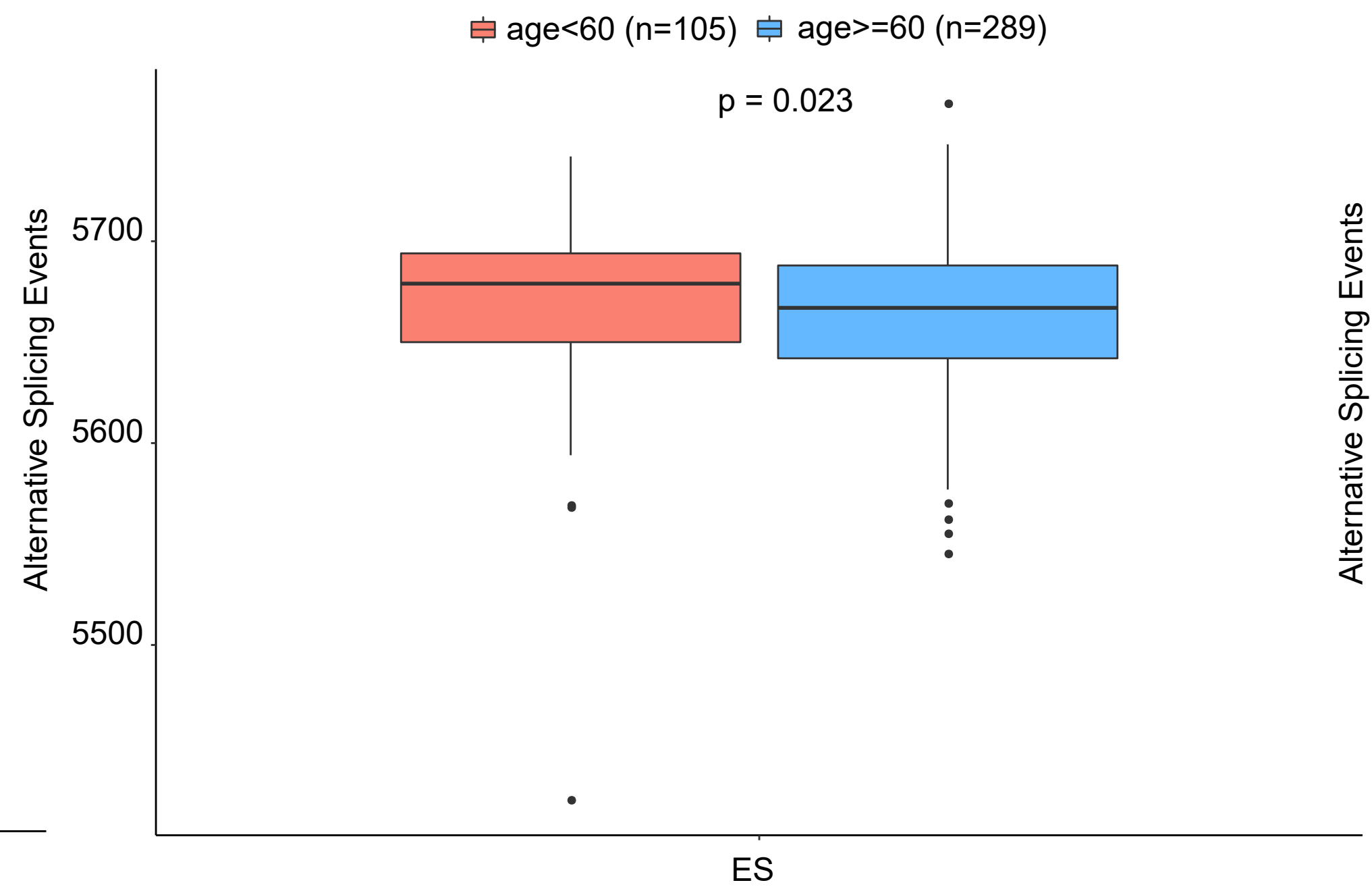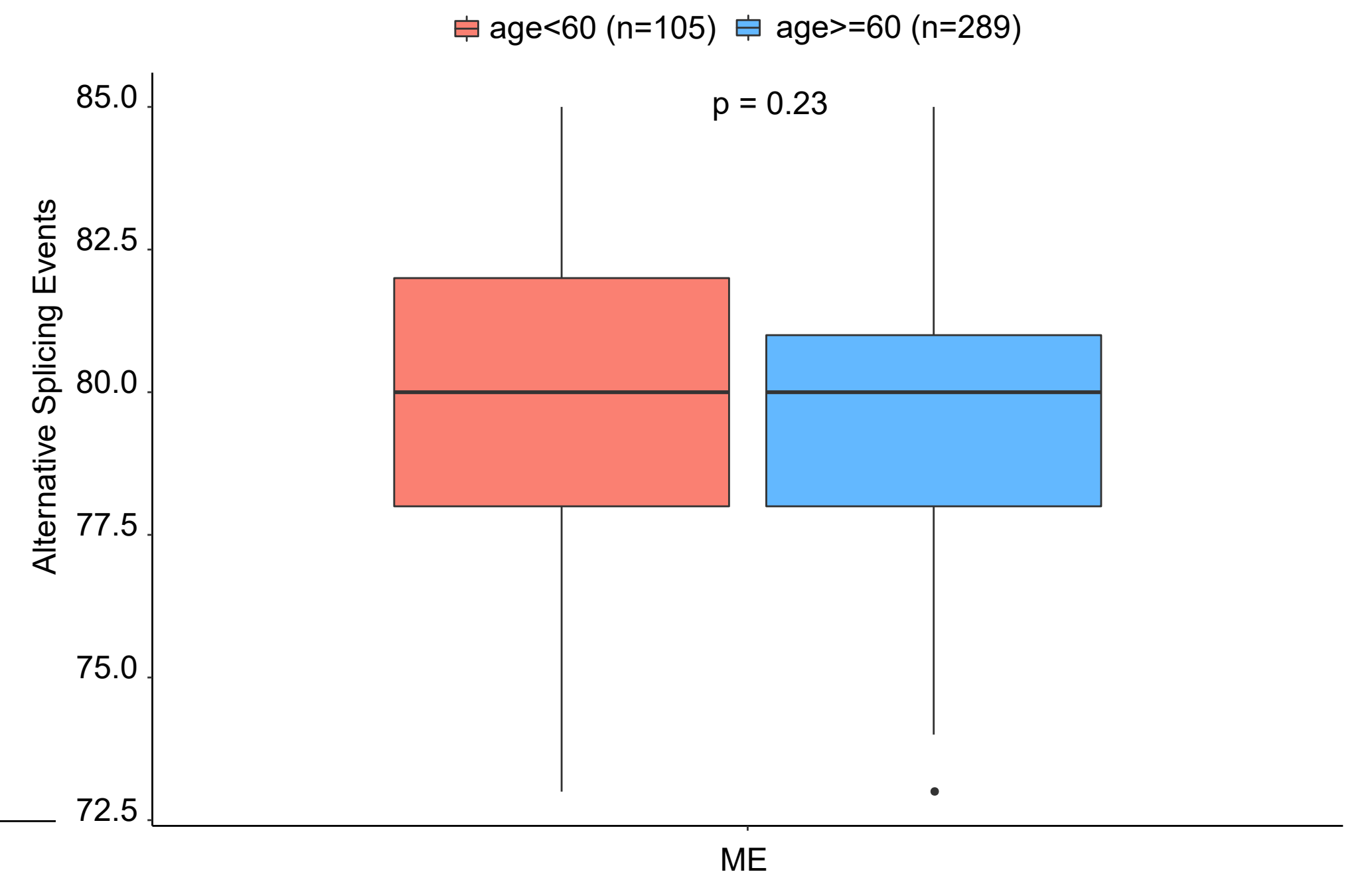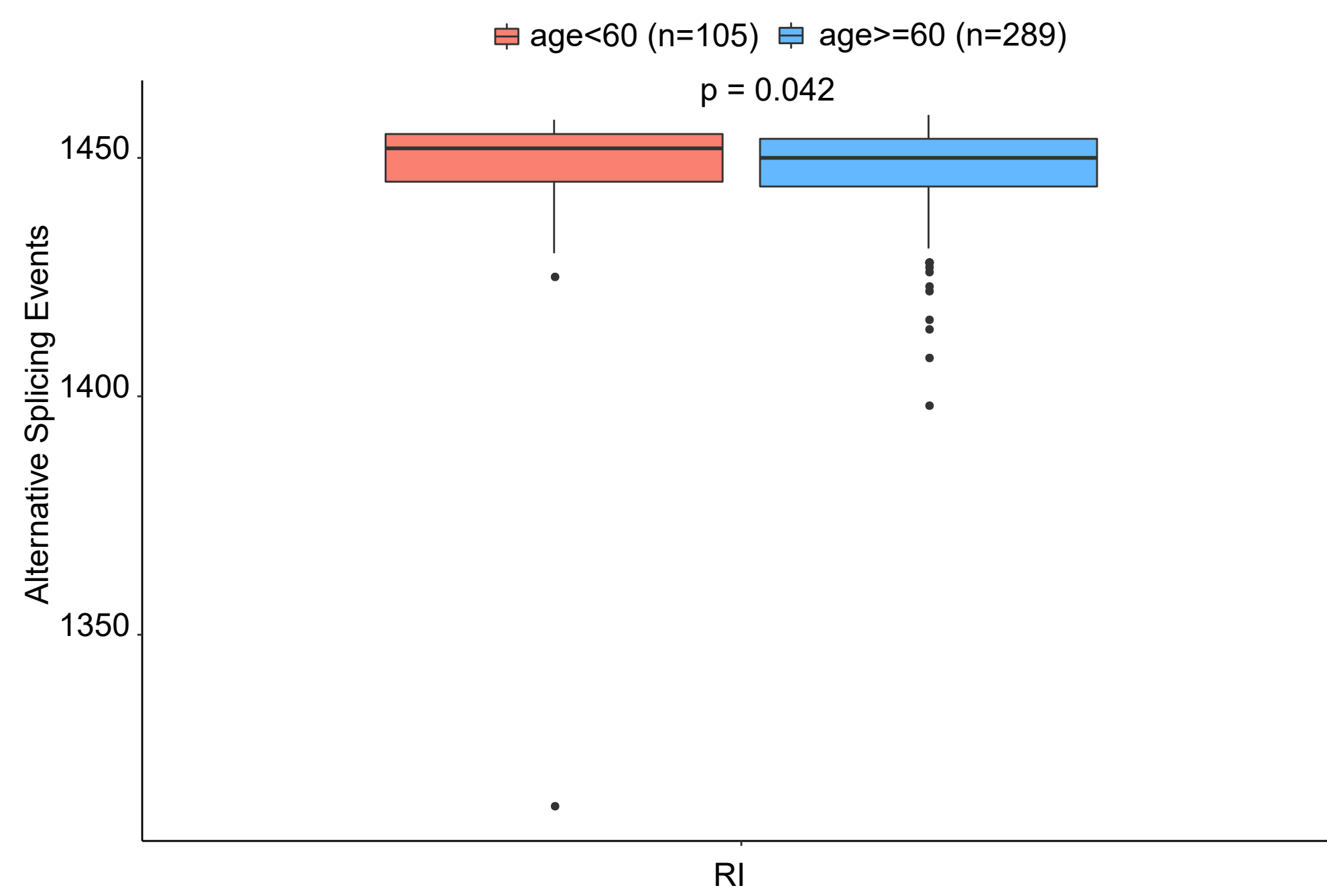

Supplement: Supplementary file 1 — Additional file 1: Supplementary Figure 1. [file 12957_2022_2685_MOESM1_ESM.pdf]

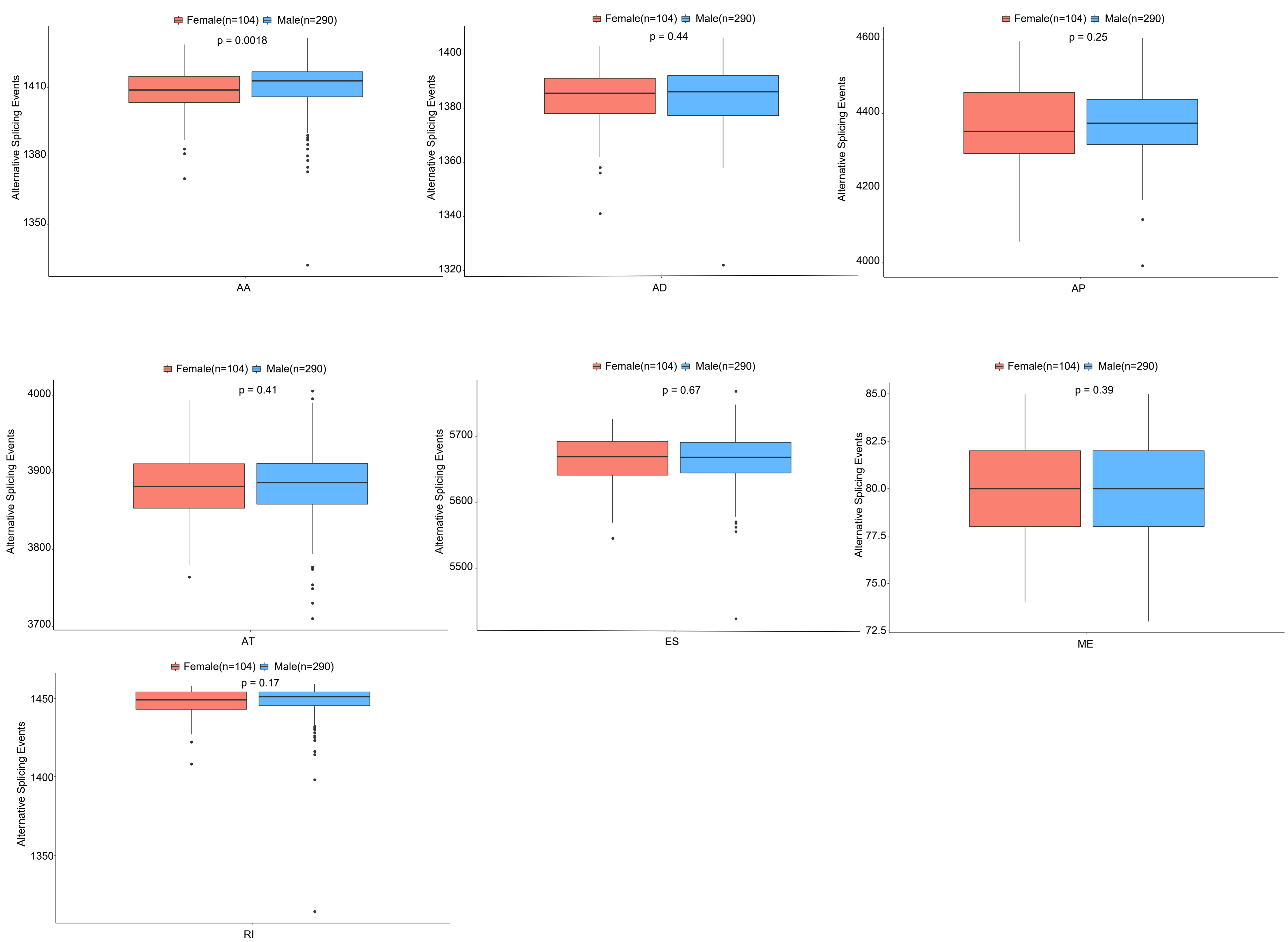

Supplement: Supplementary file 2 — Additional file 2: Supplementary Figure 2. [file 12957_2022_2685_MOESM2_ESM.pdf]
