# Supplementary material for: Alternative splicing patterns reveal prognostic indicator in muscle-invasive bladder cancer
Source: World J Surg Oncol. 2022 Jul 12;20:231. doi: 10.1186/s12957-022-02685-0 (PMC9277948; doi:10.1186/s12957-022-02685-0)
Supplement: Supplementary file 3 — Additional file 3: Supplementary Table 1. 2589 prognosis-associated AS events in MIBC. Supplementary Table 2. GO and KEGG pathway analysis of prognosis-related AS events by DAVID (https://david.ncifcrf.gov/) website. Supplementary Table 3. Prognosis-associated splicing factors. [file 12957_2022_2685_MOESM3_ESM.docx]

**Supplementary Table 1: 2589 prognosis-associated AS events in MIBC.** This table shows the prognosis-associated AS events in MIBC with log-rank p value <0.05. In each column, Gene AS events represents detailed prognosis-associated AS events; Hazard Ratio(HR) represents the ratio of (risk of outcome in one group)/(risk of outcome in another group), occurring at a given interval of time ; 95%CI represents reliable range of values( 95% confidence interval) in which we expect the true population parameter to be included ; P value measures the statistical significance in log-rank test.

| Gene AS events | Hazard Ratio (HR) | 95%CI | Log-rank P-value |
| --- | --- | --- | --- |
| TMEM126B_RI_18114 | 0.49 | 0.36-0.67 | 3.60E-06 |
| PFDN5_ES_21997 | 0.49 | 0.36-0.67 | 4.79E-06 |
| SIDT2_RI_18888 | 0.50 | 0.37-0.68 | 7.41E-06 |
| ZNF439_AP_47755 | 0.51 | 0.37-0.69 | 1.12E-05 |
| MMAB_ES_24322 | 0.51 | 0.37-0.69 | 1.17E-05 |
| P4HTM_ES_64788 | 0.52 | 0.38-0.70 | 1.65E-05 |
| AKNAD1_AT_3952 | 0.52 | 0.38-0.70 | 2.01E-05 |
| LSP1_AP_13868 | 1.90 | 1.40-2.58 | 2.95E-05 |
| HINFP_AA_19122 | 0.53 | 0.39-0.71 | 3.25E-05 |
| DNAJC17_ES_30047 | 0.53 | 0.39-0.72 | 3.53E-05 |
| IDS_AP_90286 | 0.53 | 0.39-0.72 | 3.60E-05 |
| IDS_AP_90287 | 1.89 | 1.39-2.57 | 3.60E-05 |
| AK9_AT_77203 | 1.89 | 1.39-2.57 | 3.91E-05 |
| GSTM1_ES_4070 | 1.88 | 1.38-2.55 | 4.00E-05 |
| HPCAL1_ES_52658 | 0.53 | 0.39-0.72 | 4.11E-05 |
| OLFML2B_AP_8735 | 0.53 | 0.39-0.72 | 4.33E-05 |
| RHOT2_RI_32938 | 0.53 | 0.39-0.72 | 4.41E-05 |
| PCGF2_AP_40586 | 0.53 | 0.39-0.73 | 4.56E-05 |
| OLFML2B_AP_8734 | 1.87 | 1.38-2.54 | 4.77E-05 |
| PCGF2_AP_40584 | 1.87 | 1.38-2.53 | 4.78E-05 |
| NPEPPS_AP_42075 | 1.86 | 1.38-2.53 | 4.89E-05 |
| EPS8L2_RI_13731 | 0.54 | 0.40-0.73 | 4.97E-05 |
| SPIN2A_AP_89297 | 0.54 | 0.39-0.73 | 5.48E-05 |
| NPEPPS_AD_42082 | 1.86 | 1.37-2.52 | 5.66E-05 |
| PTGER3_AT_3412 | 1.87 | 1.37-2.55 | 5.68E-05 |
| SP140L_RI_57889 | 0.54 | 0.40-0.73 | 6.30E-05 |
| NPEPPS_AP_42074 | 0.54 | 0.40-0.74 | 6.92E-05 |
| PFKP_AP_10619 | 0.54 | 0.40-0.74 | 7.39E-05 |
| RNF128_AP_89805 | 0.55 | 0.40-0.74 | 8.39E-05 |
| CIRBP_RI_46432 | 0.55 | 0.40-0.74 | 8.79E-05 |
| MTFR1L_AA_1212 | 0.55 | 0.40-0.74 | 9.16E-05 |
| SAR1B_AP_73415 | 1.83 | 1.34-2.49 | 9.25E-05 |
| SPIN2A_AP_89298 | 1.83 | 1.34-2.49 | 9.30E-05 |
| MUTYH_AA_2671 | 1.82 | 1.34-2.47 | 9.57E-05 |
| SLC38A2_AP_21329 | 1.82 | 1.34-2.46 | 9.62E-05 |
| SLC38A2_AP_21330 | 0.55 | 0.41-0.74 | 9.62E-05 |
| DYM_ES_45472 | 0.55 | 0.40-0.75 | 9.81E-05 |
| LSM14A_ES_48955 | 0.55 | 0.40-0.75 | 9.84E-05 |
| TBC1D9B_AP_74987 | 1.82 | 1.34-2.47 | 9.91E-05 |
| C17orf62_AP_44335 | 1.82 | 1.34-2.46 | 0.000102468 |
| GMPPB_RI_64912 | 0.55 | 0.41-0.75 | 0.000109213 |
| SLC26A6_AP_64721 | 1.81 | 1.33-2.44 | 0.000113386 |
| SHC4_AP_30507 | 1.81 | 1.33-2.46 | 0.000113458 |
| CBWD5_AT_86498 | 0.55 | 0.41-0.75 | 0.000120502 |
| MARS_RI_22600 | 0.55 | 0.41-0.75 | 0.000123038 |
| TPM2_ES_98132 | 0.56 | 0.41-0.75 | 0.000135705 |
| SYNGR2_AA_43779 | 0.56 | 0.41-0.76 | 0.000136335 |
| AHI1_ES_77894 | 0.56 | 0.41-0.75 | 0.000137242 |
| SMUG1_AP_22120 | 1.80 | 1.32-2.44 | 0.000142655 |
| CCNDBP1_AA_30219 | 0.56 | 0.41-0.76 | 0.00015577 |
| SLC26A6_AP_64720 | 0.56 | 0.41-0.76 | 0.000159043 |
| KLHDC2_RI_27443 | 0.56 | 0.41-0.76 | 0.000167893 |
| SRP19_AA_72988 | 0.56 | 0.41-0.76 | 0.000175222 |
| CARM1_RI_47597 | 0.56 | 0.41-0.76 | 0.000176955 |
| LRRFIP2_ES_63960 | 1.78 | 1.31-2.41 | 0.000182389 |
| SHC4_AP_30508 | 0.56 | 0.41-0.76 | 0.000186141 |
| SH3BP2_ES_68600 | 0.56 | 0.41-0.76 | 0.000187152 |
| EMC9_AA_26856 | 0.56 | 0.42-0.76 | 0.000189811 |
| EML2_ES_50500 | 0.56 | 0.42-0.76 | 0.000190291 |
| ANK3_AP_11842 | 0.56 | 0.41-0.76 | 0.000196086 |
| APBB3_RI_73673 | 0.56 | 0.42-0.77 | 0.00019624 |
| SUOX_ES_22342 | 0.56 | 0.42-0.77 | 0.000198273 |
| FLNB_ES_65418 | 0.57 | 0.42-0.77 | 0.000220368 |
| RPL28_AT_52095 | 1.76 | 1.30-2.39 | 0.000227305 |
| TRADD_AP_36927 | 0.57 | 0.42-0.77 | 0.000227628 |
| TRADD_AP_36928 | 1.76 | 1.30-2.38 | 0.000227628 |
| KTN1_AP_27628 | 1.76 | 1.30-2.39 | 0.000231032 |
| DIS3L2_RI_57985 | 0.57 | 0.42-0.77 | 0.000237972 |
| MTMR14_AP_63102 | 1.76 | 1.30-2.38 | 0.000240807 |
| MTMR14_AP_63104 | 0.57 | 0.42-0.77 | 0.000240807 |
| KLC2_RI_16992 | 0.57 | 0.42-0.77 | 0.000249592 |
| UBP1_ES_63866 | 0.57 | 0.42-0.77 | 0.000255674 |
| TBC1D9B_AP_74988 | 0.57 | 0.42-0.77 | 0.000257462 |
| B3GAT3_AA_16372 | 0.57 | 0.42-0.77 | 0.000259161 |
| UBE2A_AP_89955 | 0.57 | 0.42-0.77 | 0.000263895 |
| UBE2A_AP_89956 | 1.75 | 1.29-2.37 | 0.000263895 |
| PCGF3_AA_68406 | 0.57 | 0.42-0.77 | 0.000269065 |
| MANBAL_AP_59335 | 1.75 | 1.29-2.37 | 0.000270983 |
| ATP6V0B_ES_2510 | 0.57 | 0.42-0.78 | 0.000275528 |
| TARBP2_AA_22073 | 0.57 | 0.42-0.78 | 0.000279945 |
| MAP7D3_AT_90197 | 0.57 | 0.42-0.78 | 0.000287396 |
| MAP7D3_AT_90198 | 1.74 | 1.29-2.36 | 0.000287396 |
| MAP4K3_ES_53330 | 0.57 | 0.42-0.78 | 0.000294743 |
| TAF1D_RI_18318 | 0.57 | 0.42-0.78 | 0.000296623 |
| C12orf49_AP_24657 | 0.57 | 0.42-0.78 | 0.000297876 |
| FBXO44_ES_658 | 0.57 | 0.42-0.78 | 0.000303131 |
| HEXA_AT_31540 | 1.74 | 1.28-2.35 | 0.000305108 |
| ACTG1_RI_44120 | 0.58 | 0.42-0.78 | 0.000315962 |
| AGAP3_ES_82353 | 0.58 | 0.42-0.78 | 0.000316149 |
| DCAF4_AT_28232 | 0.58 | 0.42-0.78 | 0.00032182 |
| PARP2_RI_26426 | 0.58 | 0.43-0.78 | 0.000323418 |
| KDELC2_AP_18615 | 1.73 | 1.28-2.35 | 0.000328128 |
| KYNU_AT_55479 | 1.74 | 1.28-2.35 | 0.000331809 |
| MVD_AD_38009 | 0.58 | 0.43-0.78 | 0.000339517 |
| HNRNPF_AP_11324 | 0.58 | 0.42-0.78 | 0.000340776 |
| PHLDB2_AP_66057 | 0.58 | 0.42-0.78 | 0.000352417 |
| DUOXA1_ES_30390 | 0.58 | 0.43-0.78 | 0.000363965 |
| NSMCE4A_AP_13328 | 1.73 | 1.27-2.34 | 0.000367801 |
| NSMCE4A_AP_13329 | 0.58 | 0.43-0.78 | 0.000367801 |
| EBPL_AT_25899 | 1.73 | 1.27-2.35 | 0.000372705 |
| EBPL_AT_25900 | 0.58 | 0.43-0.79 | 0.000372705 |
| ZNF439_AP_47756 | 1.73 | 1.27-2.35 | 0.000374288 |
| WDR62_RI_49341 | 0.58 | 0.43-0.79 | 0.000377798 |
| EDF1_RI_88229 | 0.58 | 0.43-0.79 | 0.000380687 |
| DCAF4_AT_28231 | 1.73 | 1.27-2.34 | 0.000381141 |
| ST3GAL4_AP_19391 | 1.73 | 1.27-2.36 | 0.000386215 |
| FHL2_ES_54832 | 0.58 | 0.43-0.79 | 0.000389853 |
| ST3GAL4_AP_19395 | 0.58 | 0.43-0.79 | 0.000405553 |
| AP1S3_AT_57760 | 1.72 | 1.27-2.33 | 0.000406617 |
| POLR2J3_RI_81116 | 0.58 | 0.43-0.79 | 0.000408411 |
| CPAMD8_AT_48218 | 0.58 | 0.43-0.79 | 0.000421123 |
| ARHGEF1_ES_50101 | 0.58 | 0.43-0.79 | 0.000422806 |
| EXOC3_RI_71441 | 0.58 | 0.43-0.79 | 0.000428336 |
| MANBAL_AP_59337 | 0.58 | 0.43-0.79 | 0.000428992 |
| SZRD1_ES_811 | 0.58 | 0.43-0.79 | 0.000433036 |
| CPAMD8_AT_48219 | 1.71 | 1.26-2.33 | 0.000456525 |
| TAOK2_AP_35996 | 0.58 | 0.43-0.79 | 0.000460105 |
| TAOK2_AP_35997 | 1.71 | 1.26-2.32 | 0.000460105 |
| GBA2_AA_86291 | 0.58 | 0.43-0.79 | 0.0004669 |
| SAR1B_AP_73412 | 0.58 | 0.43-0.79 | 0.000468622 |
| RNF128_AP_89804 | 1.71 | 1.26-2.32 | 0.00048405 |
| NADSYN1_AP_17423 | 0.59 | 0.43-0.79 | 0.000485059 |
| MRC2_AA_42911 | 0.59 | 0.43-0.79 | 0.000485717 |
| C12orf49_AP_24658 | 1.71 | 1.26-2.31 | 0.000490157 |
| ALDH5A1_ES_75516 | 1.71 | 1.26-2.31 | 0.000496248 |
| TXLNA_AP_1559 | 1.70 | 1.26-2.31 | 0.000505054 |
| RBM3_RI_88998 | 0.59 | 0.43-0.80 | 0.000506863 |
| MAZ_AP_35937 | 1.70 | 1.26-2.31 | 0.000510755 |
| ABCB9_AP_24995 | 1.71 | 1.26-2.31 | 0.000511037 |
| RBP1_AT_67032 | 0.59 | 0.43-0.80 | 0.000511686 |
| FILIP1L_AT_65811 | 0.59 | 0.43-0.80 | 0.000513682 |
| HEXA_AT_31543 | 0.59 | 0.43-0.80 | 0.000525373 |
| TAZ_RI_90585 | 0.59 | 0.43-0.80 | 0.00054558 |
| BMP1_ES_82988 | 0.59 | 0.43-0.80 | 0.000545603 |
| UBE3B_AA_24313 | 0.59 | 0.43-0.80 | 0.00054869 |
| TP53_AT_39036 | 0.59 | 0.44-0.80 | 0.000549967 |
| RNF166_AP_38013 | 0.59 | 0.43-0.80 | 0.000550654 |
| DCTN3_RI_86182 | 0.59 | 0.44-0.80 | 0.00055933 |
| RNF166_AP_38014 | 1.69 | 1.25-2.29 | 0.000563228 |
| PIK3C3_AP_45311 | 0.59 | 0.43-0.80 | 0.000564453 |
| RRM1_AP_14033 | 0.59 | 0.43-0.80 | 0.000564747 |
| ZBTB25_AT_27882 | 0.59 | 0.44-0.80 | 0.000566943 |
| ZBTB25_AT_27883 | 1.70 | 1.25-2.30 | 0.000566943 |
| ILK_RI_14168 | 0.59 | 0.44-0.80 | 0.000576286 |
| FAM192A_AP_36527 | 1.69 | 1.25-2.30 | 0.000584785 |
| TBC1D17_ES_51116 | 0.59 | 0.43-0.80 | 0.000588905 |
| GUK1_ES_10183 | 0.59 | 0.44-0.80 | 0.000601679 |
| TP53I3_RI_52811 | 0.59 | 0.44-0.80 | 0.000610256 |
| MTFR1L_ES_1213 | 0.59 | 0.44-0.80 | 0.000617319 |
| SLC11A2_AT_21729 | 0.59 | 0.44-0.80 | 0.000626377 |
| SLC11A2_AT_21730 | 1.69 | 1.25-2.29 | 0.000626377 |
| UNC50_RI_54645 | 0.59 | 0.44-0.80 | 0.000634292 |
| YBEY_ES_60914 | 1.69 | 1.25-2.28 | 0.00063465 |
| ALKBH3_ES_15467 | 1.69 | 1.25-2.29 | 0.000639095 |
| METTL3_RI_26595 | 0.59 | 0.44-0.80 | 0.000653725 |
| NARF_ES_44399 | 0.59 | 0.44-0.80 | 0.000669606 |
| PLGLB1_AT_54460 | 0.59 | 0.44-0.80 | 0.000672023 |
| MAT2B_RI_74453 | 0.59 | 0.44-0.80 | 0.000672421 |
| KALRN_AT_66523 | 1.69 | 1.24-2.29 | 0.000672705 |
| CERS5_ES_21684 | 0.59 | 0.44-0.80 | 0.000673802 |
| KTN1_AP_27626 | 0.59 | 0.44-0.80 | 0.000674814 |
| EXOSC10_RI_644 | 0.59 | 0.44-0.80 | 0.000687438 |
| CHMP1A_AD_38100 | 0.59 | 0.44-0.80 | 0.000688744 |
| IL32_AD_33439 | 0.59 | 0.44-0.81 | 0.000702019 |
| AHCYL2_AP_81744 | 0.59 | 0.44-0.80 | 0.000702367 |
| PPARG_AP_63414 | 1.68 | 1.24-2.28 | 0.000708952 |
| DPH2_AD_2501 | 0.60 | 0.44-0.81 | 0.000713894 |
| SCLY_AT_58198 | 0.60 | 0.44-0.81 | 0.000727047 |
| SCLY_AT_58199 | 1.68 | 1.24-2.27 | 0.000727047 |
| RRP8_AA_14162 | 0.60 | 0.44-0.81 | 0.000732791 |
| ATG16L2_AT_17649 | 0.59 | 0.44-0.81 | 0.000735596 |
| ATG16L2_AT_17650 | 1.68 | 1.24-2.28 | 0.000735596 |
| KXD1_AP_48456 | 0.60 | 0.44-0.81 | 0.00074109 |
| C17orf62_AP_44336 | 0.60 | 0.44-0.81 | 0.000742358 |
| NPEPPS_AD_42080 | 0.60 | 0.44-0.81 | 0.000754789 |
| AOC3_AP_41156 | 0.60 | 0.44-0.81 | 0.000755063 |
| DDT_AP_61356 | 0.60 | 0.44-0.81 | 0.000760391 |
| SIDT2_RI_18887 | 0.60 | 0.44-0.81 | 0.000763205 |
| ME3_ES_18199 | 0.60 | 0.44-0.81 | 0.00077128 |
| HMGN1_AP_60609 | 0.60 | 0.44-0.81 | 0.000771898 |
| ARIH2_AP_64784 | 1.68 | 1.23-2.27 | 0.000788172 |
| ARIH2_AP_64785 | 0.60 | 0.44-0.81 | 0.000788172 |
| CERCAM_RI_87747 | 0.60 | 0.44-0.81 | 0.000791077 |
| MORF4L2_AP_89766 | 0.60 | 0.44-0.81 | 0.000793992 |
| TBX1_AT_61081 | 1.67 | 1.24-2.27 | 0.000799553 |
| TAF1D_RI_18313 | 0.60 | 0.44-0.81 | 0.000805534 |
| PPFIBP2_AP_14200 | 0.60 | 0.44-0.81 | 0.000806312 |
| PPFIBP2_AP_14203 | 1.67 | 1.23-2.27 | 0.000808726 |
| IDE_ES_12534 | 0.60 | 0.44-0.81 | 0.000814098 |
| NPIPB3_AP_34443 | 1.67 | 1.23-2.27 | 0.000823521 |
| ATAD3A_AA_176 | 0.60 | 0.44-0.81 | 0.000831421 |
| LRRD1_AT_80421 | 1.67 | 1.23-2.26 | 0.00083621 |
| LRRD1_AT_80422 | 0.60 | 0.44-0.81 | 0.00083621 |
| C1QTNF6_AP_62089 | 1.67 | 1.23-2.26 | 0.000858466 |
| C9orf89_AA_86900 | 0.60 | 0.44-0.81 | 0.000863004 |
| ARL6IP4_AA_25028 | 0.60 | 0.44-0.81 | 0.000871196 |
| SLC8B1_AP_24633 | 0.60 | 0.44-0.81 | 0.000874486 |
| SNX29_ES_34058 | 0.60 | 0.44-0.81 | 0.00087721 |
| FUT3_ES_46944 | 0.60 | 0.44-0.81 | 0.000902002 |
| RBP1_AT_67033 | 1.66 | 1.23-2.25 | 0.000923616 |
| NHEJ1_ES_57591 | 0.60 | 0.44-0.81 | 0.000923634 |
| DUSP18_AT_61794 | 1.67 | 1.23-2.26 | 0.000928826 |
| DCAKD_AP_41927 | 1.66 | 1.23-2.26 | 0.000929747 |
| PCGF3_ES_68407 | 0.60 | 0.44-0.82 | 0.000936389 |
| SLC25A4_AA_71330 | 0.60 | 0.45-0.82 | 0.000946149 |
| DRG2_RI_39548 | 0.60 | 0.45-0.82 | 0.000958673 |
| LMF2_AA_62836 | 0.60 | 0.44-0.82 | 0.000962561 |
| RHOT1_ES_40176 | 0.60 | 0.44-0.82 | 0.00096692 |
| TLE2_RI_46654 | 0.60 | 0.45-0.82 | 0.000988202 |
| MLPH_ES_58115 | 1.66 | 1.22-2.24 | 0.000988663 |
| FILIP1L_AT_65812 | 1.66 | 1.22-2.25 | 0.000997425 |
| SUSD4_AT_9911 | 0.60 | 0.45-0.82 | 0.001003324 |
| CLIP1_AP_24943 | 0.60 | 0.45-0.82 | 0.001005399 |
| AP1B1_ES_61603 | 1.66 | 1.22-2.24 | 0.001007743 |
| VPS28_RI_85606 | 0.60 | 0.45-0.82 | 0.001018741 |
| ARFIP2_ES_14135 | 0.61 | 0.45-0.82 | 0.001021907 |
| TMEM175_ES_68431 | 0.60 | 0.45-0.82 | 0.001026799 |
| TMEM107_ES_39131 | 0.60 | 0.44-0.82 | 0.00103079 |
| NAGK_AA_53917 | 0.61 | 0.45-0.82 | 0.001038325 |
| TCF12_AP_30783 | 1.65 | 1.22-2.24 | 0.001051065 |
| PTGES2_ES_87700 | 0.60 | 0.45-0.82 | 0.001055792 |
| GSS_RI_59053 | 0.61 | 0.45-0.82 | 0.001077347 |
| TPM1_AP_30979 | 1.65 | 1.22-2.24 | 0.001080528 |
| INO80E_ES_36009 | 0.61 | 0.45-0.82 | 0.001090526 |
| VSTM4_AT_11498 | 1.65 | 1.22-2.23 | 0.001097932 |
| VSTM4_AT_11499 | 0.61 | 0.45-0.82 | 0.001097932 |
| SLC9B2_AP_70167 | 0.61 | 0.45-0.82 | 0.001104859 |
| FGF11_AP_38952 | 0.61 | 0.45-0.82 | 0.001105906 |
| TTLL12_AP_62574 | 1.65 | 1.22-2.23 | 0.001110335 |
| TIMMDC1_ES_66322 | 1.65 | 1.22-2.23 | 0.001114753 |
| SLC6A8_AP_90463 | 0.61 | 0.45-0.82 | 0.001114779 |
| TKT_ES_65305 | 0.61 | 0.45-0.82 | 0.001117121 |
| PLCD4_ES_57506 | 0.61 | 0.45-0.82 | 0.001118819 |
| LMNA_AP_8173 | 1.65 | 1.22-2.23 | 0.001121301 |
| MTMR3_ES_61690 | 1.65 | 1.22-2.23 | 0.001122682 |
| ROGDI_ES_33858 | 0.61 | 0.45-0.82 | 0.001124043 |
| CBWD6_AT_86488 | 0.61 | 0.45-0.82 | 0.001139203 |
| PLS3_ES_89927 | 0.61 | 0.45-0.82 | 0.001141069 |
| CDC37_RI_47514 | 0.61 | 0.45-0.82 | 0.001141763 |
| LEPROTL1_AT_83274 | 1.65 | 1.22-2.24 | 0.001146895 |
| TNK2_AA_68210 | 0.61 | 0.45-0.82 | 0.001161661 |
| INO80E_ES_36005 | 0.61 | 0.45-0.82 | 0.001166991 |
| PGM3_AP_76860 | 0.61 | 0.45-0.82 | 0.001168926 |
| CDK2_RI_22325 | 0.61 | 0.45-0.82 | 0.00116987 |
| ERBB2IP_ES_72261 | 0.61 | 0.45-0.82 | 0.00117038 |
| MCF2L_AP_26317 | 1.64 | 1.21-2.22 | 0.001176557 |
| PCSK5_AT_86634 | 1.65 | 1.21-2.24 | 0.001178253 |
| ULK3_RI_31757 | 0.61 | 0.45-0.82 | 0.001190142 |
| SMIM1_AT_337 | 1.64 | 1.21-2.23 | 0.00119457 |
| IL32_AD_33436 | 0.61 | 0.45-0.82 | 0.001195765 |
| NAB1_AP_56578 | 0.61 | 0.45-0.82 | 0.00119872 |
| RBM19_AT_24645 | 1.64 | 1.21-2.22 | 0.001206776 |
| RBM19_AT_24646 | 0.61 | 0.45-0.82 | 0.001206776 |
| PFKP_AP_10621 | 1.64 | 1.21-2.22 | 0.001208309 |
| KALRN_AT_66524 | 0.61 | 0.45-0.82 | 0.001211528 |
| TMEM138_RI_16188 | 0.61 | 0.45-0.82 | 0.001233129 |
| SEC14L2_AT_61736 | 0.61 | 0.45-0.83 | 0.001241704 |
| SEC14L2_AT_61738 | 1.64 | 1.21-2.22 | 0.001241704 |
| ELMO3_AP_36970 | 0.61 | 0.45-0.83 | 0.001244309 |
| AOC3_AP_41155 | 1.64 | 1.21-2.22 | 0.001250625 |
| PTOV1_RI_51096 | 0.61 | 0.45-0.83 | 0.001252485 |
| TPM2_ES_98133 | 0.61 | 0.45-0.83 | 0.001254932 |
| SYNGR2_AA_43775 | 0.61 | 0.45-0.83 | 0.001266625 |
| NADSYN1_AP_17422 | 1.64 | 1.21-2.21 | 0.001268736 |
| AUTS2_AT_79909 | 0.61 | 0.45-0.83 | 0.001282085 |
| AUTS2_AT_79910 | 1.64 | 1.21-2.22 | 0.001282085 |
| MED17_RI_18329 | 0.61 | 0.45-0.83 | 0.001290665 |
| TUBB3_ES_38173 | 0.61 | 0.45-0.83 | 0.001292213 |
| RPE_ES_57242 | 0.61 | 0.45-0.83 | 0.001298078 |
| TMEM143_ES_50751 | 0.61 | 0.45-0.83 | 0.001307559 |
| MRPL10_AP_42097 | 0.61 | 0.45-0.83 | 0.001310356 |
| MRPL10_AP_42098 | 1.64 | 1.21-2.22 | 0.001310356 |
| NAB1_AP_56577 | 1.63 | 1.21-2.21 | 0.001318163 |
| ACRC_AP_89428 | 0.60 | 0.43-0.83 | 0.00131932 |
| C19orf57_ES_47943 | 0.61 | 0.45-0.83 | 0.0013357 |
| ERBB2IP_ES_72263 | 0.61 | 0.45-0.83 | 0.001338379 |
| PCBD2_AT_73438 | 1.63 | 1.21-2.21 | 0.0013443 |
| PCBD2_AT_73439 | 0.61 | 0.45-0.83 | 0.0013443 |
| PAPOLA_ES_29207 | 1.64 | 1.21-2.23 | 0.001348212 |
| NLE1_ES_40281 | 0.61 | 0.45-0.83 | 0.001348542 |
| CWC25_AD_40597 | 0.61 | 0.45-0.83 | 0.001348889 |
| SIRT2_ES_49714 | 0.61 | 0.45-0.83 | 0.001360923 |
| SRSF7_AA_53281 | 0.61 | 0.45-0.83 | 0.001370571 |
| DNAJC4_ES_16600 | 0.61 | 0.45-0.83 | 0.001375756 |
| ZNF564_ES_47805 | 0.61 | 0.45-0.83 | 0.001377903 |
| PPP1R26_AP_88105 | 0.61 | 0.45-0.83 | 0.001378755 |
| CAPN10_ES_58280 | 0.61 | 0.45-0.83 | 0.001380661 |
| LY75_AT_55707 | 1.63 | 1.21-2.21 | 0.00138276 |
| LY75_AT_55708 | 0.61 | 0.45-0.83 | 0.00138276 |
| PRKAR1A_AP_43142 | 0.61 | 0.45-0.83 | 0.001382904 |
| SV2A_AT_7410 | 0.61 | 0.45-0.83 | 0.001392312 |
| SV2A_AT_7411 | 1.63 | 1.21-2.21 | 0.001392312 |
| POLB_AP_83706 | 0.61 | 0.45-0.83 | 0.001411549 |
| POLB_AP_83707 | 1.63 | 1.20-2.21 | 0.001411549 |
| RNF146_ES_77453 | 1.64 | 1.21-2.23 | 0.001421908 |
| NAT9_RI_43292 | 0.61 | 0.45-0.83 | 0.001423987 |
| PQLC1_ES_46266 | 0.61 | 0.45-0.83 | 0.001424982 |
| CARD6_AT_71873 | 1.63 | 1.20-2.21 | 0.001440866 |
| CARD6_AT_71874 | 0.61 | 0.45-0.83 | 0.001440866 |
| PODXL_RI_81821 | 0.61 | 0.45-0.83 | 0.001441366 |
| SREBF2_ES_62464 | 0.61 | 0.45-0.83 | 0.00144477 |
| AP4S1_AT_27099 | 0.61 | 0.45-0.83 | 0.0014599 |
| AP4S1_AT_27100 | 1.63 | 1.20-2.20 | 0.0014599 |
| NSMF_AP_88306 | 0.61 | 0.45-0.83 | 0.00146397 |
| RBM6_AT_64932 | 1.63 | 1.20-2.21 | 0.001466057 |
| RBM6_AT_64934 | 0.61 | 0.45-0.83 | 0.001466057 |
| EXOSC10_RI_645 | 0.61 | 0.45-0.83 | 0.001474336 |
| PCYOX1L_AP_74031 | 1.63 | 1.20-2.20 | 0.001496162 |
| C9orf89_RI_86903 | 0.61 | 0.45-0.83 | 0.001502485 |
| RANBP3_AA_46963 | 0.62 | 0.46-0.83 | 0.001514119 |
| CTSW_RI_16935 | 0.62 | 0.45-0.83 | 0.001522188 |
| RNF166_AP_38015 | 0.61 | 0.45-0.83 | 0.001522401 |
| WDR33_AT_55243 | 0.62 | 0.46-0.83 | 0.00152303 |
| CRYZL1_AA_60459 | 0.61 | 0.45-0.83 | 0.001524294 |
| GULP1_AT_56499 | 0.61 | 0.45-0.83 | 0.001532243 |
| GULP1_AT_56500 | 1.63 | 1.20-2.21 | 0.001532243 |
| ACOXL_AT_54941 | 0.62 | 0.45-0.83 | 0.001537475 |
| MAX_RI_27943 | 0.62 | 0.46-0.83 | 0.00155552 |
| TAZ_ES_90586 | 0.62 | 0.46-0.83 | 0.001573621 |
| RRM1_AP_14034 | 1.62 | 1.20-2.20 | 0.001576696 |
| UBR4_AP_877 | 1.62 | 1.20-2.20 | 0.001578859 |
| INO80E_ES_36011 | 0.62 | 0.45-0.83 | 0.001581089 |
| NEK10_AT_63770 | 1.69 | 1.23-2.31 | 0.001583393 |
| SUPT7L_RI_53041 | 0.62 | 0.46-0.83 | 0.001594774 |
| DCTN6_ES_83284 | 0.62 | 0.46-0.84 | 0.00161731 |
| MXRA7_AP_43608 | 1.62 | 1.20-2.20 | 0.001619767 |
| ZNF706_AP_84733 | 0.62 | 0.46-0.84 | 0.001635412 |
| OGFR_RI_60085 | 0.62 | 0.46-0.84 | 0.001640182 |
| NR2C1_AT_23741 | 0.62 | 0.45-0.84 | 0.001642682 |
| NR2C1_AT_23742 | 1.62 | 1.20-2.20 | 0.001642682 |
| INIP_ES_87253 | 1.62 | 1.20-2.19 | 0.001650543 |
| C16orf58_RI_36278 | 0.62 | 0.46-0.84 | 0.001653368 |
| PRKAR1B_AP_78506 | 0.62 | 0.46-0.84 | 0.001654132 |
| ZC3H7A_AP_34035 | 1.62 | 1.20-2.20 | 0.001655853 |
| ANAPC5_AP_24855 | 1.62 | 1.19-2.19 | 0.001681005 |
| ANAPC5_AP_24859 | 0.62 | 0.46-0.84 | 0.001681005 |
| SLC6A8_AP_90462 | 1.62 | 1.20-2.19 | 0.001681105 |
| MAPK1_AT_61256 | 1.62 | 1.20-2.20 | 0.001684161 |
| MAPK1_AT_61257 | 0.62 | 0.46-0.84 | 0.001684161 |
| LTBP3_AP_16864 | 0.62 | 0.46-0.84 | 0.00168431 |
| CEP290_AP_23607 | 0.62 | 0.46-0.84 | 0.001687329 |
| MICALL2_AA_78572 | 1.62 | 1.19-2.19 | 0.001697028 |
| C11orf73_RI_18185 | 0.62 | 0.46-0.84 | 0.001720493 |
| DDX11_AA_20971 | 0.62 | 0.46-0.84 | 0.001758554 |
| KLHDC4_ES_37958 | 1.62 | 1.19-2.18 | 0.001758811 |
| MPV17_AP_52963 | 0.62 | 0.46-0.84 | 0.001759559 |
| RBFOX2_AA_61988 | 1.61 | 1.19-2.19 | 0.001763971 |
| NSFL1C_ES_58501 | 1.61 | 1.19-2.18 | 0.00177217 |
| MIEN1_RI_40690 | 0.62 | 0.46-0.84 | 0.001773099 |
| CSF1_AA_4086 | 1.61 | 1.19-2.18 | 0.001774798 |
| RCC1_AP_1386 | 0.62 | 0.46-0.84 | 0.001778315 |
| RCC1_AP_1387 | 1.62 | 1.19-2.19 | 0.001778315 |
| ATF2_ES_56063 | 0.62 | 0.46-0.84 | 0.001790702 |
| SAR1B_AP_73414 | 1.62 | 1.19-2.19 | 0.001800172 |
| FBLIM1_AT_772 | 1.62 | 1.19-2.19 | 0.00183646 |
| FBLIM1_AT_773 | 0.62 | 0.46-0.84 | 0.00183646 |
| VAV3_ES_3934 | 0.62 | 0.46-0.84 | 0.001855328 |
| METTL3_RI_26596 | 0.62 | 0.46-0.84 | 0.001858415 |
| CLK1_AP_56752 | 0.62 | 0.46-0.84 | 0.001866917 |
| TIAL1_ES_13268 | 0.62 | 0.46-0.84 | 0.001885371 |
| TRMT10A_AP_70055 | 0.62 | 0.46-0.84 | 0.001886982 |
| SUMO3_AT_60835 | 1.61 | 1.19-2.18 | 0.001896262 |
| SHISA5_AP_64685 | 1.61 | 1.19-2.18 | 0.001908166 |
| METTL17_AA_26479 | 0.62 | 0.46-0.84 | 0.001915117 |
| NAT9_RI_43289 | 0.62 | 0.46-0.84 | 0.001915911 |
| EMC10_ES_51201 | 0.62 | 0.46-0.84 | 0.00192239 |
| NADK_AP_225 | 1.61 | 1.19-2.17 | 0.001926505 |
| NCOR2_ES_25144 | 0.62 | 0.46-0.84 | 0.001939373 |
| TLE2_RI_46656 | 0.62 | 0.46-0.84 | 0.001961366 |
| ANK3_AP_11845 | 1.61 | 1.19-2.18 | 0.001963798 |
| SLC25A29_AA_29256 | 0.62 | 0.46-0.84 | 0.00197521 |
| P4HA3_AT_17767 | 1.61 | 1.19-2.18 | 0.001975567 |
| RPP38_ES_91486 | 1.61 | 1.19-2.18 | 0.001992463 |
| GHDC_AA_41025 | 0.62 | 0.46-0.84 | 0.002012492 |
| JTB_RI_7776 | 0.62 | 0.46-0.84 | 0.002012763 |
| PRC1_ES_32529 | 1.60 | 1.19-2.17 | 0.002013544 |
| PDCD6IP_AA_63891 | 0.62 | 0.46-0.84 | 0.002023987 |
| SDR39U1_AP_27004 | 1.60 | 1.19-2.17 | 0.002032284 |
| PACS2_AP_29630 | 1.60 | 1.18-2.17 | 0.002035846 |
| EIF5_AP_29441 | 0.62 | 0.46-0.84 | 0.00204863 |
| DYNC2LI1_ES_53403 | 0.62 | 0.46-0.84 | 0.002079113 |
| YES1_AP_44477 | 1.60 | 1.18-2.17 | 0.002083436 |
| GDPD5_AP_17859 | 0.62 | 0.46-0.84 | 0.002084065 |
| ZNF771_AT_36107 | 1.61 | 1.18-2.18 | 0.002085754 |
| FAM175A_AT_69800 | 1.60 | 1.18-2.17 | 0.002110054 |
| INO80C_AA_45175 | 0.62 | 0.46-0.85 | 0.002111557 |
| PAM16_AP_33645 | 0.62 | 0.46-0.84 | 0.002112184 |
| ACTR10_ES_27682 | 0.63 | 0.46-0.85 | 0.002125655 |
| WDR11_RI_13288 | 0.63 | 0.46-0.84 | 0.002153491 |
| C12orf76_AT_24404 | 0.63 | 0.46-0.85 | 0.002158658 |
| LRRC29_AP_36980 | 0.63 | 0.46-0.85 | 0.002171363 |
| ATP2A3_ES_38516 | 0.62 | 0.46-0.85 | 0.002172705 |
| NPRL2_RI_65034 | 0.63 | 0.46-0.85 | 0.002179121 |
| ACOT8_AA_59624 | 0.62 | 0.46-0.85 | 0.00218133 |
| CIRBP_AD_46429 | 0.62 | 0.46-0.85 | 0.002194095 |
| C21orf58_AP_60922 | 0.63 | 0.46-0.85 | 0.002197786 |
| EIF2A_ES_67287 | 0.63 | 0.46-0.85 | 0.002216154 |
| AGAP3_AP_82348 | 1.60 | 1.18-2.16 | 0.002218304 |
| PSMD7_ES_37562 | 0.63 | 0.46-0.85 | 0.002239269 |
| GALK1_RI_43496 | 0.63 | 0.46-0.85 | 0.002265429 |
| ATP8B2_AT_7833 | 0.63 | 0.46-0.85 | 0.0022677 |
| CBWD3_AT_86510 | 1.60 | 1.18-2.16 | 0.002275868 |
| TP53_AT_39037 | 1.59 | 1.18-2.15 | 0.002288535 |
| SIRT2_AA_49713 | 0.63 | 0.46-0.85 | 0.002289483 |
| TMEM138_AT_16186 | 1.60 | 1.18-2.16 | 0.002300496 |
| TMEM138_AT_16187 | 0.63 | 0.46-0.85 | 0.002300496 |
| CD7_RI_44315 | 0.63 | 0.46-0.85 | 0.002328827 |
| BRCC3_AA_90678 | 0.63 | 0.46-0.85 | 0.002336615 |
| WDR55_RI_73717 | 0.63 | 0.46-0.85 | 0.002338156 |
| PHLDB2_AP_66059 | 1.60 | 1.18-2.17 | 0.002344079 |
| NDRG2_RI_26503 | 0.63 | 0.46-0.85 | 0.002353707 |
| CABP1_AP_24774 | 1.59 | 1.18-2.15 | 0.002356492 |
| FKBP5_AT_75918 | 0.63 | 0.46-0.85 | 0.002364176 |
| FKBP5_AT_75919 | 1.59 | 1.18-2.16 | 0.002364176 |
| MRPL55_ES_10121 | 1.59 | 1.18-2.16 | 0.002386803 |
| ANXA8L2_ES_11459 | 0.63 | 0.47-0.85 | 0.002421847 |
| KDELC2_AP_18614 | 0.63 | 0.47-0.85 | 0.0024288 |
| OSTC_ES_70305 | 0.63 | 0.46-0.85 | 0.002440473 |
| TGIF1_AP_44502 | 1.59 | 1.17-2.15 | 0.002480953 |
| FAM76A_ES_1344 | 0.63 | 0.46-0.85 | 0.002483937 |
| GGA3_RI_43399 | 0.63 | 0.47-0.85 | 0.002486139 |
| ISLR_AP_31676 | 1.59 | 1.17-2.15 | 0.00248962 |
| TMEM104_ME_217418 | 0.63 | 0.47-0.85 | 0.002517817 |
| METTL17_RI_26478 | 0.63 | 0.47-0.85 | 0.002558493 |
| TMEM107_ES_39125 | 0.63 | 0.47-0.85 | 0.002582 |
| PSMC3IP_AD_41077 | 0.63 | 0.47-0.85 | 0.002594209 |
| CYP4F12_RI_48110 | 0.63 | 0.47-0.85 | 0.002621318 |
| ABTB1_AP_66615 | 1.58 | 1.17-2.14 | 0.002641564 |
| SLC25A39_AA_41846 | 0.63 | 0.47-0.85 | 0.002656481 |
| ABTB1_AP_66616 | 0.63 | 0.47-0.85 | 0.002658461 |
| CBWD3_AT_86509 | 0.63 | 0.46-0.85 | 0.002661319 |
| SLC25A19_AP_43430 | 0.63 | 0.47-0.85 | 0.002683855 |
| THAP3_AT_486 | 0.63 | 0.47-0.85 | 0.002698287 |
| THAP3_AT_487 | 1.58 | 1.17-2.15 | 0.002698287 |
| APOL2_ES_62014 | 0.63 | 0.46-0.85 | 0.002705112 |
| EFS_ES_26738 | 0.63 | 0.47-0.86 | 0.002717999 |
| TRMT10A_AP_70054 | 1.58 | 1.17-2.14 | 0.002736635 |
| ATP2C1_AP_66756 | 1.59 | 1.17-2.15 | 0.002752772 |
| ELMO3_AP_36971 | 1.58 | 1.17-2.14 | 0.002772714 |
| LTBP3_AP_16862 | 1.58 | 1.17-2.14 | 0.002871549 |
| CTPS2_AP_88576 | 1.58 | 1.17-2.13 | 0.002899824 |
| OGDH_AT_79543 | 0.63 | 0.47-0.86 | 0.00290109 |
| OGDH_AT_79544 | 1.58 | 1.17-2.13 | 0.00290109 |
| AFMID_ES_43801 | 0.63 | 0.47-0.86 | 0.002947046 |
| SLC25A19_AP_43428 | 1.57 | 1.16-2.13 | 0.002973023 |
| MS4A4A_ES_16081 | 0.63 | 0.47-0.86 | 0.002979778 |
| CDH23_AT_12058 | 1.58 | 1.16-2.13 | 0.002983793 |
| HNRNPF_AP_11326 | 0.64 | 0.47-0.86 | 0.002988954 |
| OTUD5_AP_89042 | 0.63 | 0.47-0.86 | 0.003000369 |
| KDM2B_AP_24871 | 1.58 | 1.16-2.13 | 0.00303887 |
| NFATC2IP_AP_35900 | 1.57 | 1.16-2.13 | 0.003040036 |
| TBC1D10A_ES_61719 | 0.64 | 0.47-0.86 | 0.003067586 |
| SMPD4_ES_95699 | 1.57 | 1.16-2.13 | 0.003076538 |
| HARS2_AD_73743 | 0.64 | 0.47-0.86 | 0.003085241 |
| SRGAP1_AP_22855 | 1.57 | 1.16-2.12 | 0.003110083 |
| LDLRAD3_ES_15419 | 0.64 | 0.47-0.86 | 0.003132925 |
| GSDMB_ES_40792 | 0.64 | 0.47-0.86 | 0.003152902 |
| DACT3_AT_50576 | 0.64 | 0.47-0.86 | 0.003153839 |
| DACT3_AT_50577 | 1.57 | 1.16-2.13 | 0.003153839 |
| STARD3_AA_40659 | 0.64 | 0.47-0.86 | 0.003166889 |
| TMEM108_AT_66827 | 1.58 | 1.16-2.14 | 0.003177442 |
| TMEM108_AT_66828 | 0.63 | 0.47-0.86 | 0.003177442 |
| GNPDA1_AP_73857 | 1.57 | 1.16-2.12 | 0.003180205 |
| MARCH6_AP_71560 | 0.64 | 0.47-0.86 | 0.003183601 |
| MARCH6_AP_71561 | 1.57 | 1.16-2.12 | 0.003183601 |
| AP1S3_AT_57757 | 0.64 | 0.47-0.86 | 0.003198906 |
| CTTN_ES_17403 | 0.64 | 0.47-0.86 | 0.003199772 |
| PSMC5_AA_43010 | 0.64 | 0.47-0.86 | 0.00320145 |
| POLR2G_AD_16418 | 0.64 | 0.47-0.86 | 0.003217843 |
| UGT1A1_AP_58049 | 0.64 | 0.47-0.86 | 0.003232148 |
| EHBP1L1_AP_16875 | 1.57 | 1.16-2.12 | 0.003239585 |
| EHBP1L1_AP_16876 | 0.64 | 0.47-0.86 | 0.003239585 |
| METTL5_RI_55890 | 0.64 | 0.47-0.86 | 0.003249947 |
| CCDC24_ES_2530 | 0.64 | 0.47-0.86 | 0.003249959 |
| HIRA_AP_61047 | 1.57 | 1.16-2.12 | 0.003250105 |
| S100A4_AP_7711 | 0.64 | 0.47-0.86 | 0.003253258 |
| SPTAN1_AD_87771 | 0.64 | 0.47-0.86 | 0.003256752 |
| ATMIN_AP_37749 | 0.64 | 0.47-0.86 | 0.00326131 |
| SHROOM1_ES_73312 | 0.64 | 0.47-0.86 | 0.003265889 |
| OGFR_AP_60083 | 0.64 | 0.47-0.86 | 0.00326698 |
| OGFR_AP_60084 | 1.57 | 1.16-2.12 | 0.00326698 |
| MRPL55_AA_10102 | 0.64 | 0.47-0.86 | 0.003267932 |
| HMBS_RI_19098 | 0.64 | 0.47-0.86 | 0.00327315 |
| OSBPL3_AD_79025 | 0.64 | 0.47-0.86 | 0.003298772 |
| GALK2_ES_30535 | 0.64 | 0.47-0.86 | 0.00330981 |
| CREB3L2_AT_81912 | 0.64 | 0.47-0.86 | 0.003314751 |
| KRT15_RI_40913 | 0.64 | 0.47-0.86 | 0.003321836 |
| GGA1_AT_62123 | 1.57 | 1.16-2.12 | 0.003321985 |
| AK9_AT_77205 | 0.64 | 0.47-0.86 | 0.003327283 |
| USP31_AP_35576 | 0.64 | 0.47-0.86 | 0.003354429 |
| BCS1L_AD_57544 | 0.64 | 0.47-0.86 | 0.003355773 |
| FBLIM1_AP_771 | 0.64 | 0.47-0.86 | 0.003358569 |
| TUBGCP2_ES_13534 | 0.64 | 0.47-0.86 | 0.003359187 |
| CBWD5_AT_86499 | 1.57 | 1.16-2.12 | 0.003369401 |
| TMEM59_AP_3111 | 1.57 | 1.16-2.12 | 0.003371756 |
| VWA9_AD_31227 | 0.64 | 0.47-0.86 | 0.003374386 |
| TMUB2_ES_41790 | 0.64 | 0.47-0.86 | 0.003375961 |
| SUMO3_AT_60834 | 0.64 | 0.47-0.86 | 0.003379186 |
| P4HA3_AT_17766 | 0.64 | 0.47-0.86 | 0.003388551 |
| OTUD5_AP_89043 | 1.57 | 1.16-2.12 | 0.003391519 |
| ARMCX4_AT_89646 | 1.57 | 1.16-2.12 | 0.003402353 |
| ACOT7_AP_390 | 0.64 | 0.47-0.86 | 0.003406576 |
| AFMID_ES_43802 | 0.64 | 0.47-0.86 | 0.003417011 |
| ZNF365_AT_11877 | 0.64 | 0.47-0.86 | 0.003423791 |
| GSN_AP_87422 | 1.56 | 1.16-2.11 | 0.003428469 |
| ICA1L_AT_56955 | 1.57 | 1.16-2.12 | 0.003430844 |
| LENG8_RI_51898 | 0.64 | 0.47-0.86 | 0.003455359 |
| PTPRU_AD_1428 | 1.56 | 1.16-2.11 | 0.003472794 |
| CD163_AA_20098 | 0.64 | 0.47-0.86 | 0.00348583 |
| CERS5_ES_21681 | 0.64 | 0.47-0.87 | 0.003506545 |
| IL32_AA_33427 | 0.64 | 0.47-0.87 | 0.003550153 |
| SLC25A46_AP_72943 | 0.64 | 0.47-0.87 | 0.003556797 |
| DHX30_AP_64538 | 1.56 | 1.16-2.12 | 0.003564732 |
| DHX30_AP_64539 | 0.64 | 0.47-0.87 | 0.003564732 |
| AKIP1_ES_14279 | 1.56 | 1.16-2.11 | 0.003591182 |
| C19orf66_ES_47453 | 0.64 | 0.47-0.87 | 0.0036368 |
| DENND1A_AT_87515 | 1.56 | 1.15-2.11 | 0.003640886 |
| FAM193B_ES_74800 | 0.64 | 0.47-0.87 | 0.003644082 |
| BTBD8_AT_3754 | 0.64 | 0.47-0.87 | 0.003664183 |
| PPHLN1_AT_21214 | 1.56 | 1.15-2.11 | 0.003702115 |
| CSAD_AP_21944 | 0.64 | 0.47-0.87 | 0.003710629 |
| FOXJ3_RI_2068 | 0.64 | 0.47-0.87 | 0.003728119 |
| OSGIN2_AP_84387 | 0.64 | 0.47-0.87 | 0.003734469 |
| ATP6V1D_AT_28048 | 0.64 | 0.47-0.87 | 0.003756089 |
| ATP6V1D_AT_28049 | 1.56 | 1.15-2.11 | 0.003756089 |
| FHL2_ES_54829 | 0.64 | 0.48-0.87 | 0.00380797 |
| SKA2_ES_42731 | 0.64 | 0.48-0.87 | 0.003830158 |
| SMC6_ES_52732 | 0.64 | 0.47-0.87 | 0.003842807 |
| YLPM1_ES_28429 | 0.64 | 0.47-0.87 | 0.003853086 |
| ASPSCR1_ES_44256 | 0.64 | 0.48-0.87 | 0.003853125 |
| ZNF880_AT_51448 | 1.55 | 1.15-2.10 | 0.003859203 |
| CTPS2_AP_88575 | 0.64 | 0.48-0.87 | 0.003862949 |
| RELL1_AT_69002 | 1.56 | 1.15-2.11 | 0.003863805 |
| RELL1_AT_69003 | 0.64 | 0.47-0.87 | 0.003863805 |
| EZH2_RI_82155 | 0.64 | 0.48-0.87 | 0.003875332 |
| CSAD_AP_21946 | 1.56 | 1.15-2.10 | 0.003899173 |
| UBA52_AP_48466 | 1.56 | 1.15-2.11 | 0.003905147 |
| VEGFA_ES_76328 | 0.64 | 0.47-0.87 | 0.003907352 |
| C19orf54_RI_49966 | 1.56 | 1.15-2.11 | 0.003921982 |
| TLN2_AT_30977 | 1.55 | 1.15-2.10 | 0.003942771 |
| TLN2_AT_30978 | 0.64 | 0.48-0.87 | 0.003942771 |
| SUGP2_RI_48551 | 0.64 | 0.48-0.87 | 0.003943129 |
| PCYOX1L_AP_74032 | 0.64 | 0.48-0.87 | 0.003957032 |
| BAHD1_AA_29983 | 1.55 | 1.15-2.10 | 0.003965137 |
| ABCB8_RI_82298 | 0.64 | 0.47-0.87 | 0.003987125 |
| RNF41_ES_22402 | 0.64 | 0.47-0.87 | 0.004011784 |
| URGCP_AP_79355 | 0.64 | 0.48-0.87 | 0.00404494 |
| WDR6_RI_64794 | 0.64 | 0.48-0.87 | 0.004055583 |
| COG4_ES_37401 | 0.64 | 0.48-0.87 | 0.004063294 |
| CCDC24_ES_2528 | 0.64 | 0.48-0.87 | 0.004080304 |
| THBS3_AA_8026 | 0.64 | 0.48-0.87 | 0.004082121 |
| SRGAP1_AP_22854 | 0.64 | 0.48-0.87 | 0.00408284 |
| C1orf145_AT_10195 | 0.64 | 0.48-0.87 | 0.004086132 |
| ELAC1_AT_45554 | 0.64 | 0.48-0.87 | 0.004094725 |
| ELAC1_AT_45555 | 1.55 | 1.15-2.10 | 0.004094725 |
| FAM192A_AP_36525 | 0.64 | 0.48-0.87 | 0.004101733 |
| TRMU_AA_62709 | 1.55 | 1.15-2.10 | 0.004103596 |
| FAM175A_AT_69798 | 0.64 | 0.48-0.87 | 0.004109179 |
| DGKZ_AA_15547 | 0.64 | 0.48-0.87 | 0.004119526 |
| ING3_RI_81588 | 0.64 | 0.47-0.87 | 0.004148263 |
| ATP8B2_AT_7832 | 1.55 | 1.15-2.10 | 0.004171643 |
| CHORDC1_RI_18266 | 0.64 | 0.48-0.87 | 0.004173075 |
| AKNAD1_AT_3953 | 1.55 | 1.15-2.10 | 0.004176296 |
| FAM102A_AP_87690 | 0.65 | 0.48-0.87 | 0.004180914 |
| FAM102A_AP_87691 | 1.55 | 1.15-2.10 | 0.004180914 |
| FAM73B_AD_87818 | 0.65 | 0.48-0.87 | 0.004256319 |
| FXYD3_RI_49035 | 0.65 | 0.48-0.87 | 0.004260153 |
| GPS1_AA_44281 | 0.65 | 0.48-0.87 | 0.004267572 |
| SDAD1_AD_69574 | 0.65 | 0.48-0.87 | 0.004278507 |
| TTLL12_AP_62573 | 0.65 | 0.48-0.87 | 0.004298546 |
| MAD2L2_ES_665 | 1.55 | 1.15-2.09 | 0.004302471 |
| BANP_ES_37989 | 1.55 | 1.14-2.10 | 0.00430376 |
| GALT_AA_86205 | 0.65 | 0.48-0.87 | 0.004338706 |
| PPP1R26_AP_88108 | 1.55 | 1.14-2.09 | 0.004357458 |
| CPSF3L_ES_140 | 0.65 | 0.48-0.87 | 0.004359041 |
| ZNF333_ES_48022 | 0.65 | 0.48-0.87 | 0.0043886 |
| TUBA1A_AP_21537 | 0.65 | 0.48-0.88 | 0.004413752 |
| C9orf89_AD_86901 | 0.65 | 0.48-0.87 | 0.004427916 |
| MORC2_ES_61809 | 0.65 | 0.48-0.88 | 0.004435692 |
| SRSF11_RI_3380 | 0.65 | 0.48-0.88 | 0.004442286 |
| HNRNPA2B1_RI_79033 | 0.65 | 0.48-0.87 | 0.004450925 |
| NAPEPLD_RI_81172 | 1.55 | 1.14-2.09 | 0.004468374 |
| FBLIM1_AP_769 | 1.55 | 1.14-2.09 | 0.004484316 |
| LDHA_ES_14636 | 0.65 | 0.48-0.88 | 0.004486071 |
| ERCC4_AT_34064 | 1.54 | 1.14-2.09 | 0.004488217 |
| ERCC4_AT_34065 | 0.65 | 0.48-0.88 | 0.004488217 |
| MXRA7_AP_43610 | 0.65 | 0.48-0.88 | 0.004497198 |
| SRSF2_RI_43661 | 0.65 | 0.48-0.88 | 0.004528976 |
| NUCB2_AP_14519 | 0.65 | 0.48-0.88 | 0.004531539 |
| NUCB2_AP_14520 | 1.55 | 1.14-2.09 | 0.004531539 |
| LCLAT1_ES_53117 | 0.65 | 0.48-0.88 | 0.004550468 |
| EPB41L5_AT_55145 | 1.54 | 1.14-2.09 | 0.004577922 |
| EPB41L5_AT_55146 | 0.65 | 0.48-0.88 | 0.004577922 |
| RPL28_AT_52096 | 0.65 | 0.48-0.88 | 0.004590988 |
| BCL2L1_AA_58902 | 0.65 | 0.48-0.88 | 0.004595923 |
| TBL1XR1_AP_67668 | 1.54 | 1.14-2.08 | 0.004651943 |
| YES1_AP_44478 | 0.65 | 0.48-0.88 | 0.004659813 |
| COX17_AT_66351 | 1.54 | 1.14-2.09 | 0.004661428 |
| DPAGT1_RI_19112 | 0.65 | 0.48-0.88 | 0.004672974 |
| CYP2R1_AP_14479 | 0.65 | 0.48-0.88 | 0.004675379 |
| KLHDC9_AA_8553 | 0.65 | 0.48-0.88 | 0.004681155 |
| ARL6IP4_ES_25030 | 0.65 | 0.48-0.88 | 0.004695778 |
| ZNF226_RI_50292 | 0.65 | 0.48-0.88 | 0.004697029 |
| PTTG1IP_AP_60838 | 0.65 | 0.48-0.88 | 0.004755166 |
| PTTG1IP_AP_60839 | 1.54 | 1.14-2.08 | 0.004755166 |
| PKMYT1_AT_33327 | 0.65 | 0.48-0.88 | 0.004767469 |
| PKMYT1_AT_33328 | 1.54 | 1.14-2.09 | 0.004767469 |
| CYFIP1_AP_29674 | 0.65 | 0.48-0.88 | 0.004771402 |
| KANK2_AD_47636 | 1.54 | 1.14-2.09 | 0.004778689 |
| SMIM5_AP_43470 | 0.65 | 0.48-0.88 | 0.004789959 |
| SMIM5_AP_43471 | 1.54 | 1.14-2.09 | 0.004789959 |
| DDX41_AA_74796 | 0.65 | 0.48-0.88 | 0.004808758 |
| LRRC20_ES_12048 | 1.54 | 1.14-2.08 | 0.004813809 |
| NSMF_AT_88309 | 1.54 | 1.14-2.09 | 0.004823648 |
| ACOXL_AT_54942 | 1.54 | 1.14-2.08 | 0.004838835 |
| SGSM3_RI_62346 | 0.65 | 0.48-0.88 | 0.004840094 |
| CARD8_AT_50708 | 0.65 | 0.48-0.88 | 0.004842469 |
| HNRNPUL1_AD_50039 | 0.65 | 0.48-0.88 | 0.004884873 |
| RNF213_AT_44046 | 0.65 | 0.48-0.88 | 0.004899312 |
| DYNC1I1_AT_80542 | 0.65 | 0.48-0.88 | 0.004991267 |
| NSMF_AA_88315 | 0.65 | 0.48-0.88 | 0.005037297 |
| RPE_ME_100824 | 0.65 | 0.48-0.88 | 0.005037798 |
| MGAT4B_AP_74927 | 1.53 | 1.14-2.07 | 0.005079123 |
| MGAT4B_AP_74928 | 0.65 | 0.48-0.88 | 0.005079123 |
| KCTD10_AP_24290 | 0.65 | 0.48-0.88 | 0.005083353 |
| TIA1_ES_53874 | 0.65 | 0.48-0.88 | 0.005155676 |
| INO80E_ES_36015 | 0.65 | 0.48-0.88 | 0.005162404 |
| FRAS1_AT_69640 | 0.65 | 0.48-0.88 | 0.00519255 |
| PRKCZ_AP_238 | 0.65 | 0.48-0.88 | 0.005197723 |
| NBPF10_ES_5531 | 1.53 | 1.13-2.08 | 0.0051998 |
| SERINC5_AT_72644 | 0.65 | 0.48-0.88 | 0.005203685 |
| SERINC5_AT_72645 | 1.53 | 1.13-2.08 | 0.005203685 |
| ACOXL_AT_54943 | 1.53 | 1.13-2.07 | 0.005234896 |
| IL32_RI_33385 | 1.53 | 1.13-2.08 | 0.005273895 |
| METTL17_RI_26475 | 0.65 | 0.48-0.88 | 0.00528914 |
| TNPO1_AP_72474 | 0.65 | 0.48-0.88 | 0.005311609 |
| NKIRAS2_ES_40982 | 0.65 | 0.48-0.88 | 0.005317039 |
| BRICD5_RI_33232 | 0.65 | 0.48-0.88 | 0.005320529 |
| POMT1_AD_87937 | 0.65 | 0.48-0.88 | 0.005322639 |
| DCUN1D4_AD_69271 | 0.65 | 0.48-0.88 | 0.005339906 |
| HSH2D_RI_48138 | 0.65 | 0.48-0.88 | 0.005346853 |
| GGCX_ES_54288 | 1.53 | 1.13-2.07 | 0.005358742 |
| ANKRD46_AD_84712 | 0.65 | 0.48-0.88 | 0.005360415 |
| PARP3_ES_65116 | 0.65 | 0.48-0.88 | 0.005374973 |
| ATP11C_ES_90245 | 0.65 | 0.48-0.88 | 0.005381908 |
| SLC39A13_RI_15741 | 0.65 | 0.48-0.88 | 0.005385925 |
| ICA1L_AT_56954 | 0.65 | 0.48-0.88 | 0.005396977 |
| LUC7L_AP_32836 | 1.53 | 1.13-2.07 | 0.005404211 |
| LUC7L_AP_32837 | 0.65 | 0.48-0.88 | 0.005404211 |
| LHFPL2_AP_72609 | 1.54 | 1.14-2.07 | 0.00543517 |
| EVC_AT_68698 | 1.53 | 1.13-2.06 | 0.00544196 |
| FKBP9_AP_79199 | 1.54 | 1.14-2.08 | 0.005446081 |
| TMEM107_ES_39129 | 0.65 | 0.48-0.88 | 0.005449236 |
| PCSK5_AT_86632 | 0.65 | 0.48-0.88 | 0.005471713 |
| ZNF264_AT_52207 | 1.53 | 1.13-2.07 | 0.005472244 |
| NFIA_AP_3222 | 0.65 | 0.48-0.88 | 0.005477828 |
| RAP1GAP_AA_990 | 0.65 | 0.48-0.88 | 0.005496169 |
| MGRN1_AA_33780 | 0.65 | 0.48-0.88 | 0.005500946 |
| RNF149_AT_54740 | 0.65 | 0.48-0.88 | 0.005502424 |
| RNF149_AT_54741 | 1.53 | 1.13-2.07 | 0.005502424 |
| C14orf80_ES_29661 | 0.65 | 0.48-0.88 | 0.005506093 |
| GIGYF2_ES_58026 | 1.53 | 1.13-2.06 | 0.005506257 |
| DENND1A_AT_87514 | 0.65 | 0.48-0.88 | 0.005508064 |
| NDUFC1_AD_70623 | 1.53 | 1.13-2.08 | 0.00551579 |
| TCF4_AP_45609 | 1.53 | 1.13-2.07 | 0.005535993 |
| CRELD1_ES_63289 | 0.65 | 0.48-0.88 | 0.005538023 |
| RBM4B_AT_17107 | 1.53 | 1.13-2.07 | 0.005543106 |
| RBM4B_AT_17108 | 0.65 | 0.48-0.88 | 0.005543106 |
| IL17RC_AD_63261 | 0.65 | 0.48-0.89 | 0.005551395 |
| TSPAN17_RI_74672 | 0.66 | 0.48-0.89 | 0.00558696 |
| RBM43_AT_55565 | 1.53 | 1.13-2.07 | 0.00559117 |
| RBM43_AT_55566 | 0.65 | 0.48-0.88 | 0.00559117 |
| GIPC1_ES_47983 | 1.53 | 1.13-2.07 | 0.005604877 |
| SMUG1_AP_22121 | 0.65 | 0.48-0.89 | 0.005683511 |
| CRCP_ES_79873 | 0.65 | 0.48-0.89 | 0.005684947 |
| NCAPG2_ES_82527 | 1.53 | 1.13-2.06 | 0.005722815 |
| CBWD6_AT_86489 | 1.53 | 1.13-2.07 | 0.005723676 |
| RUFY2_AA_11939 | 0.66 | 0.49-0.89 | 0.005730608 |
| ZFAND2B_RI_57624 | 0.66 | 0.49-0.89 | 0.00575675 |
| VPS13A_AP_86647 | 0.66 | 0.49-0.89 | 0.005762989 |
| FHL2_ES_54839 | 1.53 | 1.13-2.06 | 0.00577734 |
| PTPLAD2_AT_85991 | 0.65 | 0.48-0.89 | 0.005778016 |
| PTPLAD2_AT_85992 | 1.53 | 1.13-2.07 | 0.005778016 |
| EIF2B1_RI_25100 | 0.66 | 0.49-0.89 | 0.005802138 |
| TSPAN17_RI_74670 | 0.66 | 0.48-0.89 | 0.005819088 |
| URGCP_ES_79367 | 1.52 | 1.13-2.06 | 0.005823422 |
| INO80E_ES_36013 | 0.65 | 0.48-0.89 | 0.00584147 |
| ZNF525_ME_51714 | 0.66 | 0.49-0.89 | 0.005846542 |
| MTFR1L_AP_1198 | 1.52 | 1.13-2.06 | 0.005853862 |
| DPP9_ES_46826 | 0.65 | 0.48-0.89 | 0.005858423 |
| MEF2BNB_ES_48604 | 0.66 | 0.49-0.89 | 0.005864517 |
| HMGN1_AP_60607 | 1.52 | 1.13-2.06 | 0.00589521 |
| ADCK3_AP_10035 | 1.52 | 1.13-2.06 | 0.005924771 |
| ADCK3_AP_10036 | 0.66 | 0.49-0.89 | 0.005924771 |
| GEMIN2_ES_27356 | 0.66 | 0.49-0.89 | 0.005935651 |
| C1QTNF6_AP_62088 | 0.66 | 0.48-0.89 | 0.005939511 |
| ZNF148_ES_66549 | 0.66 | 0.48-0.89 | 0.005949113 |
| TRAP1_AP_33617 | 0.66 | 0.48-0.89 | 0.005949205 |
| TRAP1_AP_33618 | 1.52 | 1.13-2.06 | 0.005949205 |
| PXN_AP_24744 | 1.52 | 1.13-2.06 | 0.005967512 |
| COA6_AP_10335 | 1.52 | 1.13-2.06 | 0.005969286 |
| CTTN_ME_17402 | 1.52 | 1.13-2.06 | 0.005980979 |
| RMDN1_ES_84377 | 0.66 | 0.49-0.89 | 0.005993422 |
| S100A13_AP_7732 | 1.52 | 1.13-2.06 | 0.005995985 |
| DDX11_RI_20963 | 0.66 | 0.49-0.89 | 0.006017843 |
| OS9_RI_22695 | 0.66 | 0.48-0.89 | 0.006020168 |
| ARHGAP32_RI_19451 | 0.66 | 0.49-0.89 | 0.00603846 |
| ZNF774_AA_32485 | 1.53 | 1.12-2.07 | 0.006043198 |
| HKR1_AP_49478 | 0.66 | 0.49-0.89 | 0.006052832 |
| ERGIC3_ES_59178 | 0.66 | 0.49-0.89 | 0.006055173 |
| BTN3A2_AA_75619 | 0.66 | 0.49-0.89 | 0.006058235 |
| SLC12A2_ES_73194 | 0.66 | 0.49-0.89 | 0.006065353 |
| PDE4DIP_AT_4410 | 1.52 | 1.12-2.06 | 0.006069119 |
| ZNF814_AT_52355 | 0.66 | 0.49-0.89 | 0.006081294 |
| DYNLT1_AP_78273 | 0.66 | 0.49-0.89 | 0.006133952 |
| DYNLT1_AP_78274 | 1.52 | 1.13-2.05 | 0.006133952 |
| STAP2_AA_46789 | 0.66 | 0.49-0.89 | 0.006142185 |
| CRELD1_RI_63292 | 0.66 | 0.49-0.89 | 0.00617307 |
| ZC3H7A_AP_34033 | 0.66 | 0.49-0.89 | 0.006184425 |
| MAP4_AP_64547 | 1.52 | 1.12-2.06 | 0.006188445 |
| FHL2_AD_54841 | 1.52 | 1.12-2.06 | 0.006209719 |
| GABPB1_AA_30574 | 1.52 | 1.12-2.05 | 0.006224536 |
| TMEM59_AP_3108 | 0.66 | 0.49-0.89 | 0.006227021 |
| SF1_AA_16681 | 0.66 | 0.49-0.89 | 0.006237874 |
| ATG4B_AD_58398 | 0.66 | 0.49-0.89 | 0.00624063 |
| KHDC1_AP_76736 | 0.66 | 0.49-0.89 | 0.006257384 |
| NPDC1_AP_88260 | 0.66 | 0.49-0.89 | 0.006269423 |
| NPDC1_AP_88261 | 1.52 | 1.12-2.05 | 0.006269423 |
| SECISBP2_AP_86797 | 0.66 | 0.49-0.89 | 0.006306223 |
| WDR62_RI_49339 | 0.66 | 0.49-0.89 | 0.006311938 |
| TMUB1_RI_82347 | 0.66 | 0.49-0.89 | 0.00632196 |
| RDX_ES_18650 | 0.66 | 0.49-0.89 | 0.006324568 |
| TBC1D1_AT_69013 | 1.52 | 1.12-2.05 | 0.006329979 |
| TBC1D1_AT_69014 | 0.66 | 0.49-0.89 | 0.006329979 |
| MTFR1L_ES_1214 | 0.66 | 0.49-0.89 | 0.006330483 |
| FSIP2_AT_56479 | 0.66 | 0.49-0.89 | 0.006341216 |
| FSIP2_AT_56480 | 1.52 | 1.12-2.06 | 0.006341216 |
| ASCC1_AP_12073 | 0.66 | 0.49-0.89 | 0.006349578 |
| PLXDC1_ES_40623 | 0.66 | 0.49-0.89 | 0.006350072 |
| IFRD2_RI_64979 | 0.66 | 0.49-0.89 | 0.006351408 |
| GGT1_AP_61431 | 1.52 | 1.12-2.06 | 0.006378585 |
| TRIM24_AD_81916 | 1.52 | 1.12-2.06 | 0.006393172 |
| GTPBP3_RI_48289 | 0.66 | 0.49-0.89 | 0.006394573 |
| RAD9A_AA_17181 | 1.52 | 1.12-2.05 | 0.006396367 |
| TMEM134_ES_17231 | 0.66 | 0.49-0.89 | 0.006400457 |
| BRD4_AT_48066 | 1.52 | 1.12-2.05 | 0.006402179 |
| OGT_AA_89426 | 1.52 | 1.12-2.05 | 0.006437291 |
| C8orf59_AP_84330 | 1.52 | 1.12-2.05 | 0.006440867 |
| PSEN1_AP_28260 | 1.52 | 1.12-2.05 | 0.00644634 |
| KRT15_ES_40916 | 0.66 | 0.49-0.89 | 0.006446946 |
| PSMC5_RI_43009 | 0.66 | 0.49-0.89 | 0.006463212 |
| DDX19A_AA_37368 | 0.66 | 0.49-0.89 | 0.006470879 |
| IFI44_AT_3554 | 0.66 | 0.49-0.89 | 0.006474148 |
| IFI44_AT_3555 | 1.52 | 1.12-2.05 | 0.006474148 |
| RNF220_AD_2559 | 0.66 | 0.49-0.89 | 0.006499245 |
| ESYT2_AP_82534 | 1.52 | 1.12-2.05 | 0.006507751 |
| ESYT2_AP_82535 | 0.66 | 0.49-0.89 | 0.006507751 |
| CUZD1_AP_13374 | 0.66 | 0.49-0.89 | 0.006512789 |
| WASH4P_RI_32778 | 0.66 | 0.49-0.89 | 0.006532978 |
| AACS_AP_25173 | 1.51 | 1.12-2.04 | 0.00653581 |
| NPIPB4_RI_35506 | 1.52 | 1.12-2.05 | 0.006545887 |
| IMPA2_AT_44659 | 1.52 | 1.12-2.05 | 0.006558707 |
| IMPA2_AT_44660 | 0.66 | 0.49-0.89 | 0.006558707 |
| DEPDC5_ES_61899 | 1.51 | 1.12-2.05 | 0.006570042 |
| ZBTB17_RI_779 | 0.66 | 0.49-0.89 | 0.006625084 |
| ATMIN_AP_37748 | 1.51 | 1.12-2.05 | 0.0066635 |
| EEF1B2_AA_57137 | 0.66 | 0.48-0.89 | 0.006666006 |
| IFT80_AP_67473 | 0.66 | 0.49-0.89 | 0.006711405 |
| HNRNPF_AP_11323 | 1.51 | 1.12-2.05 | 0.00673059 |
| RBMS2_ME_22465 | 1.51 | 1.12-2.04 | 0.006744941 |
| ANKDD1A_AT_31137 | 1.51 | 1.12-2.04 | 0.006766212 |
| POLR2J2_RI_81127 | 0.66 | 0.49-0.89 | 0.006819876 |
| AFMID_ES_94690 | 0.66 | 0.49-0.89 | 0.00684266 |
| PLD2_RI_38595 | 0.66 | 0.49-0.89 | 0.006859087 |
| ATP6V0A2_AP_25113 | 0.66 | 0.49-0.89 | 0.006862269 |
| USP48_AP_994 | 0.66 | 0.49-0.89 | 0.00687067 |
| USP48_AP_995 | 1.51 | 1.12-2.04 | 0.00687067 |
| USE1_RI_48238 | 0.66 | 0.49-0.89 | 0.006882037 |
| LYRM1_AP_34401 | 0.66 | 0.49-0.89 | 0.006891358 |
| HARS_AP_73719 | 1.51 | 1.12-2.04 | 0.006895452 |
| HARS_AP_73720 | 0.66 | 0.49-0.89 | 0.006895452 |
| CEBPG_AP_48937 | 0.66 | 0.49-0.90 | 0.006951042 |
| CEBPG_AP_48938 | 1.51 | 1.12-2.05 | 0.006951042 |
| SIGIRR_RI_13652 | 0.66 | 0.49-0.89 | 0.006963779 |
| SETBP1_AT_45324 | 0.66 | 0.49-0.89 | 0.00696672 |
| SETBP1_AT_45325 | 1.51 | 1.12-2.04 | 0.00696672 |
| HECTD1_AD_27107 | 0.66 | 0.49-0.89 | 0.006967583 |
| DEF8_ES_38194 | 0.66 | 0.49-0.89 | 0.006969104 |
| ABI1_AA_11044 | 0.66 | 0.49-0.90 | 0.00698323 |
| ATXN2L_AD_35844 | 1.51 | 1.12-2.05 | 0.006998941 |
| DENND2D_AP_4142 | 1.51 | 1.12-2.05 | 0.007013765 |
| TTC3_AP_60545 | 0.66 | 0.49-0.90 | 0.007021484 |
| TMUB2_AP_41785 | 1.51 | 1.12-2.05 | 0.007031282 |
| SEMA6C_AD_7562 | 0.66 | 0.49-0.90 | 0.007035634 |
| EFNA3_AP_7937 | 1.51 | 1.12-2.04 | 0.007056695 |
| UBXN4_RI_55449 | 0.66 | 0.49-0.90 | 0.007073818 |
| SIRT1_AP_11907 | 1.51 | 1.12-2.03 | 0.007156156 |
| PAM16_AP_33648 | 1.51 | 1.12-2.04 | 0.007164142 |
| MOK_AT_29371 | 1.51 | 1.12-2.04 | 0.007181683 |
| LIMCH1_AP_69112 | 1.51 | 1.12-2.04 | 0.007185074 |
| IP6K2_RI_64753 | 0.66 | 0.49-0.90 | 0.007186559 |
| CC2D1B_RI_3003 | 0.66 | 0.49-0.90 | 0.007210815 |
| LUC7L_ES_32848 | 0.66 | 0.49-0.90 | 0.007215822 |
| CCAR2_RI_83039 | 0.66 | 0.49-0.90 | 0.007218831 |
| PPP1CC_RI_24503 | 0.66 | 0.49-0.90 | 0.007310314 |
| MMAB_ES_24323 | 0.66 | 0.49-0.90 | 0.007314726 |
| HAT1_AT_55960 | 1.51 | 1.11-2.04 | 0.007316173 |
| ZNF329_ES_52415 | 0.66 | 0.49-0.90 | 0.007340977 |
| NAT9_RI_43298 | 0.66 | 0.49-0.90 | 0.007353749 |
| INO80E_ES_36010 | 0.66 | 0.49-0.90 | 0.007357886 |
| CHORDC1_RI_18267 | 0.66 | 0.49-0.90 | 0.007360188 |
| MPZL2_AT_18983 | 1.51 | 1.11-2.04 | 0.007370641 |
| MPZL2_AT_18984 | 0.66 | 0.49-0.90 | 0.007370641 |
| LETMD1_AD_21745 | 0.66 | 0.49-0.90 | 0.007379817 |
| MKNK1_AA_2808 | 0.66 | 0.49-0.90 | 0.007396665 |
| RAD51AP1_ES_19791 | 1.51 | 1.11-2.04 | 0.007442178 |
| RNF166_RI_38016 | 0.66 | 0.49-0.90 | 0.007447901 |
| ARMCX4_AT_89649 | 0.66 | 0.49-0.90 | 0.007499915 |
| FADS3_RI_16305 | 0.66 | 0.49-0.90 | 0.007508779 |
| PTGER3_AT_3416 | 0.66 | 0.49-0.90 | 0.007515627 |
| C14orf79_AP_29585 | 0.66 | 0.49-0.90 | 0.007531805 |
| C14orf79_AP_29587 | 1.50 | 1.11-2.04 | 0.007531805 |
| SS18_ES_44966 | 0.67 | 0.49-0.90 | 0.007629744 |
| KHDC1_AP_76735 | 1.50 | 1.11-2.03 | 0.00765501 |
| SLC7A6_ES_37213 | 0.66 | 0.49-0.90 | 0.007658589 |
| TDP1_ES_28815 | 0.66 | 0.49-0.90 | 0.007700305 |
| GLOD4_ES_38275 | 0.66 | 0.49-0.90 | 0.00771927 |
| GSN_AP_87421 | 0.67 | 0.49-0.90 | 0.007732283 |
| SSR1_AT_75254 | 1.50 | 1.11-2.03 | 0.007805214 |
| NBPF12_ES_7362 | 1.50 | 1.11-2.03 | 0.007858227 |
| ORAI3_AT_36207 | 0.67 | 0.49-0.90 | 0.007859787 |
| ORAI3_AT_36208 | 1.50 | 1.11-2.03 | 0.007859787 |
| DDX17_AP_62237 | 0.67 | 0.49-0.90 | 0.007896583 |
| DDX17_AP_62238 | 1.50 | 1.11-2.03 | 0.007896583 |
| TMEM107_ES_39130 | 0.67 | 0.49-0.90 | 0.007903929 |
| MTFR1L_AP_1196 | 0.67 | 0.49-0.90 | 0.007932361 |
| C17orf62_AP_44337 | 0.67 | 0.49-0.90 | 0.007946992 |
| BMP2K_AT_69644 | 0.67 | 0.49-0.90 | 0.007960493 |
| BMP2K_AT_69645 | 1.50 | 1.11-2.03 | 0.007960493 |
| RABL5_AT_81059 | 0.67 | 0.49-0.90 | 0.007974141 |
| CYFIP1_AP_29675 | 1.50 | 1.11-2.03 | 0.007979885 |
| SEC31A_ES_69732 | 0.67 | 0.49-0.90 | 0.007997105 |
| ERRFI1_RI_533 | 0.67 | 0.49-0.90 | 0.008017945 |
| YIF1B_AP_49602 | 0.67 | 0.49-0.90 | 0.008028793 |
| THBS3_AA_8029 | 0.67 | 0.49-0.90 | 0.008033946 |
| MAST1_AT_47879 | 0.67 | 0.49-0.90 | 0.008065696 |
| RBBP8_AP_44784 | 1.50 | 1.11-2.03 | 0.008078799 |
| ZNRF1_AT_37576 | 1.50 | 1.11-2.03 | 0.008093805 |
| ZNRF1_AT_37577 | 0.67 | 0.49-0.90 | 0.008093805 |
| BCAT2_ES_50816 | 0.67 | 0.49-0.90 | 0.008116404 |
| STEAP4_AT_80361 | 1.50 | 1.11-2.02 | 0.008137041 |
| STEAP4_AT_80362 | 0.67 | 0.49-0.90 | 0.008137041 |
| SLC25A17_AP_62356 | 1.50 | 1.11-2.02 | 0.008138828 |
| SLC25A17_AP_62357 | 0.67 | 0.49-0.90 | 0.008138828 |
| FLAD1_RI_7867 | 0.67 | 0.49-0.90 | 0.008146342 |
| C1QTNF1_AP_43983 | 0.67 | 0.49-0.90 | 0.00815544 |
| FAM118A_AP_62658 | 0.67 | 0.49-0.90 | 0.008173203 |
| CDK11A_AD_213 | 1.50 | 1.11-2.03 | 0.008180669 |
| NR1H3_ES_15703 | 0.67 | 0.49-0.90 | 0.00818719 |
| C1orf131_RI_10272 | 0.67 | 0.49-0.90 | 0.008192345 |
| APBB3_AD_73680 | 0.67 | 0.49-0.90 | 0.008229003 |
| DMTF1_AA_80302 | 0.67 | 0.49-0.90 | 0.008236117 |
| CASQ1_AP_8433 | 0.67 | 0.49-0.90 | 0.008236892 |
| TSEN34_AP_51808 | 0.67 | 0.49-0.90 | 0.008238023 |
| S100A13_AP_7733 | 0.67 | 0.49-0.90 | 0.00823818 |
| VEZT_ES_23789 | 0.67 | 0.49-0.90 | 0.008256246 |
| TMEM63A_AP_9999 | 1.50 | 1.11-2.02 | 0.008262388 |
| SEC14L1_AP_43701 | 1.50 | 1.11-2.02 | 0.008270588 |
| EFNA3_AP_7936 | 0.67 | 0.49-0.90 | 0.00827097 |
| RAD51B_AT_28110 | 1.50 | 1.11-2.02 | 0.008283071 |
| DYNC2LI1_AA_53399 | 1.50 | 1.11-2.02 | 0.008295894 |
| TLDC2_AT_59327 | 1.50 | 1.11-2.02 | 0.008362683 |
| FBXW7_AP_70849 | 1.50 | 1.11-2.03 | 0.008368917 |
| WHSC1_AA_68528 | 0.67 | 0.49-0.90 | 0.008391297 |
| UBXN11_ES_1261 | 0.67 | 0.49-0.90 | 0.008395887 |
| MMP19_RI_22267 | 0.67 | 0.49-0.90 | 0.008403054 |
| CCDC51_AA_64649 | 1.50 | 1.11-2.02 | 0.00844463 |
| CEP70_AT_66998 | 0.67 | 0.49-0.90 | 0.008489832 |
| NEK10_AT_63771 | 0.67 | 0.49-0.90 | 0.008494926 |
| C9orf156_AP_87023 | 1.50 | 1.11-2.02 | 0.008581629 |
| RABL5_AT_81057 | 1.49 | 1.11-2.02 | 0.008588421 |
| AKAP17A_AT_88392 | 1.49 | 1.11-2.02 | 0.008603683 |
| TMEM88_AA_39057 | 0.67 | 0.49-0.90 | 0.008630521 |
| TBC1D5_AP_63655 | 1.49 | 1.10-2.02 | 0.008635799 |
| DCN_AT_23652 | 1.50 | 1.10-2.02 | 0.00867045 |
| HEXB_AP_72499 | 0.67 | 0.50-0.90 | 0.008670992 |
| TANGO2_AA_61126 | 0.67 | 0.50-0.91 | 0.008716202 |
| ARID5A_AP_54531 | 1.49 | 1.11-2.02 | 0.008736839 |
| GID4_AT_39538 | 0.67 | 0.49-0.91 | 0.008744969 |
| GID4_AT_39539 | 1.49 | 1.10-2.02 | 0.008744969 |
| SHISA5_AP_64686 | 0.67 | 0.50-0.91 | 0.008750805 |
| SYTL2_AP_18142 | 1.49 | 1.10-2.02 | 0.008752926 |
| S100A4_AP_7713 | 1.49 | 1.10-2.02 | 0.008756223 |
| STX17_ES_87069 | 0.67 | 0.50-0.91 | 0.008774222 |
| HP1BP3_AP_937 | 0.67 | 0.49-0.91 | 0.008780177 |
| COA6_AP_10336 | 0.67 | 0.50-0.91 | 0.008789185 |
| CLEC3B_AP_64395 | 0.65 | 0.47-0.91 | 0.008797461 |
| SFSWAP_AD_25216 | 1.49 | 1.10-2.02 | 0.008826968 |
| ANGEL1_AP_28558 | 0.67 | 0.50-0.91 | 0.008853389 |
| ZFAND2B_RI_57625 | 0.67 | 0.50-0.91 | 0.008868434 |
| RALGDS_AP_88016 | 0.67 | 0.50-0.91 | 0.008881726 |
| BBS1_RI_17047 | 0.67 | 0.50-0.91 | 0.008884832 |
| SLC39A14_AT_83007 | 1.49 | 1.10-2.02 | 0.00890789 |
| SLC39A14_AT_83008 | 0.67 | 0.49-0.91 | 0.00890789 |
| ZNF76_AA_75903 | 0.67 | 0.50-0.91 | 0.008933793 |
| VPS35_AD_36307 | 0.67 | 0.50-0.91 | 0.00895617 |
| TBRG1_ES_19224 | 0.67 | 0.50-0.91 | 0.008992919 |
| TMEM205_AD_47659 | 0.67 | 0.50-0.91 | 0.008995248 |
| PTGER3_AT_3415 | 1.50 | 1.10-2.03 | 0.009010773 |
| C8orf59_AP_84329 | 0.67 | 0.50-0.91 | 0.009065487 |
| DCTD_ES_71245 | 0.67 | 0.50-0.91 | 0.009104082 |
| DNAJC2_ES_81189 | 0.67 | 0.50-0.91 | 0.009158465 |
| AKAP17A_AT_88391 | 0.67 | 0.50-0.91 | 0.009174901 |
| COL4A6_AP_89856 | 0.67 | 0.50-0.91 | 0.009183556 |
| TEX264_ES_65105 | 0.67 | 0.50-0.91 | 0.009208905 |
| POMGNT1_RI_2786 | 0.67 | 0.50-0.91 | 0.009230487 |
| FKBP3_ES_27404 | 0.67 | 0.50-0.91 | 0.009241944 |
| NKIRAS2_ES_40978 | 0.67 | 0.50-0.91 | 0.009250233 |
| PJA1_AP_89357 | 0.67 | 0.50-0.91 | 0.009284574 |
| EWSR1_AA_61580 | 1.49 | 1.10-2.01 | 0.009287812 |
| ABI2_ES_57025 | 0.67 | 0.50-0.91 | 0.009291362 |
| FBXO18_ES_10672 | 0.67 | 0.50-0.91 | 0.00930143 |
| REPS1_RI_77954 | 0.67 | 0.50-0.91 | 0.009302732 |
| SEC22C_AP_64287 | 0.67 | 0.50-0.91 | 0.00936433 |
| SLC23A3_AT_57594 | 0.67 | 0.50-0.91 | 0.009392611 |
| CCDC80_AD_66114 | 0.67 | 0.50-0.91 | 0.009466302 |
| C17orf70_AP_44127 | 1.49 | 1.10-2.01 | 0.009509099 |
| C17orf70_AP_44128 | 0.67 | 0.50-0.91 | 0.009509099 |
| RBM6_ES_64951 | 0.67 | 0.50-0.91 | 0.009564981 |
| PCSK7_ES_18901 | 0.67 | 0.50-0.91 | 0.009574305 |
| BCS1L_AD_57552 | 0.67 | 0.50-0.91 | 0.009575294 |
| MAZ_AP_35938 | 0.67 | 0.50-0.91 | 0.00958014 |
| AHCYL2_AP_81742 | 1.49 | 1.10-2.01 | 0.00958015 |
| TXLNA_AP_1558 | 0.67 | 0.50-0.91 | 0.009580514 |
| MCC_AP_73004 | 0.67 | 0.50-0.91 | 0.009593008 |
| PDXP_AP_62142 | 1.49 | 1.10-2.01 | 0.009595969 |
| EPS15L1_AT_48158 | 0.67 | 0.50-0.91 | 0.009603421 |
| KDM5C_ES_89196 | 0.67 | 0.50-0.91 | 0.009623436 |
| SUMF2_ES_79794 | 0.67 | 0.50-0.91 | 0.00963577 |
| C9orf156_AP_87024 | 0.67 | 0.50-0.91 | 0.009652461 |
| NSMF_RI_88313 | 0.67 | 0.50-0.91 | 0.009672101 |
| ERCC5_RI_26230 | 0.67 | 0.50-0.91 | 0.009687777 |
| PMPCB_RI_81177 | 0.67 | 0.50-0.91 | 0.009688542 |
| TMEM63A_AP_10000 | 0.67 | 0.50-0.91 | 0.009699129 |
| RIPK3_AD_26981 | 0.67 | 0.50-0.91 | 0.0097116 |
| SLC23A3_AT_57595 | 1.49 | 1.10-2.01 | 0.009722548 |
| ERRFI1_AA_538 | 0.67 | 0.50-0.91 | 0.009797144 |
| LIG1_AD_50689 | 0.67 | 0.50-0.91 | 0.009812028 |
| TSSC4_ES_13923 | 0.67 | 0.50-0.91 | 0.009829482 |
| ARID5B_AP_11865 | 1.49 | 1.10-2.01 | 0.009850044 |
| PPM1B_AA_53415 | 0.67 | 0.50-0.91 | 0.009877286 |
| CARM1_ES_47600 | 0.67 | 0.50-0.91 | 0.009883671 |
| CREM_AP_11231 | 1.48 | 1.10-2.01 | 0.009890284 |
| TCERG1_ES_73912 | 0.67 | 0.50-0.91 | 0.009905824 |
| CRYZL1_AA_60454 | 0.67 | 0.50-0.91 | 0.009915655 |
| SLC25A46_AP_72944 | 1.48 | 1.10-2.00 | 0.009960828 |
| CCDC24_ES_2531 | 0.67 | 0.50-0.91 | 0.009974135 |
| BSDC1_RI_1591 | 0.67 | 0.50-0.91 | 0.0099825 |
| SREBF2_ES_62465 | 0.67 | 0.50-0.91 | 0.009988164 |
| PML_RI_31631 | 0.67 | 0.50-0.91 | 0.009993614 |
| LSM12_ES_41759 | 0.67 | 0.50-0.91 | 0.010020578 |
| ACAP1_AP_38921 | 0.67 | 0.50-0.91 | 0.010028297 |
| ACP5_ES_47750 | 0.67 | 0.50-0.91 | 0.010044894 |
| SNRPN_AP_29698 | 0.68 | 0.50-0.91 | 0.0100691 |
| GPNMB_AT_78964 | 1.48 | 1.10-2.01 | 0.010074757 |
| GPNMB_AT_78965 | 0.67 | 0.50-0.91 | 0.010074757 |
| ACAA1_AD_64017 | 0.68 | 0.50-0.91 | 0.010078816 |
| CLIP1_AP_24945 | 1.48 | 1.10-2.00 | 0.010114204 |
| CDK10_ES_38125 | 0.67 | 0.50-0.91 | 0.010159258 |
| NDUFS1_AP_57114 | 1.48 | 1.10-2.00 | 0.01017338 |
| KCTD10_AP_24289 | 1.48 | 1.10-2.00 | 0.010173856 |
| FKBP11_AA_21499 | 0.67 | 0.50-0.91 | 0.010203628 |
| ARGLU1_ES_26233 | 0.68 | 0.50-0.91 | 0.010237946 |
| C16orf52_AT_35531 | 1.48 | 1.10-2.00 | 0.01032348 |
| R3HDM2_AP_22566 | 0.68 | 0.50-0.91 | 0.01033746 |
| ATG4B_AD_58397 | 0.68 | 0.50-0.91 | 0.010379432 |
| TMEM150A_AP_54297 | 1.48 | 1.10-2.00 | 0.010429242 |
| PGS1_ES_43881 | 0.67 | 0.50-0.91 | 0.010447329 |
| ZNF530_AT_52302 | 1.48 | 1.09-2.00 | 0.01047061 |
| SUMF2_ES_79798 | 1.48 | 1.09-2.00 | 0.010470628 |
| C1orf159_AD_13 | 0.68 | 0.50-0.91 | 0.010479439 |
| RPLP0_AP_24722 | 1.48 | 1.09-2.00 | 0.010492343 |
| RPLP0_AP_24723 | 0.68 | 0.50-0.91 | 0.010492343 |
| RNH1_ES_13676 | 0.68 | 0.50-0.91 | 0.010518325 |
| MPV17_AP_52961 | 1.48 | 1.09-2.00 | 0.01052244 |
| GPS1_ES_44284 | 0.68 | 0.50-0.91 | 0.010536059 |
| FLNB_ES_65419 | 0.68 | 0.50-0.91 | 0.01056122 |
| TPM1_AP_30981 | 0.68 | 0.50-0.91 | 0.010585725 |
| ME3_AP_18195 | 0.68 | 0.50-0.91 | 0.010606999 |
| IL32_RI_33378 | 0.68 | 0.50-0.91 | 0.010612574 |
| HES2_AT_400 | 1.48 | 1.09-2.00 | 0.010620806 |
| HES2_AT_401 | 0.68 | 0.50-0.92 | 0.010620806 |
| AKAP8L_RI_48081 | 1.48 | 1.09-2.00 | 0.010647928 |
| IST1_ES_37517 | 0.68 | 0.50-0.91 | 0.010676966 |
| RPS6KB2_ES_17204 | 0.68 | 0.50-0.92 | 0.010754521 |
| C14orf105_AT_27658 | 0.62 | 0.42-0.91 | 0.010778694 |
| PPM1N_AP_50473 | 0.68 | 0.50-0.92 | 0.010790667 |
| ANKRD11_AT_38078 | 1.48 | 1.09-2.00 | 0.010822904 |
| FPGT_ES_3452 | 0.68 | 0.50-0.92 | 0.010835744 |
| SMPD4_ES_55292 | 0.68 | 0.50-0.92 | 0.010837373 |
| ZNF365_AT_11879 | 1.48 | 1.09-2.00 | 0.010872662 |
| GNPDA1_AP_73856 | 0.68 | 0.50-0.92 | 0.010872855 |
| PGAP2_ES_14008 | 0.68 | 0.50-0.92 | 0.01088898 |
| METTL23_ES_43632 | 1.48 | 1.09-1.99 | 0.010889644 |
| POLL_RI_12886 | 0.68 | 0.50-0.92 | 0.010891011 |
| SCO2_AP_62848 | 0.68 | 0.50-0.91 | 0.010926664 |
| RFXANK_ES_48608 | 0.68 | 0.50-0.92 | 0.010940034 |
| DCTD_AD_71253 | 0.68 | 0.50-0.92 | 0.010954763 |
| ARFGAP1_ES_60111 | 0.68 | 0.50-0.92 | 0.010956219 |
| DMKN_RI_49146 | 0.68 | 0.50-0.92 | 0.010987139 |
| NGFRAP1_AP_89730 | 1.47 | 1.09-1.99 | 0.011004564 |
| KIF22_AD_35935 | 0.68 | 0.50-0.92 | 0.011043016 |
| APOD_ES_68181 | 0.68 | 0.50-0.92 | 0.0110931 |
| WRAP53_AP_39043 | 1.48 | 1.09-2.00 | 0.011105249 |
| CDKN2A_AT_86005 | 1.48 | 1.09-2.00 | 0.011120755 |
| REXO2_AP_18830 | 1.47 | 1.09-1.99 | 0.011149994 |
| REXO2_AP_18831 | 0.68 | 0.50-0.92 | 0.011149994 |
| ZCCHC8_AP_24960 | 0.68 | 0.50-0.92 | 0.011163961 |
| SLC3A2_ES_16467 | 1.47 | 1.09-1.99 | 0.011165618 |
| PJA1_AP_89358 | 1.47 | 1.09-1.99 | 0.011184403 |
| GCLC_AP_76498 | 0.68 | 0.50-0.92 | 0.011225115 |
| FAM65A_AP_37081 | 0.68 | 0.50-0.92 | 0.011234256 |
| TRIM29_AP_19152 | 1.48 | 1.09-2.00 | 0.011236619 |
| TNFRSF18_AP_34 | 1.47 | 1.09-1.99 | 0.011238126 |
| MLXIP_AP_24922 | 0.68 | 0.50-0.92 | 0.011272145 |
| MLXIP_AP_24923 | 1.47 | 1.09-1.99 | 0.011272145 |
| TJAP1_AA_76276 | 0.68 | 0.50-0.92 | 0.01128996 |
| RNF121_ES_17462 | 0.68 | 0.50-0.92 | 0.011297113 |
| TPM1_AP_30980 | 0.68 | 0.50-0.92 | 0.011299461 |
| THNSL2_ME_54469 | 1.47 | 1.09-1.99 | 0.011350322 |
| ARFIP1_ES_70858 | 0.68 | 0.50-0.92 | 0.011356134 |
| MAST4_AP_72276 | 0.68 | 0.50-0.92 | 0.011390291 |
| PALLD_AP_71123 | 1.47 | 1.09-1.99 | 0.011419238 |
| TBC1D5_AP_63658 | 0.68 | 0.50-0.92 | 0.011419825 |
| FARP1_AP_26163 | 0.68 | 0.50-0.92 | 0.011422309 |
| METTL23_ES_43633 | 1.47 | 1.09-1.99 | 0.011462574 |
| PIGO_RI_86233 | 1.47 | 1.09-1.99 | 0.01148488 |
| AMPD2_AP_4047 | 1.47 | 1.09-1.99 | 0.011500188 |
| COMMD4_ES_31851 | 0.68 | 0.50-0.92 | 0.011515575 |
| CMTM4_AT_36827 | 1.47 | 1.09-1.99 | 0.011520601 |
| CMTM4_AT_36828 | 0.68 | 0.50-0.92 | 0.011520601 |
| NAT9_ES_43294 | 0.68 | 0.50-0.92 | 0.011540602 |
| MAST1_AT_47878 | 1.47 | 1.09-2.00 | 0.011624094 |
| USP10_ES_37864 | 0.68 | 0.50-0.92 | 0.011632781 |
| AIM1_AP_77120 | 1.47 | 1.09-1.99 | 0.011638456 |
| SAR1A_AP_12032 | 0.68 | 0.50-0.92 | 0.01167761 |
| ZNF37A_ES_11312 | 0.68 | 0.50-0.92 | 0.011695912 |
| ETV7_ES_75976 | 1.47 | 1.09-1.99 | 0.011696012 |
| INVS_AT_87074 | 0.68 | 0.50-0.92 | 0.011730794 |
| DUSP14_AP_40525 | 0.68 | 0.50-0.92 | 0.011730985 |
| ZNF626_AT_48723 | 1.47 | 1.09-1.99 | 0.011747381 |
| ZNF626_AT_48724 | 0.68 | 0.50-0.92 | 0.011747381 |
| UPF1_AD_48507 | 0.68 | 0.50-0.92 | 0.011757711 |
| SUV420H1_ES_17298 | 0.68 | 0.50-0.92 | 0.011768523 |
| MSTO1_RI_8091 | 0.68 | 0.50-0.92 | 0.01182271 |
| SMN1_RI_72421 | 0.68 | 0.50-0.92 | 0.011897498 |
| INTS6_AT_25938 | 1.47 | 1.09-1.99 | 0.011950241 |
| INTS6_AT_25940 | 0.68 | 0.50-0.92 | 0.011950241 |
| MORN1_AT_252 | 1.47 | 1.09-1.99 | 0.011979031 |
| RPL14_AD_64220 | 0.68 | 0.50-0.92 | 0.011979905 |
| MLH1_AD_63953 | 0.68 | 0.50-0.92 | 0.01201362 |
| TAF1A_AT_9884 | 1.47 | 1.09-1.99 | 0.012026352 |
| OS9_RI_22696 | 0.68 | 0.50-0.92 | 0.012029355 |
| CLCF1_AP_17175 | 1.47 | 1.09-1.99 | 0.012087801 |
| CAPN2_AP_9923 | 1.47 | 1.09-1.98 | 0.012121767 |
| CAPN2_AP_9924 | 0.68 | 0.50-0.92 | 0.012121767 |
| PIK3C3_AP_45313 | 1.47 | 1.09-1.98 | 0.012145816 |
| MRS2_AA_75508 | 0.68 | 0.51-0.92 | 0.012161596 |
| OSGIN2_AP_84388 | 1.47 | 1.09-1.98 | 0.012172461 |
| TNFRSF12A_ES_33347 | 0.68 | 0.50-0.92 | 0.012176514 |
| DIS3L2_AT_57983 | 0.68 | 0.50-0.92 | 0.012201621 |
| C7orf10_AT_79305 | 1.47 | 1.08-1.99 | 0.012214267 |
| C7orf10_AT_79306 | 0.68 | 0.50-0.92 | 0.012214267 |
| CGGBP1_AA_65668 | 0.68 | 0.50-0.92 | 0.012252373 |
| CARKD_AA_26254 | 0.68 | 0.51-0.92 | 0.012279692 |
| CHMP2A_AP_52483 | 1.47 | 1.08-1.99 | 0.012288302 |
| ARFRP1_AP_60151 | 0.68 | 0.51-0.92 | 0.012354723 |
| USP40_AP_58045 | 0.68 | 0.51-0.92 | 0.01236759 |
| TM2D1_AT_3232 | 1.47 | 1.08-1.98 | 0.012377389 |
| YAF2_ES_21155 | 1.46 | 1.08-1.98 | 0.012444994 |
| FAM195A_ES_32927 | 0.68 | 0.51-0.92 | 0.01244582 |
| TMUB2_AT_41787 | 0.68 | 0.51-0.92 | 0.012448944 |
| CARKD_RI_26255 | 0.68 | 0.51-0.92 | 0.012480498 |
| CGREF1_AT_52934 | 1.47 | 1.08-1.98 | 0.012481186 |
| PGM3_AP_76859 | 1.47 | 1.08-1.98 | 0.012495292 |
| GABPB2_ES_7559 | 1.47 | 1.08-1.98 | 0.012529131 |
| SZT2_AT_2177 | 0.68 | 0.50-0.92 | 0.012564868 |
| SP140L_RI_57890 | 0.68 | 0.51-0.92 | 0.012599683 |
| LIN37_RI_49283 | 0.68 | 0.51-0.92 | 0.012602364 |
| TM2D1_AT_3230 | 0.68 | 0.51-0.92 | 0.012604542 |
| PILRB_ES_80935 | 0.68 | 0.50-0.92 | 0.012647833 |
| TRMT10B_AD_86433 | 0.68 | 0.50-0.92 | 0.012651229 |
| ERMAP_AP_2122 | 0.68 | 0.50-0.92 | 0.012701616 |
| CTBP1_AP_68460 | 0.68 | 0.50-0.92 | 0.012705441 |
| CTBP1_AP_68461 | 1.47 | 1.08-1.98 | 0.012705441 |
| RRP7A_ES_62507 | 0.68 | 0.51-0.92 | 0.012744904 |
| CCDC24_ES_2527 | 0.68 | 0.51-0.92 | 0.012839541 |
| GPATCH8_AT_41869 | 0.68 | 0.51-0.92 | 0.012891443 |
| ARL16_ES_44150 | 0.68 | 0.51-0.92 | 0.012898645 |
| NDUFB5_ES_67706 | 1.46 | 1.08-1.98 | 0.012907345 |
| ACTG1_AD_44122 | 1.46 | 1.08-1.97 | 0.012915211 |
| NADK_ES_229 | 0.68 | 0.51-0.92 | 0.012930912 |
| SEC11A_ES_32313 | 1.46 | 1.08-1.98 | 0.012940255 |
| SLC16A3_AP_44300 | 1.46 | 1.08-1.97 | 0.012963977 |
| ATG4D_ES_47538 | 0.68 | 0.50-0.92 | 0.012972185 |
| ADAM15_ES_7910 | 0.68 | 0.51-0.92 | 0.012982917 |
| MYH11_ES_34188 | 1.46 | 1.08-1.98 | 0.012999845 |
| ALS2CL_RI_64463 | 0.68 | 0.51-0.92 | 0.013023496 |
| ZSWIM7_RI_39391 | 0.68 | 0.51-0.92 | 0.013061795 |
| INTS10_AD_82887 | 0.68 | 0.51-0.92 | 0.013066731 |
| GJB2_AP_25420 | 0.68 | 0.51-0.92 | 0.013105065 |
| SREBF1_AP_39500 | 1.46 | 1.08-1.97 | 0.013134587 |
| TM4SF19_AT_68239 | 0.68 | 0.51-0.93 | 0.013156838 |
| DCK_AD_69460 | 0.68 | 0.51-0.93 | 0.013164364 |
| SRSF11_AA_3391 | 0.68 | 0.50-0.93 | 0.013164477 |
| MOBP_AT_64190 | 1.46 | 1.08-1.97 | 0.013174056 |
| AMZ2_ES_43129 | 1.46 | 1.08-1.97 | 0.013185855 |
| PELP1_ES_38555 | 0.68 | 0.51-0.92 | 0.013196923 |
| ARHGEF16_AP_309 | 1.46 | 1.08-1.97 | 0.013208247 |
| C9orf72_AT_86052 | 0.69 | 0.51-0.93 | 0.013234636 |
| C9orf72_AT_86053 | 1.46 | 1.08-1.97 | 0.013234636 |
| SUCLA2_AP_25849 | 0.68 | 0.51-0.93 | 0.013321779 |
| MPND_ES_46795 | 0.69 | 0.51-0.93 | 0.013336432 |
| GSTK1_RI_82082 | 0.69 | 0.51-0.93 | 0.013395077 |
| GPT2_ES_36318 | 0.68 | 0.51-0.93 | 0.013407295 |
| WHSC1_RI_68534 | 0.69 | 0.51-0.93 | 0.013525226 |
| SPP1_ES_69873 | 0.69 | 0.51-0.93 | 0.013580753 |
| BCS1L_AD_57548 | 0.69 | 0.51-0.93 | 0.013645821 |
| GPS1_AA_44280 | 1.46 | 1.08-1.97 | 0.013655392 |
| C20orf196_AT_58656 | 1.46 | 1.08-1.97 | 0.013671289 |
| C20orf196_AT_58657 | 0.69 | 0.51-0.93 | 0.013671289 |
| MCCC1_ES_67777 | 0.69 | 0.51-0.93 | 0.013699892 |
| SERPINB1_ES_75145 | 0.69 | 0.51-0.93 | 0.013727223 |
| AP5S1_AA_58605 | 0.69 | 0.51-0.93 | 0.013735949 |
| FCF1_AA_28423 | 0.69 | 0.51-0.93 | 0.013736789 |
| CD55_ES_9637 | 0.69 | 0.51-0.93 | 0.01373714 |
| UBAP2L_AD_7816 | 1.46 | 1.08-1.97 | 0.013764641 |
| ATG4D_ES_47535 | 0.69 | 0.51-0.93 | 0.013785614 |
| MCF2L_AT_26326 | 0.69 | 0.51-0.93 | 0.013795724 |
| MAP4_AP_64546 | 0.60 | 0.39-0.92 | 0.013799895 |
| LRTOMT_ES_17539 | 0.69 | 0.51-0.93 | 0.013812938 |
| EPB41L1_ES_59273 | 1.46 | 1.08-1.97 | 0.013876185 |
| TRAPPC2P1_RI_52233 | 0.69 | 0.51-0.93 | 0.013907973 |
| KIFC3_AP_36606 | 0.69 | 0.51-0.93 | 0.01390803 |
| ZSCAN9_ES_75722 | 0.69 | 0.51-0.93 | 0.01392663 |
| CNTNAP3B_AT_86482 | 0.69 | 0.51-0.93 | 0.013949975 |
| TADA2B_AP_68733 | 0.69 | 0.51-0.93 | 0.013971907 |
| OFD1_ES_88522 | 1.46 | 1.08-1.97 | 0.013982362 |
| TBC1D23_ES_65817 | 1.46 | 1.08-1.97 | 0.014013385 |
| WDR33_AT_55244 | 1.46 | 1.08-1.97 | 0.014016 |
| EIF3C_AP_35825 | 0.69 | 0.51-0.93 | 0.014062081 |
| SEPT5_AP_61071 | 1.46 | 1.08-1.97 | 0.014067512 |
| TMEM120B_RI_24893 | 1.45 | 1.08-1.97 | 0.014072853 |
| P4HA1_ES_12123 | 0.69 | 0.51-0.93 | 0.01410281 |
| ZNF66_AP_48725 | 1.46 | 1.08-1.97 | 0.014143617 |
| ITCH_ES_59022 | 0.69 | 0.51-0.93 | 0.014148123 |
| B3GAT3_AA_16371 | 1.45 | 1.08-1.97 | 0.014195068 |
| ZFC3H1_RI_23406 | 0.69 | 0.51-0.93 | 0.014205888 |
| TTC14_RI_67728 | 0.69 | 0.51-0.93 | 0.014212676 |
| SNX13_RI_78881 | 0.69 | 0.51-0.93 | 0.014221281 |
| KLC4_AD_76230 | 0.69 | 0.51-0.93 | 0.014270605 |
| CXorf40A_AT_90300 | 0.69 | 0.51-0.93 | 0.014278854 |
| CXorf40A_AT_90301 | 1.46 | 1.08-1.97 | 0.014278854 |
| SLAIN2_ES_69214 | 0.69 | 0.51-0.93 | 0.014335579 |
| XYLB_AT_64043 | 0.69 | 0.51-0.93 | 0.014337188 |
| XYLB_AT_64044 | 1.46 | 1.08-1.97 | 0.014337188 |
| MAPKAPK2_AA_9592 | 0.69 | 0.51-0.93 | 0.014395985 |
| TMEM107_ES_39127 | 0.69 | 0.51-0.93 | 0.014470424 |
| COPS7A_AD_19956 | 0.69 | 0.51-0.93 | 0.014479371 |
| TPD52_AT_84270 | 0.69 | 0.51-0.93 | 0.014508835 |
| TPD52_AT_84271 | 1.46 | 1.08-1.97 | 0.014508835 |
| USP31_AP_35575 | 1.45 | 1.07-1.97 | 0.014513473 |
| APOL4_ES_62005 | 0.69 | 0.51-0.93 | 0.014542751 |
| MCC_AP_73005 | 1.45 | 1.08-1.97 | 0.014573273 |
| ZNF74_ES_61152 | 0.69 | 0.51-0.93 | 0.014583294 |
| SLC30A5_AA_72303 | 0.69 | 0.51-0.93 | 0.014620286 |
| LMO7_ES_26067 | 1.45 | 1.07-1.97 | 0.01462948 |
| SLC50A1_AP_7940 | 0.69 | 0.51-0.93 | 0.014666124 |
| AIM1_AP_77119 | 0.69 | 0.51-0.93 | 0.014669841 |
| ARID5A_AP_54530 | 0.69 | 0.51-0.93 | 0.014675145 |
| HAT1_AT_55961 | 0.69 | 0.51-0.93 | 0.01468431 |
| C11orf74_AP_15435 | 1.45 | 1.07-1.96 | 0.014696341 |
| DONSON_ES_60445 | 1.45 | 1.07-1.96 | 0.014716995 |
| RAD21_AP_84980 | 1.45 | 1.07-1.96 | 0.01472725 |
| TMEM234_RI_1571 | 1.45 | 1.07-1.96 | 0.014729662 |
| ANKLE2_AP_25295 | 1.45 | 1.07-1.97 | 0.014734536 |
| ANKLE2_AP_25296 | 0.69 | 0.51-0.93 | 0.014734536 |
| PDCD2_RI_78501 | 0.69 | 0.51-0.93 | 0.014742445 |
| SLC44A3_AP_3818 | 0.69 | 0.51-0.93 | 0.014773365 |
| WDPCP_AP_53725 | 1.45 | 1.07-1.96 | 0.014774161 |
| PIAS3_RI_7292 | 0.69 | 0.51-0.93 | 0.014782004 |
| ARL5A_AD_55597 | 1.45 | 1.07-1.96 | 0.014815322 |
| RAN_AD_25205 | 0.69 | 0.51-0.93 | 0.014824829 |
| PLK1_AD_35629 | 0.69 | 0.51-0.93 | 0.014831163 |
| UBA1_AP_88908 | 0.69 | 0.51-0.93 | 0.014838566 |
| PGS1_AA_43873 | 1.45 | 1.07-1.96 | 0.014861668 |
| C21orf59_AD_60359 | 0.69 | 0.51-0.93 | 0.014861774 |
| RBM14-RBM4_ES_17102 | 1.45 | 1.07-1.95 | 0.014878918 |
| DGKA_ME_22303 | 1.45 | 1.07-1.96 | 0.014937805 |
| PI4KB_ES_7591 | 0.69 | 0.51-0.93 | 0.014954183 |
| PCBP4_ES_65137 | 0.69 | 0.51-0.93 | 0.014958899 |
| PHACTR2_AP_77982 | 0.69 | 0.51-0.93 | 0.014980142 |
| MYO19_AA_40482 | 0.69 | 0.51-0.93 | 0.015038821 |
| D2HGDH_ES_58423 | 0.69 | 0.51-0.93 | 0.015069056 |
| CSTF3_AT_14883 | 1.45 | 1.07-1.96 | 0.015087428 |
| CLSPN_AT_1730 | 1.45 | 1.07-1.96 | 0.015098056 |
| CLSPN_AT_1731 | 0.69 | 0.51-0.93 | 0.015098056 |
| PIK3C3_AP_45315 | 0.69 | 0.51-0.93 | 0.015116358 |
| FAM86A_ES_33883 | 1.45 | 1.07-1.96 | 0.015129191 |
| FN3KRP_ES_44418 | 0.69 | 0.51-0.93 | 0.015149333 |
| RPL28_RI_52097 | 0.69 | 0.51-0.93 | 0.015167914 |
| FBN1_AT_30497 | 0.69 | 0.51-0.93 | 0.015182502 |
| FBN1_AT_30499 | 1.45 | 1.07-1.96 | 0.015182502 |
| SLC25A14_RI_90098 | 0.69 | 0.51-0.93 | 0.015215409 |
| BTBD8_AT_3753 | 1.45 | 1.07-1.96 | 0.015230726 |
| PRMT1_AP_51039 | 0.69 | 0.51-0.93 | 0.015258281 |
| ZNF780B_AD_49855 | 0.69 | 0.51-0.93 | 0.015261787 |
| C1orf109_AA_1816 | 0.69 | 0.51-0.93 | 0.015263429 |
| TMEM180_ES_12954 | 0.69 | 0.51-0.93 | 0.015284048 |
| MRRF_ES_87470 | 0.69 | 0.51-0.93 | 0.015291904 |
| LIG1_AA_50690 | 0.69 | 0.51-0.93 | 0.015313338 |
| RARA_AP_40857 | 1.45 | 1.07-1.96 | 0.015336722 |
| NPIPB5_AP_35558 | 0.69 | 0.51-0.93 | 0.01536083 |
| UBAP2_AP_86127 | 0.69 | 0.51-0.93 | 0.015364317 |
| MFSD11_RI_43686 | 0.69 | 0.51-0.93 | 0.015414682 |
| ARFRP1_AP_60152 | 1.45 | 1.07-1.95 | 0.015468246 |
| ASCC2_ES_61687 | 0.69 | 0.51-0.93 | 0.015468438 |
| C7orf49_AD_81878 | 0.69 | 0.51-0.93 | 0.015473885 |
| TGIF1_AP_44497 | 1.45 | 1.07-1.96 | 0.015478867 |
| KIAA1191_ES_74644 | 0.69 | 0.51-0.93 | 0.015482909 |
| FBLN5_ES_28893 | 0.69 | 0.51-0.93 | 0.015486859 |
| ZNF185_ES_90404 | 0.69 | 0.51-0.93 | 0.015494833 |
| DCAF8_ES_8441 | 0.69 | 0.51-0.93 | 0.01550083 |
| NCOA6_AP_59034 | 1.45 | 1.07-1.97 | 0.01551223 |
| NCOA6_AP_59035 | 0.69 | 0.51-0.93 | 0.01551223 |
| AFMID_ES_43810 | 0.69 | 0.51-0.93 | 0.015563471 |
| MYD88_AD_64029 | 0.69 | 0.51-0.93 | 0.015580979 |
| INO80E_ES_36004 | 0.69 | 0.51-0.93 | 0.015590027 |
| RPAP1_ES_30096 | 0.69 | 0.51-0.93 | 0.01559093 |
| TMEM63B_AP_76352 | 0.69 | 0.51-0.93 | 0.015596388 |
| CPSF3L_AA_80 | 0.69 | 0.51-0.93 | 0.015598982 |
| FGD3_RI_86895 | 0.69 | 0.51-0.93 | 0.015649675 |
| ABCC1_ES_34214 | 1.45 | 1.07-1.95 | 0.015653528 |
| EIF5_AP_29442 | 1.45 | 1.07-1.95 | 0.01566497 |
| STAMBPL1_AP_12469 | 0.69 | 0.51-0.93 | 0.015678431 |
| DLX4_AT_42338 | 0.69 | 0.51-0.93 | 0.015687341 |
| LRP8_ES_3058 | 1.45 | 1.07-1.95 | 0.015700795 |
| PVR_AD_50340 | 0.69 | 0.51-0.93 | 0.015706273 |
| SLC15A4_RI_25187 | 0.69 | 0.51-0.93 | 0.015755071 |
| ZNF250_AT_85674 | 0.69 | 0.51-0.93 | 0.015762973 |
| TMUB2_AT_41786 | 1.45 | 1.07-1.95 | 0.015852729 |
| CNKSR1_RI_1236 | 0.69 | 0.51-0.94 | 0.015853649 |
| SNW1_RI_28620 | 0.69 | 0.51-0.93 | 0.015864867 |
| EIF3C_AP_35827 | 1.45 | 1.07-1.95 | 0.015878399 |
| BCS1L_ES_57546 | 0.69 | 0.51-0.93 | 0.015892674 |
| NDUFS1_AP_57113 | 0.69 | 0.51-0.94 | 0.015906763 |
| CDK17_AT_23827 | 1.45 | 1.07-1.96 | 0.015912356 |
| CDK17_AT_23828 | 0.69 | 0.51-0.94 | 0.015912356 |
| ADAT2_AP_77977 | 1.45 | 1.07-1.96 | 0.015912383 |
| ADAT2_AP_77978 | 0.69 | 0.51-0.94 | 0.015912383 |
| SMAD4_AP_45557 | 1.45 | 1.07-1.95 | 0.015912997 |
| PIDD_AA_13769 | 0.69 | 0.51-0.94 | 0.015936101 |
| USE1_AT_48235 | 0.69 | 0.51-0.94 | 0.015969826 |
| SCRN2_AA_42122 | 0.69 | 0.51-0.93 | 0.015970611 |
| PRMT1_AP_51038 | 1.45 | 1.07-1.95 | 0.015970928 |
| C9orf89_RI_86898 | 0.69 | 0.51-0.93 | 0.015995353 |
| PLS3_AP_89921 | 0.69 | 0.51-0.93 | 0.015997324 |
| WDR75_AA_56504 | 0.69 | 0.51-0.94 | 0.016016895 |
| STAMBP_AT_53987 | 1.45 | 1.07-1.95 | 0.016056368 |
| STAMBP_AT_53988 | 0.69 | 0.51-0.94 | 0.016056368 |
| FAM192A_AP_36526 | 0.69 | 0.51-0.94 | 0.016132052 |
| PLA2G15_ES_37203 | 0.69 | 0.51-0.94 | 0.016135194 |
| TMEM161B_AD_72733 | 0.69 | 0.51-0.94 | 0.016142343 |
| PMS1_AT_56543 | 1.45 | 1.07-1.95 | 0.016153633 |
| PMS1_AT_56544 | 0.69 | 0.51-0.94 | 0.016153633 |
| ANKRD46_ES_84711 | 0.69 | 0.51-0.94 | 0.016161068 |
| FAM131A_AP_67924 | 0.69 | 0.51-0.94 | 0.016161945 |
| IL11RA_AP_86208 | 1.44 | 1.07-1.95 | 0.016169878 |
| PARVB_AP_62606 | 1.45 | 1.07-1.95 | 0.016207394 |
| FAM126A_ES_78946 | 0.69 | 0.51-0.94 | 0.016230988 |
| NMRK1_ES_86630 | 0.69 | 0.51-0.94 | 0.016240615 |
| GALNS_AT_38032 | 1.44 | 1.07-1.95 | 0.016243981 |
| DCTN1_RI_54046 | 0.69 | 0.51-0.94 | 0.016262019 |
| TEX30_AP_26215 | 0.69 | 0.51-0.94 | 0.016296896 |
| KALRN_AT_66522 | 0.69 | 0.51-0.94 | 0.016309603 |
| ZNF83_ES_51506 | 0.69 | 0.51-0.94 | 0.016359833 |
| POLR2F_AT_62180 | 1.44 | 1.07-1.95 | 0.016416433 |
| CUZD1_AP_13376 | 1.44 | 1.07-1.95 | 0.016421245 |
| SMARCD3_RI_82363 | 0.69 | 0.51-0.94 | 0.016436886 |
| SLC9B2_AP_70169 | 1.44 | 1.07-1.95 | 0.016441924 |
| NFIA_AP_3224 | 1.44 | 1.07-1.95 | 0.016445501 |
| SLC44A1_AP_87111 | 1.44 | 1.07-1.95 | 0.01645465 |
| IPO4_AA_26885 | 0.69 | 0.51-0.94 | 0.016464092 |
| STARD3_ES_40657 | 0.69 | 0.51-0.94 | 0.016467664 |
| SLC3A2_AP_16463 | 0.69 | 0.51-0.94 | 0.016553666 |
| BICC1_AT_11818 | 1.44 | 1.07-1.95 | 0.016583716 |
| RPL34_AT_70300 | 1.44 | 1.07-1.95 | 0.016591705 |
| USE1_AT_48234 | 1.44 | 1.07-1.95 | 0.016607833 |
| PEX10_AD_268 | 1.44 | 1.07-1.95 | 0.016650426 |
| LILRB5_AA_51850 | 1.44 | 1.07-1.95 | 0.016695805 |
| HSPA14_AT_10830 | 0.69 | 0.51-0.94 | 0.016702026 |
| HSPA14_AT_10831 | 1.44 | 1.07-1.95 | 0.016702026 |
| ARHGEF3_AP_65360 | 0.69 | 0.51-0.94 | 0.016707761 |
| NDUFA3_AT_51775 | 0.69 | 0.51-0.94 | 0.016732266 |
| POMGNT1_RI_2787 | 0.69 | 0.51-0.94 | 0.016747339 |
| FAM111A_AA_16027 | 0.69 | 0.51-0.94 | 0.016761484 |
| BTAF1_ES_12524 | 0.69 | 0.51-0.94 | 0.016765064 |
| PRPF39_ES_27396 | 0.69 | 0.51-0.94 | 0.016833091 |
| PCYT2_AA_44231 | 0.69 | 0.51-0.94 | 0.01683767 |
| ANGEL1_AP_28557 | 1.44 | 1.07-1.95 | 0.016855732 |
| TRAPPC6A_ES_50410 | 0.69 | 0.51-0.94 | 0.016860597 |
| METAP2_AA_23795 | 1.44 | 1.07-1.94 | 0.01687355 |
| PTGR1_ES_87222 | 0.69 | 0.51-0.94 | 0.016887898 |
| FBXO44_AD_656 | 1.44 | 1.07-1.94 | 0.016943418 |
| SERPINE3_AT_25931 | 0.69 | 0.51-0.94 | 0.017006463 |
| NASP_ES_2754 | 0.69 | 0.51-0.94 | 0.017010147 |
| DIDO1_AP_60090 | 1.44 | 1.07-1.95 | 0.01702204 |
| NEIL3_AT_71226 | 0.69 | 0.51-0.94 | 0.017024329 |
| NEIL3_AT_71227 | 1.44 | 1.07-1.95 | 0.017024329 |
| PLA2G6_ES_62205 | 0.69 | 0.51-0.94 | 0.017072826 |
| ATF4_RI_62313 | 0.69 | 0.51-0.94 | 0.017089349 |
| RBM5_RI_64960 | 0.69 | 0.51-0.94 | 0.017151942 |
| THOP1_AP_46624 | 1.44 | 1.07-1.94 | 0.017183509 |
| SLC37A3_AA_81987 | 0.69 | 0.51-0.94 | 0.017196989 |
| AGL_AP_3853 | 1.44 | 1.06-1.95 | 0.017229 |
| B3GALNT2_AT_10352 | 1.44 | 1.07-1.94 | 0.01723096 |
| FUBP1_AA_3544 | 0.69 | 0.51-0.94 | 0.017237179 |
| CUL4B_AP_90003 | 0.69 | 0.51-0.94 | 0.017262773 |
| PTPN6_AP_20022 | 1.44 | 1.06-1.94 | 0.017277816 |
| PTPN6_AP_20023 | 0.70 | 0.51-0.94 | 0.017277816 |
| NME6_ES_64587 | 0.70 | 0.52-0.94 | 0.017286653 |
| CCDC159_ES_47685 | 0.70 | 0.51-0.94 | 0.017307451 |
| TM4SF19_AT_68240 | 1.44 | 1.06-1.94 | 0.017321355 |
| SAMD4B_ES_49806 | 0.70 | 0.52-0.94 | 0.017430267 |
| TLDC2_AT_59328 | 0.69 | 0.51-0.94 | 0.01746212 |
| UCP3_AT_17757 | 0.70 | 0.51-0.94 | 0.017495001 |
| UCP3_AT_17758 | 1.44 | 1.06-1.94 | 0.017495001 |
| D2HGDH_ES_58421 | 1.44 | 1.06-1.94 | 0.017522752 |
| GNPDA1_AA_73864 | 0.70 | 0.52-0.94 | 0.01752563 |
| KIFC3_AP_36605 | 1.44 | 1.06-1.94 | 0.017539668 |
| DNM3_AT_9000 | 0.70 | 0.51-0.94 | 0.017556225 |
| ADSSL1_AP_29549 | 0.70 | 0.52-0.94 | 0.017615568 |
| TAOK3_AP_24699 | 0.70 | 0.51-0.94 | 0.017618106 |
| FECH_AA_45634 | 0.70 | 0.51-0.94 | 0.01761849 |
| CEP290_AP_23606 | 1.44 | 1.06-1.94 | 0.017620707 |
| DIDO1_AT_60093 | 0.70 | 0.52-0.94 | 0.017632758 |
| RARG_AP_21979 | 0.70 | 0.52-0.94 | 0.01763607 |
| RARG_AP_21980 | 1.44 | 1.06-1.94 | 0.01763607 |
| ASAH1_AP_82843 | 0.70 | 0.51-0.94 | 0.017649253 |
| ASAH1_AP_82844 | 1.44 | 1.06-1.94 | 0.017649253 |
| FN3KRP_ES_44419 | 0.70 | 0.52-0.94 | 0.01765174 |
| HNRNPA1_RI_22144 | 0.70 | 0.52-0.94 | 0.017662528 |
| SEPT9_AP_43723 | 1.44 | 1.06-1.94 | 0.017664244 |
| CDK10_RI_38115 | 0.70 | 0.52-0.94 | 0.017694868 |
| METTL23_ES_43634 | 1.44 | 1.06-1.94 | 0.01772803 |
| CTNS_AT_38471 | 1.44 | 1.06-1.95 | 0.017777732 |
| CTNS_AT_38472 | 0.70 | 0.51-0.94 | 0.017777732 |
| FAM131A_AP_67927 | 1.44 | 1.06-1.94 | 0.017814138 |
| ABCC4_AT_26108 | 1.44 | 1.06-1.94 | 0.017851217 |
| ABCC4_AT_26110 | 0.70 | 0.52-0.94 | 0.017851217 |
| ACTR6_ES_23910 | 0.70 | 0.51-0.94 | 0.017889106 |
| SON_ES_60438 | 0.70 | 0.51-0.94 | 0.017914964 |
| SEPT5_AP_61072 | 0.70 | 0.52-0.94 | 0.017915249 |
| ATP2A2_RI_24418 | 0.69 | 0.51-0.94 | 0.017925234 |
| LDLR_AA_47618 | 0.70 | 0.51-0.94 | 0.017957803 |
| DZIP3_RI_66037 | 0.70 | 0.51-0.94 | 0.017962881 |
| DCTN2_ES_22641 | 0.70 | 0.51-0.94 | 0.017989077 |
| LMBR1L_ES_21525 | 0.70 | 0.52-0.94 | 0.017998654 |
| EXOC7_ES_43570 | 0.70 | 0.52-0.94 | 0.018002587 |
| INO80E_AA_36018 | 0.70 | 0.52-0.94 | 0.018013962 |
| ZNF585B_AP_49454 | 0.70 | 0.52-0.94 | 0.018014533 |
| MOK_AP_29361 | 0.70 | 0.52-0.94 | 0.018031995 |
| ENO2_AP_20012 | 1.44 | 1.06-1.94 | 0.018050156 |
| RPL35_AD_87538 | 0.70 | 0.52-0.94 | 0.018067127 |
| B9D1_RI_39715 | 0.70 | 0.52-0.94 | 0.018068879 |
| GRHPR_AP_86399 | 0.70 | 0.52-0.94 | 0.018071752 |
| GRHPR_AP_86400 | 1.43 | 1.06-1.93 | 0.018071752 |
| ENTPD6_ES_58864 | 0.70 | 0.51-0.94 | 0.018090348 |
| FASTK_RI_82334 | 0.70 | 0.52-0.94 | 0.018098994 |
| GGT1_AP_61430 | 1.43 | 1.06-1.94 | 0.018112844 |
| TSNARE1_ES_85346 | 0.70 | 0.52-0.94 | 0.018122579 |
| HYOU1_ES_19084 | 0.70 | 0.52-0.94 | 0.018161908 |
| FAM107B_AP_10817 | 1.43 | 1.06-1.93 | 0.018175778 |
| SDHA_ES_71420 | 1.43 | 1.06-1.93 | 0.018235133 |
| GTF2H1_AP_14598 | 0.70 | 0.52-0.94 | 0.018243437 |
| ZNF532_AP_45682 | 0.70 | 0.52-0.94 | 0.018279827 |
| MAX_RI_27939 | 0.70 | 0.52-0.94 | 0.018281135 |
| TMX2_ES_15919 | 0.70 | 0.52-0.94 | 0.018297415 |
| SLC35A5_AP_66109 | 1.43 | 1.06-1.94 | 0.018302831 |
| CRTAC1_AT_12749 | 0.69 | 0.51-0.94 | 0.018360539 |
| MLLT10_ES_10974 | 0.70 | 0.52-0.94 | 0.01836562 |
| ARFGAP2_ES_15652 | 1.43 | 1.06-1.94 | 0.018433803 |
| SLC50A1_AP_7941 | 1.43 | 1.06-1.94 | 0.018440771 |
| CTAGE5_AP_27374 | 0.70 | 0.51-0.94 | 0.018447835 |
| SH3BP1_ES_62141 | 0.70 | 0.52-0.94 | 0.018459616 |
| WSB2_AP_24687 | 0.70 | 0.51-0.94 | 0.018521025 |
| METTL6_AT_63573 | 1.43 | 1.06-1.94 | 0.018526602 |
| CGREF1_AT_52933 | 0.70 | 0.52-0.94 | 0.018533209 |
| C11orf74_AP_15436 | 0.70 | 0.51-0.94 | 0.018551789 |
| MUC1_ES_7959 | 0.70 | 0.52-0.94 | 0.018572602 |
| ZNF585B_AP_49455 | 1.43 | 1.06-1.94 | 0.018604044 |
| DENND2D_AP_4141 | 0.70 | 0.52-0.94 | 0.018657124 |
| ZNF519_AT_44756 | 1.43 | 1.06-1.94 | 0.018680295 |
| ANAPC5_RI_24863 | 0.70 | 0.52-0.94 | 0.018735197 |
| LRRC36_AT_37015 | 0.70 | 0.52-0.94 | 0.018742264 |
| FAM213A_AP_12364 | 0.70 | 0.52-0.94 | 0.018748677 |
| OXNAD1_ES_63642 | 0.70 | 0.52-0.94 | 0.018782054 |
| ATP5C1_ES_10726 | 0.70 | 0.52-0.94 | 0.018802513 |
| E2F8_AP_14690 | 0.70 | 0.52-0.94 | 0.018838511 |
| C1orf85_ES_8233 | 0.70 | 0.51-0.94 | 0.018851067 |
| ZNF85_AT_48734 | 0.70 | 0.52-0.94 | 0.018869835 |
| ZNF85_AT_48735 | 1.43 | 1.06-1.94 | 0.018869835 |
| TFAP2A_AP_75290 | 1.43 | 1.06-1.94 | 0.018892076 |
| UBALD1_RI_33767 | 0.70 | 0.52-0.94 | 0.018939237 |
| NPIPB4_ES_35509 | 0.70 | 0.52-0.94 | 0.018945667 |
| MARK3_ES_29448 | 1.43 | 1.06-1.94 | 0.018950884 |
| ARHGEF18_AP_47100 | 1.43 | 1.06-1.93 | 0.018954007 |
| ABCC5_AA_67818 | 0.70 | 0.51-0.94 | 0.018982418 |
| PRKACB_AP_3584 | 1.43 | 1.06-1.94 | 0.019009319 |
| PIGG_AD_68372 | 0.70 | 0.52-0.94 | 0.019146412 |
| NAT6_AA_64989 | 0.70 | 0.52-0.94 | 0.019218602 |
| NIN_ES_27495 | 1.43 | 1.06-1.93 | 0.019282503 |
| KANSL2_RI_21460 | 0.70 | 0.52-0.94 | 0.019287295 |
| STRADA_RI_42962 | 0.70 | 0.52-0.95 | 0.019362498 |
| DIAPH2_AT_89594 | 1.43 | 1.06-1.93 | 0.019382814 |
| DIAPH2_AT_89595 | 0.70 | 0.52-0.94 | 0.019382814 |
| AKNAD1_AT_3954 | 1.43 | 1.06-1.94 | 0.019384193 |
| FADS1_AP_16299 | 1.43 | 1.06-1.94 | 0.019434105 |
| EXOC7_ES_43565 | 0.70 | 0.52-0.95 | 0.019454064 |
| RIMKLB_AP_20199 | 1.43 | 1.06-1.93 | 0.019477814 |
| NSMF_AT_88308 | 0.70 | 0.52-0.95 | 0.01949283 |
| KNOP1_AP_34328 | 0.70 | 0.52-0.95 | 0.019502623 |
| METRNL_AP_44432 | 1.43 | 1.06-1.93 | 0.019515208 |
| WASH4P_AA_32782 | 1.43 | 1.06-1.93 | 0.019567297 |
| CA5B_ES_98313 | 1.43 | 1.06-1.93 | 0.019587074 |
| DLX4_AT_42337 | 1.43 | 1.06-1.93 | 0.019623698 |
| TMCO6_RI_73694 | 0.70 | 0.52-0.95 | 0.019633841 |
| NSMCE2_ES_85127 | 1.43 | 1.06-1.93 | 0.019641109 |
| NARF_AT_44395 | 1.43 | 1.06-1.93 | 0.019647148 |
| NARF_AT_44396 | 0.70 | 0.52-0.94 | 0.019647148 |
| INO80E_ES_36012 | 0.70 | 0.52-0.95 | 0.019647269 |
| TMEM39B_AT_1543 | 0.70 | 0.52-0.95 | 0.019655682 |
| TMEM39B_AT_1545 | 1.43 | 1.06-1.93 | 0.019655682 |
| GTF2IRD2_AT_80092 | 1.43 | 1.06-1.93 | 0.01968806 |
| TBC1D20_RI_58458 | 0.70 | 0.52-0.95 | 0.019729951 |
| KLC4_AA_76228 | 0.70 | 0.52-0.95 | 0.01973395 |
| CORO7_AD_33669 | 0.70 | 0.52-0.95 | 0.01973728 |
| ELP3_AP_83202 | 0.70 | 0.52-0.95 | 0.019759476 |
| STRA6_AD_31688 | 0.70 | 0.52-0.95 | 0.019772706 |
| SMYD5_AA_53956 | 0.70 | 0.52-0.95 | 0.019781811 |
| PQLC1_ES_46267 | 1.43 | 1.06-1.92 | 0.019842252 |
| ZNF841_AP_51403 | 1.43 | 1.06-1.93 | 0.019853702 |
| TP53_ES_39039 | 0.70 | 0.52-0.95 | 0.019909013 |
| MRPL55_ES_10105 | 0.70 | 0.52-0.95 | 0.019923085 |
| SKA2_ES_42734 | 0.70 | 0.52-0.95 | 0.0199384 |
| NXF1_RI_16435 | 0.70 | 0.52-0.95 | 0.019997923 |
| SIPA1_AP_16887 | 0.70 | 0.52-0.95 | 0.019999766 |
| TCF12_AP_30784 | 0.70 | 0.52-0.95 | 0.02003035 |
| GPATCH8_AT_41868 | 1.43 | 1.06-1.93 | 0.020070048 |
| STRA13_ES_44265 | 0.70 | 0.52-0.95 | 0.020082514 |
| MSL3_AP_88484 | 1.43 | 1.06-1.93 | 0.02009682 |
| ARTN_AP_2483 | 0.70 | 0.52-0.95 | 0.020113986 |
| NDRG1_ES_85238 | 0.70 | 0.52-0.95 | 0.020155473 |
| PPP1CA_ES_17183 | 0.70 | 0.52-0.95 | 0.020165325 |
| CAMKK2_AP_24847 | 0.70 | 0.52-0.95 | 0.020183919 |
| PLEKHG6_AP_19820 | 0.70 | 0.52-0.95 | 0.020189244 |
| UBE2D4_AD_79373 | 0.70 | 0.52-0.95 | 0.020194276 |
| DAGLB_AD_78725 | 0.70 | 0.52-0.95 | 0.020216793 |
| CERS5_ES_21659 | 0.70 | 0.52-0.95 | 0.020232832 |
| S100PBP_AT_1635 | 1.43 | 1.05-1.93 | 0.020235031 |
| S100PBP_AT_1636 | 0.70 | 0.52-0.95 | 0.020235031 |
| HSF4_ES_36943 | 0.70 | 0.52-0.95 | 0.020245858 |
| PDHA1_AT_88631 | 0.70 | 0.52-0.95 | 0.020320174 |
| PFKM_ES_21429 | 0.70 | 0.52-0.95 | 0.020324726 |
| ZNF365_AT_11878 | 1.42 | 1.06-1.92 | 0.020354079 |
| STK3_ES_84657 | 0.70 | 0.52-0.95 | 0.020400569 |
| HOOK2_AP_47863 | 1.42 | 1.06-1.92 | 0.020401139 |
| C20orf24_ES_59296 | 1.43 | 1.05-1.93 | 0.020416509 |
| ARSA_AD_62901 | 1.42 | 1.05-1.93 | 0.020418163 |
| SUCLA2_AP_25850 | 1.43 | 1.05-1.93 | 0.020429431 |
| PPM1M_RI_65192 | 0.70 | 0.52-0.95 | 0.020451878 |
| ABCB9_AT_24998 | 1.42 | 1.05-1.92 | 0.020495427 |
| LRRC37A_AP_42021 | 0.70 | 0.52-0.95 | 0.020552316 |
| LRRC37A_AP_42022 | 1.42 | 1.05-1.92 | 0.020552316 |
| CHMP7_ES_83073 | 0.70 | 0.52-0.95 | 0.02056145 |
| AURKAIP1_RI_148 | 1.43 | 1.05-1.93 | 0.020576518 |
| SLC25A12_ES_55957 | 0.70 | 0.52-0.95 | 0.020637987 |
| BRD4_AT_48069 | 0.70 | 0.52-0.95 | 0.020641974 |
| TNPO1_AP_72475 | 1.42 | 1.05-1.92 | 0.020644035 |
| SH2D3C_AP_87664 | 1.42 | 1.05-1.92 | 0.020662178 |
| SRSF5_AA_28158 | 0.70 | 0.52-0.95 | 0.020673233 |
| VGLL4_AP_63390 | 0.70 | 0.52-0.95 | 0.02068128 |
| SAR1A_AP_12034 | 1.42 | 1.05-1.92 | 0.02071377 |
| CNTNAP3B_AT_86484 | 1.42 | 1.05-1.92 | 0.02075392 |
| SNX5_AT_58744 | 0.70 | 0.52-0.95 | 0.020765527 |
| SNX5_AT_58745 | 1.42 | 1.05-1.93 | 0.020765527 |
| MADD_AD_15720 | 1.42 | 1.05-1.93 | 0.020785214 |
| PCGF3_RI_68405 | 0.70 | 0.52-0.95 | 0.020788848 |
| MPDU1_AT_38997 | 0.70 | 0.52-0.95 | 0.020810058 |
| UBTF_AP_41831 | 0.70 | 0.52-0.95 | 0.020817558 |
| SEC22C_AT_64289 | 1.42 | 1.05-1.92 | 0.020822482 |
| SEC22C_AT_64290 | 0.70 | 0.52-0.95 | 0.020822482 |
| AARSD1_ES_94557 | 1.42 | 1.05-1.92 | 0.020831773 |
| PSTPIP1_AT_31962 | 1.42 | 1.05-1.92 | 0.020856659 |
| C8orf44_AT_84029 | 0.70 | 0.52-0.95 | 0.020890331 |
| LEPRE1_RI_2106 | 0.70 | 0.52-0.95 | 0.020902311 |
| FAM13A_AT_69905 | 1.42 | 1.05-1.93 | 0.02093098 |
| HDAC8_AT_89459 | 0.70 | 0.52-0.95 | 0.02093464 |
| GALK2_ES_30524 | 0.70 | 0.52-0.95 | 0.020964124 |
| DGAT1_RI_85562 | 0.70 | 0.52-0.95 | 0.020989657 |
| C8orf44_AT_84028 | 1.42 | 1.05-1.93 | 0.021015696 |
| GPS1_AD_44283 | 0.70 | 0.52-0.95 | 0.021021806 |
| RBMS2_ES_22470 | 0.70 | 0.52-0.95 | 0.021062283 |
| PHYKPL_ES_74860 | 0.70 | 0.52-0.95 | 0.021070411 |
| FBLN5_AP_28888 | 1.42 | 1.06-1.92 | 0.021113691 |
| PSMC3IP_AD_41080 | 0.70 | 0.52-0.95 | 0.021131625 |
| DEF8_AT_38180 | 1.42 | 1.05-1.92 | 0.021147537 |
| DEF8_AT_38182 | 0.70 | 0.52-0.95 | 0.021147537 |
| SQSTM1_AP_74934 | 0.70 | 0.52-0.95 | 0.021171805 |
| PPA1_ES_12045 | 0.70 | 0.52-0.95 | 0.02123524 |
| CIDEB_AP_26957 | 0.70 | 0.52-0.95 | 0.021285504 |
| CIDEB_AP_26958 | 1.42 | 1.05-1.92 | 0.021285504 |
| SNED1_AT_58306 | 0.70 | 0.52-0.95 | 0.021290788 |
| TTLL5_AT_28518 | 1.42 | 1.05-1.92 | 0.021320618 |
| ANXA8L1_ES_11445 | 1.42 | 1.05-1.92 | 0.021322995 |
| FHL2_AD_54842 | 1.42 | 1.05-1.92 | 0.021360647 |
| TM4SF19_AT_68238 | 1.42 | 1.05-1.92 | 0.021388972 |
| EFCAB13_AT_42070 | 0.70 | 0.52-0.95 | 0.021412018 |
| SREBF1_AP_39501 | 0.70 | 0.52-0.95 | 0.021412863 |
| RPL37A_RI_57422 | 0.70 | 0.52-0.95 | 0.021415134 |
| TTC8_ES_28787 | 0.70 | 0.52-0.95 | 0.021435282 |
| ADSSL1_AP_29550 | 1.42 | 1.05-1.92 | 0.021438699 |
| XAF1_AA_38812 | 0.70 | 0.52-0.95 | 0.021449189 |
| LUC7L3_RI_42474 | 0.70 | 0.52-0.95 | 0.021479034 |
| AKAP17A_RI_88395 | 0.70 | 0.52-0.95 | 0.021564037 |
| CECR5_AP_60966 | 0.70 | 0.52-0.95 | 0.021577802 |
| LEPRE1_RI_2107 | 0.70 | 0.52-0.95 | 0.021583376 |
| DAPL1_AT_55687 | 1.42 | 1.05-1.92 | 0.021599074 |
| SRSF11_AP_3373 | 0.70 | 0.52-0.95 | 0.021606166 |
| B3GALNT2_AT_10353 | 0.70 | 0.52-0.95 | 0.021632808 |
| SNRNP40_ES_1459 | 0.70 | 0.52-0.95 | 0.02163596 |
| DNM3_AT_9002 | 1.42 | 1.05-1.92 | 0.021685641 |
| AGPAT6_ES_83526 | 0.70 | 0.52-0.95 | 0.021698742 |
| TMEM185A_AT_90317 | 0.70 | 0.52-0.95 | 0.021731425 |
| ELF3_RI_9395 | 0.70 | 0.52-0.95 | 0.02176528 |
| BLOC1S6_ES_30456 | 1.42 | 1.05-1.92 | 0.02180693 |
| KLC2_AP_16988 | 1.42 | 1.05-1.92 | 0.02181155 |
| POM121_AT_79927 | 1.42 | 1.05-1.92 | 0.021873289 |
| POM121_AT_79928 | 0.70 | 0.52-0.95 | 0.021873289 |
| NEK2_AT_9717 | 1.42 | 1.05-1.92 | 0.021874952 |
| NEK2_AT_9718 | 0.70 | 0.52-0.95 | 0.021874952 |
| BFAR_ES_34095 | 0.70 | 0.52-0.95 | 0.021882431 |
| UGGT2_AT_26129 | 0.70 | 0.52-0.95 | 0.021885878 |
| FXYD3_AP_49027 | 0.70 | 0.52-0.95 | 0.021968489 |
| PSEN1_AP_28263 | 0.70 | 0.52-0.95 | 0.021977581 |
| RPAP1_RI_30095 | 0.70 | 0.52-0.95 | 0.022027675 |
| HIRA_AP_61048 | 0.70 | 0.52-0.95 | 0.022047879 |
| RUFY1_ES_74895 | 0.70 | 0.52-0.95 | 0.02205783 |
| PCSK7_RI_18902 | 0.70 | 0.52-0.95 | 0.022076614 |
| AUH_AT_86823 | 0.70 | 0.52-0.95 | 0.022098088 |
| ZNF410_ES_28327 | 0.70 | 0.52-0.95 | 0.022135434 |
| MRPL55_RI_10087 | 0.71 | 0.52-0.95 | 0.02222925 |
| ATMIN_AP_37747 | 0.70 | 0.52-0.95 | 0.022236648 |
| IL32_RI_33381 | 1.42 | 1.05-1.92 | 0.02227371 |
| RPAIN_ES_38690 | 1.42 | 1.05-1.91 | 0.022277679 |
| MCUR1_ES_75402 | 0.71 | 0.52-0.95 | 0.022283487 |
| MCFD2_ES_53478 | 0.71 | 0.52-0.95 | 0.022395396 |
| INO80C_ES_45184 | 1.42 | 1.05-1.91 | 0.022423424 |
| APPL2_AP_24127 | 0.71 | 0.52-0.95 | 0.022445941 |
| STAP2_AA_46791 | 1.42 | 1.05-1.91 | 0.022446191 |
| TMEM53_ES_2568 | 1.42 | 1.05-1.92 | 0.022462514 |
| TMEM55B_RI_26454 | 0.71 | 0.52-0.95 | 0.022463686 |
| VPS28_RI_85604 | 0.71 | 0.52-0.95 | 0.022501568 |
| RBM10_ES_88906 | 0.71 | 0.52-0.95 | 0.022513886 |
| TUBB3_AP_38166 | 0.70 | 0.52-0.95 | 0.022522788 |
| UBA2_AP_48963 | 0.71 | 0.52-0.95 | 0.022528336 |
| SCNN1A_ES_19842 | 0.71 | 0.52-0.95 | 0.022595093 |
| SNCA_AT_69929 | 1.42 | 1.05-1.91 | 0.022622511 |
| SERPINE3_AT_25932 | 1.42 | 1.05-1.91 | 0.022624818 |
| CXorf40A_RI_90306 | 0.71 | 0.52-0.95 | 0.022626191 |
| WDR62_RI_49337 | 0.71 | 0.52-0.95 | 0.022639345 |
| CAMK2D_AD_70406 | 1.42 | 1.05-1.92 | 0.02264937 |
| HKR1_ES_49490 | 1.42 | 1.05-1.91 | 0.022649435 |
| VSIG2_AT_19231 | 1.42 | 1.05-1.91 | 0.022675599 |
| VSIG2_AT_19232 | 0.71 | 0.52-0.95 | 0.022675599 |
| SEMA3C_ES_80243 | 0.71 | 0.52-0.95 | 0.022678286 |
| SLCO3A1_AT_32546 | 1.42 | 1.05-1.91 | 0.022686223 |
| SLCO3A1_AT_32547 | 0.71 | 0.52-0.95 | 0.022686223 |
| TCEA3_AP_1059 | 0.71 | 0.52-0.95 | 0.022687773 |
| HEATR5A_AT_27111 | 0.71 | 0.52-0.95 | 0.022689557 |
| PRSS27_AT_33295 | 1.42 | 1.05-1.91 | 0.022697343 |
| PRSS27_AT_33296 | 0.71 | 0.52-0.95 | 0.022697343 |
| CCDC142_AA_54075 | 0.71 | 0.52-0.95 | 0.02270084 |
| MAP7D1_ES_1761 | 0.71 | 0.52-0.95 | 0.02270275 |
| PLEKHG6_AP_19821 | 1.42 | 1.05-1.92 | 0.022739347 |
| ZNF692_RI_10563 | 0.71 | 0.52-0.95 | 0.022744759 |
| ODF2L_ES_3674 | 0.71 | 0.52-0.95 | 0.022765182 |
| PIK3C3_AP_45312 | 0.71 | 0.52-0.95 | 0.022844644 |
| ORMDL1_RI_56537 | 0.71 | 0.52-0.95 | 0.022924404 |
| SYMPK_AP_50529 | 1.42 | 1.05-1.91 | 0.022929038 |
| DCAKD_AP_41928 | 0.71 | 0.52-0.95 | 0.022950612 |
| TSPAN9_ES_19753 | 0.71 | 0.52-0.95 | 0.022962332 |
| USP36_AT_43912 | 0.71 | 0.52-0.95 | 0.023030063 |
| C12orf65_AP_25059 | 0.71 | 0.52-0.95 | 0.023037311 |
| EPHA3_AT_65671 | 0.71 | 0.52-0.96 | 0.023089425 |
| EPHA3_AT_65672 | 1.42 | 1.05-1.92 | 0.023089425 |
| TNFRSF18_AP_33 | 0.71 | 0.52-0.95 | 0.023104716 |
| SIDT2_RI_18886 | 0.71 | 0.52-0.95 | 0.023143503 |
| AGTRAP_ES_671 | 0.71 | 0.52-0.95 | 0.023171551 |
| PSMG3_AP_78589 | 0.71 | 0.52-0.96 | 0.023178027 |
| IL4I1_AP_51119 | 1.42 | 1.05-1.91 | 0.023228383 |
| EBPL_ES_25912 | 1.42 | 1.05-1.92 | 0.023234612 |
| PSMC3IP_AD_41076 | 0.71 | 0.52-0.96 | 0.023240403 |
| PFKFB2_AT_9616 | 0.71 | 0.52-0.96 | 0.02324535 |
| PFKFB2_AT_9617 | 1.41 | 1.05-1.91 | 0.02324535 |
| SREBF1_RI_39506 | 0.71 | 0.52-0.95 | 0.023294482 |
| ZNF720_ES_36291 | 0.71 | 0.52-0.96 | 0.023336014 |
| GINS4_RI_83519 | 0.71 | 0.52-0.96 | 0.023351153 |
| KCNMB3_AP_67679 | 1.41 | 1.05-1.91 | 0.023393533 |
| ATXN2L_RI_35833 | 0.71 | 0.52-0.96 | 0.023407103 |
| IFNAR1_AP_60398 | 0.71 | 0.52-0.96 | 0.023467571 |
| IFNAR1_AP_60399 | 1.41 | 1.05-1.91 | 0.023467571 |
| HOMER3_AP_48533 | 0.71 | 0.52-0.96 | 0.023469142 |
| EPB41L2_AP_77527 | 0.71 | 0.52-0.96 | 0.023479004 |
| NUSAP1_ES_30084 | 0.71 | 0.52-0.96 | 0.023562565 |
| PNPLA8_ES_81415 | 0.71 | 0.52-0.96 | 0.023645087 |
| ULK1_AP_25223 | 0.71 | 0.52-0.96 | 0.023656341 |
| COA1_ES_79347 | 1.41 | 1.05-1.91 | 0.023660818 |
| COLCA1_AP_18662 | 1.41 | 1.05-1.91 | 0.02366967 |
| PJA1_AA_89360 | 1.41 | 1.05-1.91 | 0.023676187 |
| RNPS1_AD_33255 | 0.71 | 0.52-0.96 | 0.023693626 |
| ABCB9_AP_24994 | 0.71 | 0.52-0.96 | 0.023693773 |
| ZNF799_AP_47798 | 0.71 | 0.52-0.96 | 0.023693775 |
| TUBGCP6_AD_62794 | 0.71 | 0.52-0.96 | 0.023718042 |
| LIMS3L_AT_54938 | 0.71 | 0.52-0.96 | 0.023773981 |
| BMP1_ES_82990 | 0.71 | 0.52-0.96 | 0.023782779 |
| G3BP2_AP_69548 | 1.41 | 1.05-1.91 | 0.0237876 |
| SYTL2_ES_18148 | 0.71 | 0.52-0.96 | 0.023794539 |
| PQBP1_AD_89028 | 0.71 | 0.52-0.96 | 0.023795527 |
| TMEM33_RI_69133 | 1.41 | 1.05-1.91 | 0.023829033 |
| SENP7_AP_65945 | 0.71 | 0.52-0.96 | 0.023830062 |
| SPATA20_RI_42424 | 0.71 | 0.52-0.96 | 0.02383576 |
| NBPF11_ES_7346 | 1.41 | 1.05-1.91 | 0.023895947 |
| CLEC1A_ES_20304 | 0.71 | 0.52-0.96 | 0.023930362 |
| ANKRD11_AT_38079 | 0.71 | 0.52-0.96 | 0.02394799 |
| PHF12_RI_40024 | 0.71 | 0.52-0.96 | 0.024001063 |
| SYNRG_AA_40528 | 1.41 | 1.05-1.90 | 0.024031157 |
| ADAM15_ES_7911 | 0.71 | 0.52-0.96 | 0.024053282 |
| ZNF398_AP_82171 | 1.41 | 1.05-1.91 | 0.024060332 |
| UBE2V2_AP_83796 | 0.71 | 0.52-0.96 | 0.024176592 |
| UBE2V2_AP_83797 | 1.41 | 1.04-1.91 | 0.024176592 |
| EML3_AA_16365 | 0.71 | 0.52-0.96 | 0.024178001 |
| NBPF15_ES_7397 | 0.71 | 0.52-0.96 | 0.024184781 |
| CSTF3_AT_14884 | 0.71 | 0.53-0.96 | 0.024249799 |
| TMEM176A_AD_82265 | 0.71 | 0.52-0.96 | 0.024250107 |
| ZNF655_AT_80662 | 1.41 | 1.04-1.91 | 0.024264314 |
| ZNF655_AT_80663 | 0.71 | 0.52-0.96 | 0.024264314 |
| TAPT1_ES_68830 | 1.41 | 1.04-1.91 | 0.024271378 |
| SYT8_RI_13859 | 0.71 | 0.53-0.96 | 0.02429736 |
| TMEM91_AP_50043 | 1.41 | 1.04-1.91 | 0.02435277 |
| NFYC_AA_2019 | 0.71 | 0.53-0.96 | 0.024359044 |
| SLC25A29_ES_29259 | 0.71 | 0.52-0.96 | 0.024458229 |
| TOMM40_RI_50350 | 0.71 | 0.53-0.96 | 0.024473298 |
| HLX_RI_9879 | 0.71 | 0.53-0.96 | 0.024529872 |
| TCAIM_RI_64360 | 0.71 | 0.53-0.96 | 0.024539731 |
| USP36_RI_43916 | 0.71 | 0.53-0.96 | 0.024540211 |
| ZNF276_AP_38136 | 1.41 | 1.04-1.91 | 0.024556075 |
| SRSF1_RI_42630 | 1.41 | 1.04-1.90 | 0.024562417 |
| CDV3_AA_66837 | 0.71 | 0.52-0.96 | 0.024588657 |
| RRAS2_AP_14457 | 1.41 | 1.04-1.90 | 0.02459318 |
| TEX30_RI_26217 | 0.71 | 0.53-0.96 | 0.024596942 |
| TBC1D2_AP_87036 | 1.41 | 1.04-1.90 | 0.024597983 |
| TBC1D2_AP_87037 | 0.71 | 0.53-0.96 | 0.024597983 |
| MCF2L_AT_26328 | 1.41 | 1.04-1.90 | 0.024617119 |
| ZSCAN9_AT_75720 | 0.71 | 0.53-0.96 | 0.02462474 |
| ZSCAN9_AT_75721 | 1.41 | 1.04-1.90 | 0.02462474 |
| ASNS_AA_80562 | 0.71 | 0.52-0.96 | 0.024655193 |
| NSMAF_ES_83944 | 0.71 | 0.52-0.96 | 0.02476183 |
| MEIS1_RI_53811 | 0.71 | 0.53-0.96 | 0.024766081 |
| TBRG1_ES_19225 | 0.71 | 0.52-0.96 | 0.024802149 |
| GATA3_AA_10728 | 0.71 | 0.53-0.96 | 0.024802375 |
| TMEM180_ES_12955 | 0.71 | 0.52-0.96 | 0.024848081 |
| OSBP2_AP_61803 | 0.71 | 0.53-0.96 | 0.024876594 |
| DNAJC5_ES_60176 | 0.71 | 0.53-0.96 | 0.024876972 |
| ZNF385A_AP_22176 | 1.41 | 1.04-1.90 | 0.025072968 |
| SEPT6_ES_89975 | 0.71 | 0.52-0.96 | 0.025120348 |
| C2orf81_RI_54053 | 0.71 | 0.53-0.96 | 0.025138416 |
| C16orf74_AP_37885 | 0.71 | 0.53-0.96 | 0.025153326 |
| CLN3_AP_35708 | 1.41 | 1.04-1.91 | 0.025195084 |
| FOXM1_ES_19721 | 1.41 | 1.04-1.90 | 0.025227097 |
| LIMS3L_AT_54936 | 1.41 | 1.04-1.91 | 0.025256124 |
| SERP2_ME_25779 | 1.41 | 1.04-1.90 | 0.025259122 |
| HAUS5_RI_49218 | 0.71 | 0.53-0.96 | 0.025262588 |
| KIF13A_ES_75457 | 0.71 | 0.53-0.96 | 0.025272802 |
| HOOK2_AD_47868 | 0.71 | 0.53-0.96 | 0.025276218 |
| LEKR1_AD_67374 | 1.41 | 1.04-1.90 | 0.025422409 |
| POLR2L_AT_13787 | 0.71 | 0.53-0.96 | 0.025440791 |
| POLR2L_AT_13788 | 1.41 | 1.04-1.90 | 0.025440791 |
| ALS2CL_RI_64461 | 0.71 | 0.53-0.96 | 0.025475531 |
| TMEM204_AP_33111 | 0.66 | 0.46-0.96 | 0.025497935 |
| MAGOHB_ES_20478 | 1.41 | 1.04-1.90 | 0.025505553 |
| FAM111A_ES_16028 | 0.71 | 0.53-0.96 | 0.025527279 |
| TREX1_RI_64682 | 1.41 | 1.04-1.90 | 0.025527607 |
| ARHGEF39_AP_86268 | 0.71 | 0.53-0.96 | 0.025533936 |
| ARHGEF39_AP_86269 | 1.41 | 1.04-1.90 | 0.025533936 |
| NAA38_RI_81579 | 0.71 | 0.53-0.96 | 0.025568542 |
| SPHK2_AP_50780 | 1.41 | 1.04-1.90 | 0.02557331 |
| DLG1_ES_68285 | 0.71 | 0.53-0.96 | 0.025573353 |
| PSMA4_AP_32101 | 0.71 | 0.53-0.96 | 0.025575426 |
| TCEA3_AP_1058 | 1.41 | 1.04-1.90 | 0.025585308 |
| ZNF655_ES_80688 | 0.71 | 0.53-0.96 | 0.025602616 |
| INTS3_RI_7758 | 0.71 | 0.53-0.96 | 0.0256061 |
| IL32_ES_33440 | 0.71 | 0.53-0.96 | 0.025611587 |
| ZNF430_AT_48741 | 1.41 | 1.04-1.90 | 0.025646278 |
| AP4M1_AT_80887 | 1.41 | 1.04-1.90 | 0.02565545 |
| AP4M1_AT_80889 | 0.71 | 0.53-0.96 | 0.02565545 |
| ENO2_AP_20011 | 0.71 | 0.53-0.96 | 0.025700926 |
| GUK1_AP_10181 | 0.71 | 0.53-0.96 | 0.025704002 |
| LRR1_ES_27423 | 0.71 | 0.52-0.96 | 0.025707938 |
| ZFYVE28_AP_68557 | 1.41 | 1.04-1.90 | 0.02581215 |
| SDHAF2_ES_16226 | 1.41 | 1.04-1.90 | 0.025815611 |
| HECTD3_RI_2592 | 0.71 | 0.53-0.96 | 0.02587713 |
| RPS20_AA_83888 | 0.71 | 0.53-0.96 | 0.02590007 |
| SMN1_AA_72422 | 0.71 | 0.53-0.96 | 0.025930498 |
| GDAP1_AD_84223 | 1.41 | 1.04-1.90 | 0.025937773 |
| IRF3_AD_50988 | 0.71 | 0.53-0.96 | 0.025952791 |
| TROVE2_AT_9253 | 1.41 | 1.04-1.90 | 0.025973257 |
| TROVE2_AT_9255 | 0.71 | 0.53-0.96 | 0.025973257 |
| RTN4_AP_53582 | 0.71 | 0.53-0.96 | 0.026011319 |
| TTC31_RI_54078 | 0.71 | 0.53-0.96 | 0.026021716 |
| C9orf89_RI_86899 | 0.71 | 0.53-0.96 | 0.026032356 |
| NUDT16L1_RI_33786 | 0.71 | 0.53-0.96 | 0.02605123 |
| SLC39A13_RI_15747 | 0.71 | 0.53-0.96 | 0.026081753 |
| TIA1_ES_53873 | 0.71 | 0.53-0.96 | 0.02608342 |
| KLHDC4_AT_37947 | 0.71 | 0.53-0.96 | 0.026105317 |
| FASTK_RI_82335 | 0.71 | 0.53-0.96 | 0.02610631 |
| FOXA1_ES_27334 | 0.71 | 0.53-0.96 | 0.026108927 |
| DDX55_AA_25094 | 0.71 | 0.53-0.96 | 0.026193681 |
| MFF_ES_57817 | 1.41 | 1.04-1.90 | 0.026199468 |
| MST1_RI_64899 | 0.71 | 0.53-0.96 | 0.026210816 |
| IQCK_AT_34333 | 0.71 | 0.53-0.96 | 0.02623195 |
| TMEM102_RI_38951 | 1.40 | 1.04-1.90 | 0.026240244 |
| ZNF76_RI_75899 | 0.71 | 0.53-0.96 | 0.02629496 |
| EPB41L2_AP_77529 | 1.40 | 1.04-1.89 | 0.026323377 |
| IFT20_AT_39878 | 1.40 | 1.04-1.90 | 0.02634437 |
| IFT20_AT_39879 | 0.71 | 0.53-0.96 | 0.02634437 |
| LGALS9_ES_39858 | 0.71 | 0.53-0.96 | 0.026384708 |
| C20orf96_AP_58436 | 1.40 | 1.04-1.90 | 0.026445288 |
| ABCB9_AP_24993 | 1.40 | 1.04-1.90 | 0.02646852 |
| TUBGCP2_AP_13530 | 0.71 | 0.53-0.96 | 0.026471812 |
| HOOK2_AP_47862 | 0.71 | 0.53-0.96 | 0.026491709 |
| PLCH2_ES_274 | 0.71 | 0.53-0.96 | 0.026533842 |
| ZNF655_AA_80669 | 0.71 | 0.53-0.96 | 0.026615087 |
| WDR53_ES_68249 | 1.40 | 1.04-1.90 | 0.026648525 |
| DDB1_AP_16154 | 0.71 | 0.53-0.96 | 0.026656743 |
| ANKRD13A_ME_250011 | 0.71 | 0.53-0.96 | 0.026701377 |
| ARL4C_RI_58078 | 1.40 | 1.04-1.90 | 0.026732303 |
| SEPT2_AP_58350 | 1.40 | 1.04-1.89 | 0.02675495 |
| ARHGEF18_AP_47101 | 0.71 | 0.53-0.96 | 0.026761308 |
| FAM195B_AA_44174 | 0.71 | 0.53-0.96 | 0.026786266 |
| UQCC1_AT_59083 | 0.71 | 0.53-0.96 | 0.026793793 |
| POLRMT_AD_46308 | 1.40 | 1.04-1.89 | 0.026804616 |
| RBM6_ES_64938 | 1.40 | 1.04-1.89 | 0.02683514 |
| BOP1_RI_85554 | 0.71 | 0.53-0.96 | 0.026838634 |
| RPE_ES_57239 | 0.71 | 0.53-0.96 | 0.026845459 |
| GJB2_AP_25419 | 1.40 | 1.04-1.89 | 0.026884756 |
| PDXP_AP_62143 | 0.71 | 0.53-0.96 | 0.02691112 |
| ZNF846_ES_47402 | 0.71 | 0.53-0.96 | 0.026977744 |
| DCAF8_AD_8451 | 1.40 | 1.04-1.89 | 0.026985283 |
| TNC_ES_87339 | 0.71 | 0.53-0.96 | 0.026996851 |
| EBAG9_AP_84898 | 0.71 | 0.53-0.96 | 0.027019628 |
| PABPC1L_AP_59490 | 1.40 | 1.04-1.90 | 0.027034215 |
| PABPC1L_AP_59491 | 0.71 | 0.53-0.96 | 0.027034215 |
| LRRC23_ES_20002 | 0.71 | 0.53-0.96 | 0.027044467 |
| FHAD1_AT_747 | 1.40 | 1.04-1.90 | 0.027049722 |
| SARNP_AT_22253 | 0.71 | 0.53-0.96 | 0.027052181 |
| PPIL2_AA_61242 | 0.71 | 0.53-0.96 | 0.027112223 |
| ZNF410_ES_28328 | 0.71 | 0.53-0.96 | 0.027135455 |
| RPUSD1_AP_33007 | 0.71 | 0.53-0.96 | 0.027138907 |
| CCNH_RI_72726 | 0.71 | 0.53-0.96 | 0.027150769 |
| ZNF160_AP_51649 | 0.71 | 0.53-0.96 | 0.027204842 |
| PIDD_RI_13768 | 0.71 | 0.53-0.96 | 0.027248476 |
| ADARB1_ES_60865 | 0.71 | 0.53-0.96 | 0.027289251 |
| ZCCHC17_ES_1464 | 0.71 | 0.53-0.96 | 0.027372571 |
| SNRNP70_ES_50886 | 0.71 | 0.53-0.96 | 0.027412068 |
| IFT20_ES_39882 | 0.71 | 0.53-0.96 | 0.027447215 |
| HEATR5A_AT_27113 | 1.40 | 1.04-1.89 | 0.02749772 |
| BRD9_ES_71461 | 0.71 | 0.53-0.96 | 0.02750757 |
| KLC1_AT_29467 | 0.71 | 0.53-0.96 | 0.027513034 |
| TMEM205_RI_47657 | 0.71 | 0.53-0.96 | 0.027535835 |
| PRELID2_ES_73898 | 1.40 | 1.04-1.89 | 0.027546546 |
| ZBTB7B_AP_7875 | 0.71 | 0.53-0.96 | 0.027548236 |
| ARHGAP6_AT_88478 | 0.71 | 0.52-0.97 | 0.027619787 |
| CARD14_AT_44026 | 0.71 | 0.53-0.97 | 0.027674005 |
| CARD14_AT_44027 | 1.40 | 1.04-1.89 | 0.027674005 |
| MRPL2_ES_76239 | 1.40 | 1.04-1.89 | 0.027680998 |
| ARF4_ES_65384 | 0.71 | 0.53-0.97 | 0.027682955 |
| ZNF3_AP_80875 | 1.40 | 1.04-1.90 | 0.027686646 |
| GNL3L_AD_89244 | 1.40 | 1.04-1.89 | 0.027688185 |
| CCDC74B_AT_55275 | 1.40 | 1.04-1.89 | 0.027708284 |
| SULT1A1_AP_35815 | 0.71 | 0.53-0.96 | 0.027728128 |
| NTPCR_ES_10319 | 1.40 | 1.04-1.89 | 0.027738656 |
| ANK3_AT_11846 | 0.71 | 0.53-0.97 | 0.027765399 |
| ANK3_AT_11847 | 1.40 | 1.04-1.89 | 0.027765399 |
| EXOC4_AP_81839 | 0.71 | 0.53-0.97 | 0.027772004 |
| TRAPPC6A_AD_50413 | 0.71 | 0.53-0.96 | 0.027795579 |
| NDRG1_ES_85246 | 0.71 | 0.53-0.96 | 0.027870948 |
| DST_ES_76560 | 1.40 | 1.04-1.89 | 0.027885022 |
| MCM7_RI_80883 | 0.71 | 0.53-0.97 | 0.027891149 |
| FLNB_ES_65420 | 0.71 | 0.53-0.97 | 0.02789255 |
| TUBB6_AT_44669 | 0.72 | 0.53-0.97 | 0.027906197 |
| ULK1_AP_25222 | 1.40 | 1.04-1.89 | 0.027926924 |
| CHORDC1_AA_18268 | 0.71 | 0.53-0.97 | 0.027950306 |
| HMGA2_AT_22881 | 0.71 | 0.53-0.97 | 0.027972091 |
| SERINC2_AP_1469 | 1.40 | 1.04-1.89 | 0.027995472 |
| SIRPA_AD_58536 | 0.72 | 0.53-0.97 | 0.028009458 |
| ROGDI_AD_33857 | 0.71 | 0.53-0.97 | 0.028051459 |
| C21orf59_AA_60358 | 0.72 | 0.53-0.97 | 0.028069558 |
| SPATS2L_AP_56732 | 1.40 | 1.04-1.89 | 0.028083861 |
| DMPK_ES_50522 | 0.71 | 0.53-0.97 | 0.028088296 |
| PMF1_ES_8202 | 0.71 | 0.53-0.97 | 0.028126781 |
| SSH3_AA_17168 | 1.40 | 1.04-1.89 | 0.028144631 |
| WRB_AD_60630 | 1.40 | 1.04-1.89 | 0.028144656 |
| ZNF530_AT_52303 | 0.71 | 0.53-0.97 | 0.028157329 |
| RBM19_RI_24647 | 0.71 | 0.53-0.97 | 0.028222831 |
| NFS1_ES_59219 | 0.72 | 0.53-0.97 | 0.028239611 |
| KIAA0101_ES_31114 | 0.72 | 0.53-0.97 | 0.028266276 |
| ARIH2_ES_64787 | 0.72 | 0.53-0.97 | 0.028287792 |
| HOMER3_AP_48535 | 1.40 | 1.03-1.89 | 0.028327687 |
| CNEP1R1_AP_36357 | 0.72 | 0.53-0.97 | 0.028366559 |
| KLC2_AP_16990 | 0.72 | 0.53-0.97 | 0.028380777 |
| ZBTB43_AP_87601 | 1.40 | 1.03-1.89 | 0.028437887 |
| PARP16_AA_31165 | 0.72 | 0.53-0.97 | 0.028440997 |
| LYRM5_AT_20809 | 1.40 | 1.03-1.89 | 0.028446487 |
| LYRM5_AT_20810 | 0.71 | 0.53-0.97 | 0.028446487 |
| NDRG1_ES_85251 | 0.71 | 0.52-0.97 | 0.028453465 |
| SRSF7_AD_53283 | 0.72 | 0.53-0.97 | 0.028493262 |
| PPP1R12A_AP_23526 | 0.72 | 0.53-0.97 | 0.028497262 |
| PIGQ_ES_32905 | 0.71 | 0.53-0.97 | 0.028515332 |
| ILK_AA_14177 | 1.40 | 1.03-1.89 | 0.028570119 |
| MCAM_AP_19134 | 0.72 | 0.53-0.97 | 0.028587314 |
| MCAM_AP_19135 | 1.40 | 1.03-1.89 | 0.028587314 |
| IRF7_RI_13707 | 0.72 | 0.53-0.97 | 0.028593475 |
| SYMPK_AP_50530 | 0.72 | 0.53-0.97 | 0.028608158 |
| SPATS2_AT_21577 | 0.72 | 0.53-0.97 | 0.028707661 |
| TMEM150A_ES_54306 | 0.72 | 0.53-0.97 | 0.028769968 |
| FAM110A_AP_58466 | 0.72 | 0.53-0.97 | 0.028847291 |
| ZNF558_AP_47259 | 1.40 | 1.03-1.89 | 0.028858763 |
| PABPN1_RI_26731 | 0.72 | 0.53-0.97 | 0.028873888 |
| USP40_AP_58043 | 1.40 | 1.03-1.88 | 0.028915613 |
| SIAH1_AP_36338 | 1.40 | 1.03-1.89 | 0.028924357 |
| ST7_AP_81553 | 0.72 | 0.53-0.97 | 0.028943965 |
| DRG2_RI_39549 | 0.72 | 0.53-0.97 | 0.028947136 |
| CAP1_AA_1985 | 1.40 | 1.03-1.89 | 0.028994331 |
| PPP1CC_AP_24497 | 1.40 | 1.03-1.88 | 0.028995349 |
| PPP1CC_AP_24498 | 0.72 | 0.53-0.97 | 0.028995349 |
| STRADB_AA_56840 | 0.72 | 0.53-0.97 | 0.029024728 |
| RASA4_ES_81122 | 0.72 | 0.53-0.97 | 0.029027774 |
| IL32_ES_33437 | 0.72 | 0.53-0.97 | 0.029031344 |
| FNBP1_AA_87882 | 1.40 | 1.03-1.89 | 0.029040271 |
| ATG16L2_AD_17651 | 0.72 | 0.53-0.97 | 0.029064517 |
| NAA40_AA_16552 | 0.72 | 0.53-0.97 | 0.029087488 |
| NIPSNAP3B_AT_87104 | 1.39 | 1.03-1.88 | 0.029176701 |
| RUNX2_AP_76398 | 0.72 | 0.53-0.97 | 0.029248641 |
| MDFIC_AP_81512 | 0.64 | 0.42-0.98 | 0.029327839 |
| ZNF10_AP_25338 | 0.72 | 0.53-0.97 | 0.029335244 |
| KLHL5_AT_69041 | 0.72 | 0.53-0.97 | 0.02934149 |
| RIN1_AP_17014 | 1.39 | 1.03-1.88 | 0.029377298 |
| CANT1_RI_43973 | 1.39 | 1.03-1.88 | 0.029399747 |
| LYNX1_AP_85361 | 1.39 | 1.03-1.88 | 0.029403456 |
| ST20_AP_32163 | 0.72 | 0.53-0.97 | 0.029455689 |
| C19orf47_ES_49882 | 0.72 | 0.53-0.97 | 0.029494817 |
| CYGB_AP_43589 | 0.72 | 0.53-0.97 | 0.029519612 |
| MRC2_AP_42908 | 0.72 | 0.53-0.97 | 0.029520648 |
| GTF2IRD2_AT_80091 | 0.72 | 0.53-0.97 | 0.029541665 |
| HYAL1_AP_64992 | 1.39 | 1.03-1.88 | 0.029569711 |
| ZBTB43_AP_87600 | 0.72 | 0.53-0.97 | 0.029569769 |
| SUOX_AP_22334 | 1.39 | 1.03-1.89 | 0.029586886 |
| FNBP1_AA_87880 | 1.39 | 1.03-1.88 | 0.029654612 |
| SPATS2_AT_21578 | 1.39 | 1.03-1.88 | 0.02968431 |
| UNKL_AD_33084 | 1.39 | 1.03-1.88 | 0.02969443 |
| C21orf49_AT_60379 | 0.72 | 0.53-0.97 | 0.029707638 |
| SHF_AT_30410 | 0.72 | 0.53-0.97 | 0.029792302 |
| SHF_AT_30411 | 1.39 | 1.03-1.88 | 0.029792302 |
| IQCK_AT_34334 | 1.39 | 1.03-1.89 | 0.029824293 |
| MOB3A_AP_46572 | 0.72 | 0.53-0.97 | 0.029874929 |
| ZNF345_AP_49423 | 0.72 | 0.53-0.97 | 0.029880711 |
| C7orf55-LUC7L2_ES_81960 | 0.72 | 0.53-0.97 | 0.029892296 |
| NABP1_AA_56613 | 0.72 | 0.53-0.97 | 0.030020174 |
| RNF123_AD_64907 | 0.72 | 0.53-0.97 | 0.030029893 |
| CASQ1_AP_8432 | 1.39 | 1.03-1.89 | 0.030037627 |
| ARHGEF9_AP_89301 | 1.39 | 1.03-1.89 | 0.030054356 |
| NDUFAF1_ES_30087 | 0.72 | 0.53-0.97 | 0.030062582 |
| P2RY6_AP_17684 | 1.39 | 1.03-1.88 | 0.030080195 |
| RBM6_ES_64952 | 0.72 | 0.53-0.97 | 0.030099421 |
| SRSF2_AD_43667 | 0.72 | 0.53-0.97 | 0.030130221 |
| PSEN2_AP_10024 | 1.39 | 1.03-1.88 | 0.030167108 |
| PLEKHG5_AP_467 | 1.39 | 1.03-1.88 | 0.030203528 |
| PTK2_AP_85291 | 1.39 | 1.03-1.89 | 0.03022623 |
| HERC4_AP_11912 | 0.72 | 0.53-0.97 | 0.030228441 |
| KDM5B_ES_9420 | 0.72 | 0.53-0.97 | 0.030247041 |
| EDEM2_AP_59064 | 0.72 | 0.53-0.97 | 0.030251524 |
| ASAH1_ES_82845 | 1.39 | 1.03-1.88 | 0.030257524 |
| TCF3_AP_46533 | 0.72 | 0.53-0.97 | 0.030269426 |
| RPAIN_ES_38691 | 1.39 | 1.03-1.88 | 0.030298194 |
| TOMM7_ES_78942 | 1.39 | 1.03-1.88 | 0.030327816 |
| BRF2_ES_83358 | 0.72 | 0.53-0.97 | 0.030391348 |
| CXorf40A_AD_90305 | 0.72 | 0.53-0.97 | 0.030405829 |
| TRPC4AP_ES_59059 | 1.39 | 1.03-1.88 | 0.030443559 |
| FGF11_AP_38954 | 1.39 | 1.03-1.88 | 0.030511675 |
| RAD21_AP_84981 | 0.72 | 0.53-0.97 | 0.030523625 |
| ANKS3_ES_33805 | 1.41 | 1.04-1.92 | 0.030571132 |
| SNED1_AT_58307 | 1.39 | 1.03-1.88 | 0.03058241 |
| SMIM19_AP_83737 | 0.72 | 0.53-0.97 | 0.030612413 |
| FBXO7_AP_61930 | 1.39 | 1.03-1.88 | 0.030625408 |
| TFEB_AT_76124 | 0.72 | 0.53-0.97 | 0.030641347 |
| SP140_AT_57871 | 1.39 | 1.03-1.88 | 0.030649672 |
| ANKRD13D_AP_17155 | 1.39 | 1.03-1.88 | 0.030650381 |
| ANKRD13D_AP_17156 | 0.72 | 0.53-0.97 | 0.030650381 |
| MFSD7_ES_68395 | 0.72 | 0.53-0.97 | 0.030696832 |
| PKM_ME_203434 | 1.39 | 1.03-1.88 | 0.030717256 |
| CAPS2_AT_23445 | 0.72 | 0.53-0.97 | 0.03072858 |
| SLC29A1_AP_76356 | 1.39 | 1.03-1.88 | 0.030769963 |
| RFX5_AP_7603 | 1.39 | 1.03-1.88 | 0.030853485 |
| DOK1_AP_54113 | 1.39 | 1.03-1.88 | 0.030906903 |
| NVL_ES_9944 | 0.72 | 0.53-0.97 | 0.030925873 |
| C19orf55_AT_49287 | 1.39 | 1.03-1.88 | 0.030952103 |
| C19orf55_AT_49288 | 0.72 | 0.53-0.97 | 0.030952103 |
| ZNF575_AP_50204 | 1.39 | 1.03-1.88 | 0.031020421 |
| TUBA1A_AP_21538 | 1.39 | 1.03-1.88 | 0.031021347 |
| DNAJC25_ES_87230 | 0.72 | 0.53-0.97 | 0.031038055 |
| PRKCZ_AP_240 | 1.39 | 1.03-1.88 | 0.031054375 |
| B4GALT4_RI_66285 | 0.72 | 0.53-0.97 | 0.031068062 |
| EEF1D_ES_85454 | 0.72 | 0.53-0.97 | 0.031107376 |
| MPZL1_ES_8875 | 1.39 | 1.03-1.88 | 0.03111007 |
| VPS13A_AP_86646 | 1.39 | 1.03-1.88 | 0.031133479 |
| DIDO1_AP_60089 | 0.72 | 0.53-0.97 | 0.031152858 |
| SPTB_AT_27908 | 1.39 | 1.03-1.88 | 0.031185827 |
| SPTB_AT_27909 | 0.72 | 0.53-0.97 | 0.031185827 |
| BRD1_AA_62751 | 1.39 | 1.03-1.88 | 0.031232244 |
| STX16_ES_59970 | 1.39 | 1.03-1.88 | 0.031241109 |
| NT5C2_ES_12992 | 0.72 | 0.53-0.97 | 0.03125673 |
| DOCK6_ES_47644 | 0.72 | 0.53-0.97 | 0.031258142 |
| HP1BP3_AP_936 | 1.39 | 1.03-1.88 | 0.031277933 |
| GOLGA8M_AT_29753 | 1.39 | 1.03-1.88 | 0.031298677 |
| CHTOP_ES_91128 | 0.72 | 0.53-0.97 | 0.031306586 |
| SUV420H1_AA_17297 | 0.72 | 0.53-0.97 | 0.031360235 |
| UAP1_AA_8749 | 1.39 | 1.03-1.87 | 0.031365881 |
| AFMID_ES_94692 | 0.72 | 0.53-0.97 | 0.031529553 |
| MTMR11_AP_7412 | 1.39 | 1.03-1.87 | 0.031538855 |
| QKI_AA_78410 | 0.72 | 0.53-0.97 | 0.031548086 |
| ASPHD1_AT_35984 | 1.39 | 1.03-1.87 | 0.031550775 |
| SEC24C_RI_12174 | 0.72 | 0.53-0.97 | 0.031563382 |
| PGRMC2_AP_70575 | 1.39 | 1.03-1.88 | 0.031567763 |
| SENP1_AP_21408 | 0.72 | 0.53-0.97 | 0.031635361 |
| PSMA4_ES_32107 | 1.39 | 1.03-1.88 | 0.031644793 |
| VPS18_ES_30060 | 0.72 | 0.53-0.97 | 0.031679852 |
| KDELC2_ES_18618 | 0.72 | 0.53-0.97 | 0.03168506 |
| ZGPAT_AD_60164 | 0.72 | 0.53-0.97 | 0.031749087 |
| ZNF625_AT_47781 | 0.72 | 0.53-0.97 | 0.031874113 |
| ZNF625_AT_47783 | 1.39 | 1.03-1.87 | 0.031874113 |
| ZNF93_AT_48695 | 0.72 | 0.53-0.97 | 0.031877999 |
| TRAF3_ES_29427 | 1.39 | 1.03-1.88 | 0.031889673 |
| TNK2_ES_68211 | 0.72 | 0.53-0.97 | 0.031970582 |
| NFKB2_AA_12948 | 1.39 | 1.03-1.87 | 0.032009907 |
| VKORC1_ES_36232 | 1.39 | 1.03-1.87 | 0.032020357 |
| OSGEP_AD_26442 | 0.72 | 0.53-0.97 | 0.032078784 |
| VEGFA_ES_76337 | 0.72 | 0.53-0.97 | 0.032108875 |
| CNOT2_RI_23371 | 0.72 | 0.53-0.97 | 0.032203676 |
| KLF8_AP_89288 | 1.39 | 1.03-1.88 | 0.032204728 |
| DAPL1_AT_55686 | 0.72 | 0.53-0.97 | 0.032221026 |
| EMR2_AT_48028 | 0.72 | 0.53-0.97 | 0.032274534 |
| EMR2_AT_48029 | 1.39 | 1.03-1.87 | 0.032274534 |
| BCL6_AP_68081 | 0.72 | 0.53-0.97 | 0.032288245 |
| BAK1_ES_75761 | 0.72 | 0.53-0.97 | 0.03230713 |
| KLHL21_RI_482 | 0.72 | 0.53-0.97 | 0.032326936 |
| FAM227B_AT_30539 | 1.39 | 1.03-1.87 | 0.03233181 |
| VPS45_ES_7421 | 0.72 | 0.53-0.97 | 0.032453587 |
| FOXP1_AP_65594 | 0.72 | 0.53-0.97 | 0.032456313 |
| SYVN1_AA_16795 | 0.72 | 0.53-0.97 | 0.032526577 |
| MTA1_ES_29647 | 0.72 | 0.53-0.97 | 0.032553338 |
| ZNF28_ES_51626 | 1.39 | 1.03-1.88 | 0.032555184 |
| L3MBTL2_ES_62412 | 0.72 | 0.53-0.97 | 0.032582648 |
| NRP1_AT_11198 | 1.39 | 1.03-1.87 | 0.03260581 |
| ADPRM_AD_39284 | 0.72 | 0.53-0.97 | 0.032652828 |
| LEPROTL1_AT_83272 | 0.72 | 0.53-0.97 | 0.032673602 |
| RWDD2A_RI_76867 | 0.72 | 0.53-0.97 | 0.03267915 |
| LRRC36_AT_37014 | 1.38 | 1.03-1.87 | 0.032704791 |
| G3BP2_AP_69549 | 0.72 | 0.53-0.97 | 0.032712288 |
| RAI14_ES_71716 | 0.72 | 0.53-0.98 | 0.032752125 |
| EWSR1_AT_61578 | 1.38 | 1.03-1.87 | 0.032759587 |
| EWSR1_AT_61579 | 0.72 | 0.54-0.97 | 0.032759587 |
| SPATA5L1_AD_30425 | 0.72 | 0.53-0.97 | 0.032767092 |
| PEX26_AT_61021 | 0.72 | 0.54-0.97 | 0.032776652 |
| PEX26_AT_61022 | 1.38 | 1.03-1.87 | 0.032776652 |
| ZFP1_AP_37584 | 0.72 | 0.54-0.97 | 0.032794098 |
| DGUOK_ES_95569 | 0.72 | 0.54-0.97 | 0.032822395 |
| NAALADL1_AT_16735 | 1.38 | 1.03-1.87 | 0.032840229 |
| NAALADL1_AT_16736 | 0.72 | 0.53-0.97 | 0.032840229 |
| ZMYM2_AT_25411 | 1.39 | 1.03-1.87 | 0.03285144 |
| ZMYM2_AT_25413 | 0.72 | 0.53-0.98 | 0.03285144 |
| ODF2L_AT_3671 | 1.39 | 1.03-1.87 | 0.032882572 |
| PLS3_AP_89920 | 1.38 | 1.03-1.87 | 0.032905907 |
| ENDOV_RI_44075 | 1.38 | 1.03-1.87 | 0.032922282 |
| ASAP2_ES_52611 | 1.38 | 1.03-1.87 | 0.032941881 |
| FBXO3_AP_14920 | 1.38 | 1.03-1.87 | 0.032945126 |
| PARD3_AT_11209 | 1.39 | 1.02-1.88 | 0.032979433 |
| CYP4F12_RI_48111 | 1.39 | 1.03-1.87 | 0.033047872 |
| FAM13A_AP_69903 | 1.39 | 1.02-1.87 | 0.033062912 |
| CCDC57_AP_44293 | 0.72 | 0.53-0.98 | 0.033095759 |
| KLHL5_AT_69042 | 1.38 | 1.02-1.87 | 0.03310593 |
| PHLDB2_AP_66058 | 1.39 | 1.03-1.87 | 0.033109324 |
| ARRB2_ES_38575 | 0.72 | 0.54-0.98 | 0.033126254 |
| ANLN_ES_79255 | 1.38 | 1.03-1.87 | 0.033127871 |
| IREB2_RI_32090 | 0.72 | 0.53-0.98 | 0.033182383 |
| TBX6_AT_36063 | 0.72 | 0.53-0.97 | 0.033204462 |
| TBX6_AT_36064 | 1.39 | 1.03-1.87 | 0.033204462 |
| MTHFSD_ES_37920 | 0.72 | 0.53-0.98 | 0.03324483 |
| ATG10_AT_72682 | 1.38 | 1.03-1.87 | 0.033248733 |
| PIGT_AD_59553 | 0.72 | 0.53-0.98 | 0.033302393 |
| MSL1_RI_40845 | 1.38 | 1.03-1.87 | 0.033309014 |
| VPS8_ES_67977 | 0.72 | 0.53-0.98 | 0.033313204 |
| TOP3B_RI_61266 | 0.72 | 0.54-0.98 | 0.033329799 |
| PIGG_AD_68359 | 1.38 | 1.03-1.87 | 0.033356781 |
| SERINC2_AP_1471 | 0.72 | 0.53-0.98 | 0.033358865 |
| CNN2_ES_46362 | 0.72 | 0.54-0.98 | 0.033435062 |
| EZH2_RI_82156 | 0.72 | 0.54-0.98 | 0.033444229 |
| SLC25A36_ES_67052 | 0.72 | 0.54-0.98 | 0.033469882 |
| ARMCX4_RI_89650 | 1.38 | 1.02-1.87 | 0.033487043 |
| AMIGO2_ES_21335 | 0.72 | 0.54-0.98 | 0.033498667 |
| SLMAP_ES_65394 | 1.38 | 1.02-1.87 | 0.033527219 |
| C20orf96_AP_58435 | 0.72 | 0.53-0.98 | 0.033530415 |
| GLYCTK_RI_65201 | 0.72 | 0.54-0.98 | 0.033546906 |
| C19orf82_AT_47375 | 1.38 | 1.02-1.87 | 0.033621947 |
| PARP2_AA_26427 | 1.38 | 1.02-1.87 | 0.033630368 |
| VEPH1_AT_67395 | 1.38 | 1.02-1.87 | 0.033655681 |
| GDPD5_AP_17855 | 1.38 | 1.02-1.87 | 0.033655815 |
| D2HGDH_RI_58415 | 1.38 | 1.02-1.87 | 0.033671809 |
| SNRNP70_ES_50888 | 0.72 | 0.54-0.98 | 0.033703896 |
| ULK3_ES_31768 | 0.72 | 0.54-0.98 | 0.033739978 |
| TMEM63B_AP_76351 | 1.38 | 1.02-1.87 | 0.033768462 |
| C18orf25_ES_45391 | 1.38 | 1.02-1.87 | 0.033774805 |
| FOXN3_AP_28789 | 1.39 | 1.02-1.87 | 0.033789816 |
| TRAPPC6A_ES_50411 | 0.72 | 0.54-0.98 | 0.033798634 |
| HIGD1B_AP_41896 | 0.72 | 0.53-0.98 | 0.033830701 |
| AP4M1_AP_80884 | 1.38 | 1.02-1.86 | 0.033872616 |
| AP4M1_AP_80885 | 0.72 | 0.54-0.98 | 0.033872616 |
| TMEM25_AP_19006 | 0.72 | 0.54-0.98 | 0.033880977 |
| SLC39A13_RI_15743 | 0.72 | 0.54-0.98 | 0.033900621 |
| CXADR_AT_60226 | 0.72 | 0.53-0.98 | 0.033959332 |
| CXADR_AT_60227 | 1.38 | 1.02-1.87 | 0.033959332 |
| CUX1_AT_81070 | 1.38 | 1.02-1.87 | 0.034003432 |
| CUX1_AT_81071 | 0.72 | 0.53-0.98 | 0.034003432 |
| SLC4A8_AT_21854 | 1.38 | 1.02-1.87 | 0.034060746 |
| EMP1_AA_20538 | 0.72 | 0.54-0.98 | 0.034078867 |
| YPEL3_AP_36068 | 1.38 | 1.02-1.87 | 0.034105146 |
| APBB3_RI_73662 | 0.72 | 0.54-0.98 | 0.03411138 |
| NUDT16L1_RI_33790 | 0.72 | 0.54-0.98 | 0.034129611 |
| KCTD7_AP_79878 | 1.38 | 1.02-1.86 | 0.034299464 |
| MASP2_AT_637 | 0.68 | 0.47-0.98 | 0.034320175 |
| NSMF_AP_88305 | 0.72 | 0.54-0.98 | 0.034325282 |
| TTC31_RI_54077 | 0.72 | 0.54-0.98 | 0.034361755 |
| ORC3_ES_76971 | 0.72 | 0.53-0.98 | 0.034377183 |
| PRKACB_AT_3587 | 1.38 | 1.02-1.87 | 0.034397839 |
| PRKACB_AT_3588 | 0.72 | 0.54-0.98 | 0.034397839 |
| FLAD1_ES_91157 | 0.72 | 0.54-0.98 | 0.034403755 |
| HEXIM2_AP_41949 | 0.72 | 0.54-0.98 | 0.034492685 |
| HEXIM2_AP_41950 | 1.38 | 1.02-1.86 | 0.034492685 |
| WDR83_AP_47816 | 0.72 | 0.54-0.98 | 0.03450433 |
| ADCK5_RI_85590 | 0.72 | 0.54-0.98 | 0.034553466 |
| LYRM4_AT_75241 | 1.38 | 1.02-1.86 | 0.034554179 |
| C1orf159_AA_21 | 0.72 | 0.54-0.98 | 0.034584976 |
| NDRG2_AD_26501 | 0.72 | 0.54-0.98 | 0.034597822 |
| MIPOL1_AT_27300 | 0.72 | 0.54-0.98 | 0.034619309 |
| TTC9C_ES_16416 | 0.72 | 0.54-0.98 | 0.034654696 |
| ASH1L_ES_8088 | 0.72 | 0.54-0.98 | 0.034659411 |
| HERC2_ES_29747 | 1.38 | 1.02-1.87 | 0.034660476 |
| DBF4B_AA_41887 | 1.38 | 1.02-1.86 | 0.034677627 |
| PGAP2_AD_14004 | 0.72 | 0.54-0.98 | 0.03468112 |
| SCAF11_AP_21316 | 1.38 | 1.02-1.86 | 0.034687185 |
| SAYSD1_AP_76036 | 1.38 | 1.02-1.87 | 0.034699715 |
| SAYSD1_AP_76037 | 0.72 | 0.54-0.98 | 0.034699715 |
| ZNF101_RI_48677 | 1.38 | 1.02-1.87 | 0.034725123 |
| TMUB2_ES_41789 | 0.72 | 0.54-0.98 | 0.034756103 |
| C19orf24_AP_46446 | 1.38 | 1.02-1.87 | 0.034767364 |
| C19orf24_AP_46447 | 0.72 | 0.54-0.98 | 0.034767364 |
| ABCC3_RI_42463 | 0.72 | 0.54-0.98 | 0.034799638 |
| VGLL4_ES_63399 | 0.72 | 0.54-0.98 | 0.034938016 |
| PTCD2_AD_72469 | 0.72 | 0.54-0.98 | 0.034968694 |
| MIB2_AD_198 | 1.38 | 1.02-1.86 | 0.034980164 |
| AACS_AP_25170 | 0.72 | 0.54-0.98 | 0.034981222 |
| GTF2H3_ES_25107 | 1.38 | 1.02-1.86 | 0.035004649 |
| VEGFA_ES_76329 | 0.72 | 0.53-0.98 | 0.035005996 |
| CXorf40B_AT_90327 | 1.38 | 1.02-1.87 | 0.035057464 |
| CXorf40B_AT_90328 | 0.72 | 0.54-0.98 | 0.035057464 |
| PLXNB1_AA_64642 | 1.38 | 1.02-1.86 | 0.035067333 |
| RTEL1_AD_60146 | 0.73 | 0.54-0.98 | 0.035092233 |
| LGALS1_ES_62144 | 0.72 | 0.54-0.98 | 0.035126309 |
| ZNF544_AT_52426 | 0.72 | 0.54-0.98 | 0.035203599 |
| GMFB_RI_27591 | 0.73 | 0.54-0.98 | 0.03527436 |
| SEPT2_AP_58351 | 0.73 | 0.54-0.98 | 0.03531979 |
| CCRL2_AP_64450 | 0.73 | 0.54-0.98 | 0.035339715 |
| MAF1_AP_85532 | 0.72 | 0.54-0.98 | 0.03537729 |
| ANKDD1A_AT_31138 | 0.73 | 0.54-0.98 | 0.035394813 |
| COA4_ES_17737 | 0.72 | 0.54-0.98 | 0.03548327 |
| MRPL55_AA_10083 | 0.73 | 0.54-0.98 | 0.035489063 |
| KIF9_ES_64501 | 1.38 | 1.02-1.87 | 0.035546275 |
| DDX19A_ES_37382 | 0.73 | 0.54-0.98 | 0.035558604 |
| VRK3_AT_51145 | 0.73 | 0.54-0.98 | 0.035598516 |
| VAV1_AP_47087 | 0.73 | 0.54-0.98 | 0.035643609 |
| TCTN1_ES_24483 | 0.73 | 0.54-0.98 | 0.035686382 |
| UBA52_AP_48467 | 0.73 | 0.54-0.98 | 0.035712741 |
| ZNF76_AA_75901 | 0.73 | 0.54-0.98 | 0.035770221 |
| BCL6_AP_68079 | 1.38 | 1.02-1.86 | 0.035811366 |
| PIP4K2C_ES_22652 | 0.72 | 0.54-0.98 | 0.035812368 |
| IDUA_AP_68440 | 1.38 | 1.02-1.86 | 0.035842264 |
| RAD1_AP_71736 | 0.73 | 0.54-0.98 | 0.035875985 |
| NMRAL1_AD_33737 | 1.38 | 1.02-1.87 | 0.035881155 |
| ZBTB44_AA_19496 | 1.38 | 1.02-1.86 | 0.035926395 |
| NSMF_ES_88325 | 0.72 | 0.53-0.98 | 0.035956435 |
| PIGH_AD_28076 | 0.73 | 0.54-0.98 | 0.035962032 |
| ZNF707_ES_85485 | 0.73 | 0.54-0.98 | 0.035963622 |
| ODF2L_AT_3668 | 0.73 | 0.54-0.98 | 0.035974403 |
| SLC35A5_AP_66110 | 0.73 | 0.54-0.98 | 0.03599992 |
| RHOT1_AD_40179 | 0.73 | 0.54-0.98 | 0.036024555 |
| BCCIP_AT_13431 | 1.38 | 1.02-1.86 | 0.036025151 |
| ARL16_ES_44149 | 0.73 | 0.54-0.98 | 0.03604965 |
| SPOP_ES_42316 | 0.73 | 0.54-0.98 | 0.036085392 |
| NRP2_AT_57107 | 0.73 | 0.54-0.98 | 0.036092113 |
| TRA2A_ES_78977 | 0.73 | 0.54-0.98 | 0.036136382 |
| URGCP_RI_79358 | 0.73 | 0.54-0.98 | 0.036178442 |
| ZNF692_AA_10568 | 0.73 | 0.54-0.98 | 0.036206814 |
| STAG3_ES_97624 | 0.73 | 0.54-0.98 | 0.036217486 |
| CLEC7A_AT_20310 | 1.38 | 1.02-1.86 | 0.036271241 |
| WTAP_AT_78312 | 0.73 | 0.54-0.98 | 0.036287958 |
| WTAP_AT_78313 | 1.38 | 1.02-1.87 | 0.036287958 |
| TMEM234_RI_1570 | 1.38 | 1.02-1.86 | 0.036353663 |
| EPS8L1_ES_52012 | 0.73 | 0.54-0.98 | 0.036382917 |
| RBMX_RI_90222 | 0.73 | 0.54-0.98 | 0.036482646 |
| SUOX_AP_22333 | 0.73 | 0.54-0.98 | 0.036494758 |
| IP6K2_AA_64754 | 0.73 | 0.54-0.98 | 0.036500746 |
| FAM49B_ES_85143 | 0.73 | 0.54-0.98 | 0.036577779 |
| SLC52A1_AP_38647 | 0.73 | 0.54-0.98 | 0.036593192 |
| SLC52A1_AP_38648 | 1.38 | 1.02-1.86 | 0.036593192 |
| TNPO2_AA_47853 | 1.38 | 1.02-1.86 | 0.036616303 |
| BRF1_AA_29614 | 0.73 | 0.54-0.98 | 0.036691156 |
| DERL3_AA_61333 | 0.73 | 0.54-0.98 | 0.036696941 |
| TRIM5_AD_14078 | 0.73 | 0.54-0.98 | 0.036718202 |
| U2AF1L4_ES_49272 | 0.73 | 0.54-0.98 | 0.036736709 |
| RPS24_ES_12295 | 0.73 | 0.54-0.98 | 0.036736754 |
| TUBGCP2_AP_13529 | 1.38 | 1.02-1.86 | 0.036753238 |
| EBAG9_AP_84897 | 1.38 | 1.02-1.86 | 0.036797695 |
| ADI1_AP_52562 | 1.38 | 1.02-1.86 | 0.036859948 |
| ADI1_AP_52563 | 0.73 | 0.54-0.98 | 0.036859948 |
| IDUA_RI_68442 | 0.73 | 0.54-0.98 | 0.036875177 |
| KNOP1_AP_34329 | 1.38 | 1.02-1.86 | 0.036877468 |
| TTC13_ME_10258 | 1.38 | 1.02-1.86 | 0.036897151 |
| BBS1_ES_17048 | 0.73 | 0.54-0.98 | 0.036921481 |
| ZNF799_ES_47799 | 0.73 | 0.54-0.98 | 0.037006238 |
| U2AF1L4_AA_49277 | 0.73 | 0.54-0.98 | 0.037056948 |
| SUPT20H_AA_25664 | 0.73 | 0.54-0.98 | 0.037118323 |
| AP1G2_AD_26773 | 0.73 | 0.54-0.98 | 0.037175814 |
| IFI6_AD_1337 | 0.73 | 0.54-0.98 | 0.037228389 |
| HERC4_AP_11911 | 1.37 | 1.02-1.85 | 0.037347832 |
| NFIC_ES_46679 | 0.73 | 0.54-0.98 | 0.037383295 |
| SPOP_ES_42315 | 0.73 | 0.54-0.98 | 0.037386317 |
| B3GNTL1_AP_44424 | 1.37 | 1.02-1.86 | 0.037392295 |
| NAE1_ES_36874 | 1.37 | 1.02-1.85 | 0.037411849 |
| TBL2_AA_79982 | 0.73 | 0.54-0.98 | 0.037435188 |
| PDLIM7_AA_74781 | 0.73 | 0.54-0.98 | 0.037455014 |
| DNMBP_AP_12781 | 1.37 | 1.02-1.85 | 0.037514722 |
| PIGT_ES_59575 | 1.37 | 1.02-1.85 | 0.037579489 |
| NRBP2_RI_85507 | 0.73 | 0.54-0.98 | 0.037761333 |
| TMEM8B_AP_86317 | 0.73 | 0.54-0.98 | 0.037772219 |
| SEC24D_AA_70448 | 1.37 | 1.02-1.85 | 0.037847322 |
| SCRN1_AP_79098 | 0.73 | 0.54-0.98 | 0.037849013 |
| GCOM1_AT_30798 | 0.73 | 0.54-0.98 | 0.037891792 |
| IL11RA_AP_86209 | 0.73 | 0.54-0.98 | 0.037908062 |
| EPHB2_RI_1026 | 0.73 | 0.54-0.98 | 0.037927553 |
| NDUFS2_RI_8597 | 0.73 | 0.54-0.98 | 0.037994763 |
| ZNF706_AA_84745 | 0.73 | 0.54-0.98 | 0.038005567 |
| SRSF7_RI_53276 | 0.73 | 0.54-0.98 | 0.038016104 |
| EIF1AD_ES_16977 | 1.37 | 1.02-1.85 | 0.038045961 |
| ZNF706_AD_84742 | 0.73 | 0.54-0.98 | 0.038100114 |
| TMPRSS6_AT_62085 | 1.38 | 1.02-1.86 | 0.038207571 |
| GBA_AP_8040 | 0.73 | 0.54-0.98 | 0.038214309 |
| LLGL2_AA_43462 | 0.73 | 0.54-0.98 | 0.03823472 |
| C19orf70_AT_46866 | 0.73 | 0.54-0.98 | 0.038247262 |
| C19orf70_AT_46867 | 1.37 | 1.02-1.85 | 0.038247262 |
| NFATC2IP_AP_35902 | 0.73 | 0.54-0.98 | 0.038289998 |
| GSTM4_AT_4050 | 1.37 | 1.02-1.85 | 0.038318339 |
| GSTM4_AT_4051 | 0.73 | 0.54-0.98 | 0.038318339 |
| MARK2_AP_16537 | 1.37 | 1.02-1.85 | 0.038340472 |
| SMIM4_AT_65229 | 0.73 | 0.54-0.98 | 0.03839937 |
| SMIM4_AT_65230 | 1.37 | 1.02-1.86 | 0.03839937 |
| ZNF655_ES_80664 | 0.73 | 0.54-0.98 | 0.038405566 |
| ENY2_AA_84887 | 0.73 | 0.54-0.98 | 0.0384181 |
| TM2D3_RI_32769 | 0.73 | 0.54-0.98 | 0.038432487 |
| ERCC1_AD_50447 | 0.73 | 0.54-0.98 | 0.03846251 |
| TMEM234_RI_1573 | 0.73 | 0.54-0.98 | 0.038471592 |
| PRPF38B_ES_3947 | 0.73 | 0.54-0.98 | 0.038489109 |
| SEC16A_ES_88173 | 1.37 | 1.02-1.85 | 0.038489598 |
| BEST3_AT_23329 | 1.37 | 1.02-1.86 | 0.038518996 |
| SMIM1_AT_338 | 0.73 | 0.54-0.98 | 0.038567948 |
| FAM124B_AT_57773 | 0.73 | 0.54-0.98 | 0.038587066 |
| ZSWIM7_AD_39412 | 0.73 | 0.54-0.98 | 0.038613166 |
| PLEKHG5_AP_466 | 0.73 | 0.54-0.99 | 0.03861903 |
| DUSP15_AT_58907 | 0.73 | 0.54-0.98 | 0.038647747 |
| SRSF11_AP_3370 | 1.37 | 1.01-1.85 | 0.038751838 |
| ATXN2L_AA_35852 | 1.37 | 1.02-1.86 | 0.038784964 |
| RASA4B_ES_81102 | 0.73 | 0.54-0.98 | 0.038818571 |
| ANGPT2_AT_82577 | 0.73 | 0.54-0.98 | 0.038827356 |
| ANGPT2_AT_82578 | 1.37 | 1.02-1.85 | 0.038827356 |
| ZCCHC9_RI_72669 | 0.73 | 0.54-0.99 | 0.038864252 |
| PML_ES_31664 | 0.73 | 0.54-0.98 | 0.038871462 |
| CXorf40A_AP_90298 | 1.37 | 1.01-1.86 | 0.038873332 |
| ZNF257_AT_48803 | 1.37 | 1.01-1.85 | 0.038957767 |
| ZNF257_AT_48804 | 0.73 | 0.54-0.99 | 0.038957767 |
| ESRP2_AA_37191 | 0.73 | 0.54-0.99 | 0.038993521 |
| PDHA1_AT_88632 | 1.37 | 1.02-1.85 | 0.039059164 |
| TMEM2_AP_86569 | 1.37 | 1.01-1.85 | 0.039086928 |
| AUH_AT_86822 | 1.37 | 1.01-1.85 | 0.039087107 |
| MTO1_ES_76748 | 0.73 | 0.54-0.99 | 0.039118395 |
| TANGO2_AD_61127 | 0.73 | 0.54-0.99 | 0.039219437 |
| LRTOMT_RI_17536 | 0.73 | 0.54-0.99 | 0.039247743 |
| GUSB_ES_79863 | 1.37 | 1.02-1.85 | 0.039249407 |
| ISY1_ES_66700 | 0.73 | 0.54-0.99 | 0.039277309 |
| INCENP_ES_16337 | 0.73 | 0.54-0.99 | 0.039280374 |
| HNRNPU_AD_10476 | 0.73 | 0.54-0.99 | 0.039293544 |
| STX1A_AD_80023 | 0.73 | 0.54-0.99 | 0.039295879 |
| SARNP_AT_22251 | 1.37 | 1.01-1.85 | 0.039300484 |
| FBLN1_AT_62665 | 0.73 | 0.54-0.99 | 0.039360004 |
| KCTD18_AD_56745 | 0.73 | 0.54-0.99 | 0.03942509 |
| TEX264_AD_65099 | 0.73 | 0.54-0.99 | 0.039452889 |
| SPHK1_AD_43585 | 1.37 | 1.01-1.85 | 0.039464761 |
| JKAMP_ES_27750 | 0.73 | 0.54-0.99 | 0.039503777 |
| MANEAL_ES_1836 | 0.73 | 0.54-0.99 | 0.03957031 |
| RTCA_ES_3878 | 0.73 | 0.54-0.99 | 0.039615102 |
| PSTK_ES_91868 | 0.73 | 0.54-0.99 | 0.0396919 |
| FAM173A_AA_32964 | 0.73 | 0.54-0.99 | 0.039700212 |
| ERI2_ES_34397 | 0.73 | 0.54-0.99 | 0.03977872 |
| AKAP8L_ES_48076 | 0.73 | 0.54-0.99 | 0.039794726 |
| MARCH7_AP_55700 | 1.37 | 1.01-1.85 | 0.039795201 |
| C21orf67_AT_60854 | 0.73 | 0.54-0.99 | 0.039802353 |
| DOK1_AP_54114 | 0.73 | 0.54-0.99 | 0.039803171 |
| ZBTB25_AP_27879 | 1.37 | 1.01-1.85 | 0.039814951 |
| NDUFB5_ES_67704 | 0.73 | 0.54-0.99 | 0.039838785 |
| SLC4A11_AP_58580 | 1.37 | 1.01-1.85 | 0.039852667 |
| PHB2_ES_20044 | 0.73 | 0.54-0.99 | 0.03986384 |
| TRMT11_ES_77439 | 0.73 | 0.54-0.99 | 0.03992914 |
| CECR5_AP_60967 | 1.37 | 1.01-1.85 | 0.040075832 |
| SERPINB8_ES_45738 | 1.37 | 1.01-1.85 | 0.040100736 |
| RBM6_RI_64936 | 0.73 | 0.54-0.99 | 0.040160279 |
| CARD8_AT_50706 | 1.37 | 1.01-1.84 | 0.040237828 |
| ATXN2L_RI_35854 | 0.73 | 0.54-0.99 | 0.040256104 |
| POFUT2_AA_60871 | 0.73 | 0.54-0.99 | 0.040292099 |
| PIGG_ES_68369 | 0.73 | 0.54-0.99 | 0.040336237 |
| PPARG_AP_63415 | 0.73 | 0.54-0.99 | 0.040368651 |
| YIF1B_AT_49606 | 0.73 | 0.54-0.99 | 0.040371873 |
| YIF1B_AT_49607 | 1.37 | 1.01-1.85 | 0.040371873 |
| TSPAN4_AP_13793 | 0.73 | 0.54-0.99 | 0.040374298 |
| METTL17_RI_26477 | 0.73 | 0.54-0.99 | 0.040402819 |
| GALT_AA_86199 | 0.73 | 0.54-0.99 | 0.040440256 |
| ZNF263_ES_33511 | 0.73 | 0.54-0.99 | 0.040459071 |
| CANT1_ES_43974 | 0.73 | 0.54-0.99 | 0.040468917 |
| MIA3_AP_9887 | 1.37 | 1.01-1.85 | 0.040495899 |
| SCPEP1_ES_94617 | 0.73 | 0.54-0.99 | 0.040497462 |
| SRSF2_RI_43663 | 0.73 | 0.54-0.99 | 0.040525218 |
| DMPK_ES_50523 | 0.73 | 0.54-0.99 | 0.040528841 |
| C16orf13_ES_32915 | 0.73 | 0.54-0.99 | 0.040551144 |
| VAMP3_AP_509 | 1.37 | 1.01-1.84 | 0.040587332 |
| VAMP3_AP_510 | 0.73 | 0.54-0.99 | 0.040587332 |
| IL1RL1_AT_54791 | 1.37 | 1.01-1.85 | 0.040588079 |
| D2HGDH_ES_58414 | 0.73 | 0.54-0.99 | 0.040589429 |
| PCNXL2_AP_10321 | 1.37 | 1.01-1.85 | 0.040616962 |
| TUBB3_ES_38175 | 0.73 | 0.54-0.99 | 0.040652478 |
| MLTK_AT_55998 | 1.37 | 1.01-1.85 | 0.040667252 |
| MLTK_AT_55999 | 0.73 | 0.54-0.99 | 0.040667252 |
| VDAC1_AP_73334 | 1.37 | 1.01-1.85 | 0.040669666 |
| VDAC1_AP_73335 | 0.73 | 0.54-0.99 | 0.040669666 |
| TFEB_AT_76123 | 1.37 | 1.01-1.84 | 0.040681752 |
| CGGBP1_RI_65667 | 0.73 | 0.54-0.99 | 0.040702834 |
| ZNF83_AP_51474 | 0.73 | 0.54-0.99 | 0.04074075 |
| HDAC6_RI_89011 | 0.73 | 0.54-0.99 | 0.040844832 |
| MVD_ES_38010 | 0.73 | 0.54-0.99 | 0.040851976 |
| TTC21B_AT_55811 | 1.37 | 1.01-1.85 | 0.040882666 |
| TTC21B_AT_55812 | 0.73 | 0.54-0.99 | 0.040882666 |
| RAPH1_AT_57075 | 0.73 | 0.54-0.99 | 0.040884292 |
| C1S_AP_20067 | 1.37 | 1.01-1.85 | 0.040887418 |
| C1S_AP_20068 | 0.73 | 0.54-0.99 | 0.040887418 |
| EP400NL_AP_25237 | 0.73 | 0.54-0.99 | 0.040893109 |
| POT1_ES_81644 | 0.73 | 0.54-0.99 | 0.040912792 |
| NME6_ES_64588 | 0.73 | 0.54-0.99 | 0.040939776 |
| CHORDC1_AP_18260 | 1.37 | 1.01-1.84 | 0.040948418 |
| ZFYVE19_ES_30050 | 0.73 | 0.54-0.99 | 0.040988703 |
| PHLDB1_ES_19040 | 1.37 | 1.01-1.85 | 0.041052776 |
| CCDC64B_AP_33364 | 0.73 | 0.54-0.99 | 0.041099929 |
| THOC1_ES_44445 | 0.73 | 0.54-0.99 | 0.041112614 |
| NCOR2_AA_25147 | 0.73 | 0.54-0.99 | 0.041149455 |
| TNFRSF12A_AD_33349 | 0.73 | 0.54-0.99 | 0.041152089 |
| WBP1_ES_54068 | 0.73 | 0.54-0.99 | 0.041178044 |
| ZNF74_ES_61153 | 0.73 | 0.54-0.99 | 0.041223162 |
| ZNF43_AP_48789 | 0.73 | 0.54-0.99 | 0.041334768 |
| FAM134C_AP_41082 | 0.73 | 0.54-0.99 | 0.041376001 |
| FAM134C_AP_41083 | 1.37 | 1.01-1.85 | 0.041376001 |
| ZWINT_RI_11813 | 0.73 | 0.54-0.99 | 0.041400188 |
| DDX46_ES_73426 | 0.73 | 0.54-0.99 | 0.041453645 |
| ATE1_AP_13319 | 0.73 | 0.54-0.99 | 0.041469841 |
| ZNF28_AP_51623 | 0.73 | 0.54-0.99 | 0.041502515 |
| NARF_ES_44402 | 0.73 | 0.54-0.99 | 0.041524829 |
| SECISBP2_AP_86796 | 1.36 | 1.01-1.84 | 0.041527119 |
| SNCA_AT_69928 | 0.73 | 0.54-0.99 | 0.041534891 |
| COX6C_AT_84676 | 1.37 | 1.01-1.85 | 0.041548418 |
| ICMT_ES_385 | 1.37 | 1.01-1.85 | 0.041549614 |
| CD58_AA_4363 | 0.73 | 0.54-0.99 | 0.041551732 |
| CAMKK2_AP_24845 | 0.73 | 0.54-0.99 | 0.041574913 |
| SLC25A3_AD_23856 | 0.73 | 0.54-0.99 | 0.04159194 |
| U2SURP_ES_67125 | 1.37 | 1.01-1.84 | 0.041658126 |
| ZNF577_AT_51377 | 1.36 | 1.01-1.84 | 0.041660818 |
| ZMIZ2_ES_79559 | 0.73 | 0.54-0.99 | 0.041704365 |
| NPIPB5_AP_35559 | 1.36 | 1.01-1.84 | 0.041744245 |
| TRIM5_RI_14074 | 0.73 | 0.54-0.99 | 0.041790711 |
| RIMKLB_AT_20202 | 1.36 | 1.01-1.84 | 0.041811228 |
| RIMKLB_AT_20203 | 0.73 | 0.54-0.99 | 0.041811228 |
| UNC119_RI_39915 | 0.73 | 0.54-0.99 | 0.041859858 |
| NFX1_AT_86100 | 1.36 | 1.01-1.84 | 0.041874196 |
| RTN4_AP_53584 | 1.37 | 1.01-1.85 | 0.041922135 |
| HEMK1_AD_65084 | 1.36 | 1.01-1.84 | 0.041927802 |
| METTL23_ES_43635 | 1.36 | 1.01-1.84 | 0.041928398 |
| HPS5_ES_14591 | 0.73 | 0.54-0.99 | 0.041956706 |
| METTL10_AD_13408 | 1.36 | 1.01-1.84 | 0.04198794 |
| POLM_RI_79452 | 0.73 | 0.54-0.99 | 0.042024659 |
| FBXO7_AP_61931 | 0.73 | 0.54-0.99 | 0.042050981 |
| WAC_AP_11098 | 1.36 | 1.01-1.84 | 0.042152193 |
| ERICH1_AT_82553 | 1.36 | 1.01-1.84 | 0.042189455 |
| ERICH1_AT_82554 | 0.73 | 0.54-0.99 | 0.042189455 |
| LMBR1L_ES_21522 | 0.73 | 0.54-0.99 | 0.042244067 |
| SHPK_AT_38462 | 0.73 | 0.54-0.99 | 0.042265172 |
| OCIAD1_AD_69252 | 0.73 | 0.54-0.99 | 0.042267239 |
| SNX19_AP_19502 | 0.73 | 0.54-0.99 | 0.04227695 |
| MOGS_RI_54069 | 0.73 | 0.54-0.99 | 0.042327403 |
| ATP6V0A2_AP_25112 | 1.36 | 1.01-1.84 | 0.042400901 |
| THOP1_AP_46623 | 0.73 | 0.54-0.99 | 0.042429419 |
| ABCC5_AA_67823 | 0.73 | 0.54-0.99 | 0.0424504 |
| RRNAD1_RI_8313 | 0.73 | 0.54-0.99 | 0.042500638 |
| SLC25A29_AP_29250 | 1.36 | 1.01-1.84 | 0.042531919 |
| EXOC6_AP_12540 | 0.73 | 0.54-0.99 | 0.042568769 |
| MRPL48_AP_17713 | 0.73 | 0.54-0.99 | 0.042575502 |
| MRPL48_AP_17714 | 1.36 | 1.01-1.84 | 0.042575502 |
| TPM1_ES_30997 | 0.73 | 0.54-0.99 | 0.04262402 |
| ZNF773_RI_52280 | 0.73 | 0.54-0.99 | 0.042645042 |
| CYTH1_AP_43888 | 0.73 | 0.53-0.99 | 0.042682806 |
| XRN1_ES_67112 | 0.73 | 0.54-0.99 | 0.042690944 |
| KIAA0825_AT_72791 | 0.73 | 0.54-0.99 | 0.042725244 |
| TNKS_AP_82594 | 0.73 | 0.54-0.99 | 0.042776872 |
| TNKS_AP_82595 | 1.36 | 1.01-1.84 | 0.042776872 |
| RUVBL2_ES_50861 | 0.73 | 0.54-0.99 | 0.042778454 |
| PTDSS2_ES_13660 | 0.73 | 0.54-0.99 | 0.042781987 |
| PMM1_ES_62439 | 0.73 | 0.54-0.99 | 0.042851769 |
| NIPSNAP3B_AT_87103 | 0.73 | 0.54-0.99 | 0.042860372 |
| MEIS1_AT_53809 | 0.73 | 0.54-0.99 | 0.042959757 |
| HSPD1_AP_56688 | 1.36 | 1.01-1.84 | 0.042988776 |
| RAB15_AD_27925 | 0.73 | 0.54-0.99 | 0.043135968 |
| ATP5G3_RI_56093 | 0.73 | 0.54-0.99 | 0.043148458 |
| RPL27A_ES_91937 | 0.73 | 0.54-0.99 | 0.043152411 |
| HSD11B1L_ES_46911 | 0.73 | 0.54-0.99 | 0.043153022 |
| DIP2A_AT_60940 | 1.36 | 1.01-1.84 | 0.043163971 |
| NPIPB9_AP_94101 | 1.36 | 1.01-1.84 | 0.043177061 |
| GUK1_AP_10178 | 1.36 | 1.01-1.84 | 0.043201746 |
| KMT2C_AP_82412 | 1.36 | 1.01-1.84 | 0.043202739 |
| KMT2C_AP_82413 | 0.73 | 0.54-0.99 | 0.043202739 |
| TMEM8B_AP_86318 | 1.36 | 1.01-1.84 | 0.043205361 |
| HMGA1_ES_75775 | 0.73 | 0.54-0.99 | 0.043235578 |
| DLG4_AP_38844 | 0.73 | 0.54-0.99 | 0.043289389 |
| SCO2_AP_62846 | 1.36 | 1.01-1.85 | 0.043297249 |
| PLA2G16_AP_16516 | 0.73 | 0.54-0.99 | 0.043337001 |
| SRSF6_ES_59434 | 0.73 | 0.54-0.99 | 0.043345658 |
| C12orf73_AP_24066 | 1.36 | 1.01-1.84 | 0.043358421 |
| TBC1D22A_AP_62720 | 0.73 | 0.54-0.99 | 0.043364399 |
| NDRG1_ES_85243 | 0.72 | 0.53-1.00 | 0.04336692 |
| CAPN1_AP_16799 | 0.73 | 0.54-0.99 | 0.043377234 |
| NFIX_ES_47907 | 0.73 | 0.54-0.99 | 0.043436813 |
| LPAR2_ES_48666 | 0.73 | 0.54-0.99 | 0.043441977 |
| ATP10D_AT_69176 | 0.73 | 0.54-0.99 | 0.043443784 |
| ATP10D_AT_69177 | 1.36 | 1.01-1.84 | 0.043443784 |
| SLC44A3_ES_3821 | 0.74 | 0.54-0.99 | 0.04346134 |
| TGFBR2_ES_63806 | 1.36 | 1.01-1.84 | 0.043475332 |
| MIF4GD_ES_43425 | 1.36 | 1.01-1.83 | 0.043476345 |
| KIAA0141_RI_73836 | 0.74 | 0.54-0.99 | 0.043508643 |
| DPP9_ES_46825 | 0.73 | 0.54-0.99 | 0.043554299 |
| UBE3D_AT_76841 | 1.36 | 1.01-1.83 | 0.043555778 |
| PIEZO1_ES_38024 | 0.74 | 0.54-0.99 | 0.043619525 |
| HSPB11_AT_3097 | 1.36 | 1.01-1.84 | 0.043621726 |
| RNASE1_ES_26469 | 0.73 | 0.54-0.99 | 0.04366669 |
| MOK_AT_29368 | 0.73 | 0.54-0.99 | 0.043790237 |
| ANKRD65_RI_170 | 1.36 | 1.01-1.84 | 0.043846881 |
| ARMC5_RI_36260 | 0.74 | 0.55-0.99 | 0.043862121 |
| MAP2K7_ES_47194 | 1.36 | 1.01-1.84 | 0.04387356 |
| CLK1_AP_56751 | 1.36 | 1.01-1.84 | 0.043908633 |
| ATP5J_ES_60265 | 1.36 | 1.01-1.84 | 0.043909264 |
| ZFP1_AP_37585 | 1.36 | 1.01-1.84 | 0.043911461 |
| DCLRE1C_AP_10835 | 0.74 | 0.54-0.99 | 0.043968999 |
| INADL_ES_3245 | 1.36 | 1.01-1.83 | 0.043995383 |
| SKA2_ES_42751 | 0.74 | 0.54-0.99 | 0.044001743 |
| PSMG3_AP_78591 | 1.36 | 1.01-1.83 | 0.044003279 |
| SPATA33_AP_38104 | 1.36 | 1.01-1.83 | 0.044040129 |
| FDPS_ES_8059 | 1.36 | 1.01-1.84 | 0.044071683 |
| TMEM107_ES_39117 | 0.74 | 0.54-0.99 | 0.044083156 |
| NFKBIB_RI_49718 | 1.36 | 1.01-1.84 | 0.04408339 |
| C19orf82_ES_47383 | 1.36 | 1.01-1.83 | 0.044084896 |
| ABCA2_RI_88247 | 0.74 | 0.54-0.99 | 0.044140105 |
| EFCAB14_AT_2823 | 1.36 | 1.01-1.84 | 0.044160936 |
| EFCAB14_AT_2824 | 0.74 | 0.54-0.99 | 0.044160936 |
| PRPF39_ES_27398 | 0.74 | 0.54-0.99 | 0.044187982 |
| PPP6R2_ES_62825 | 0.74 | 0.54-0.99 | 0.044259689 |
| ATP5J2_AD_80645 | 1.36 | 1.01-1.84 | 0.044272287 |
| CCDC91_ES_20916 | 0.74 | 0.54-0.99 | 0.044287528 |
| POFUT2_RI_60870 | 0.74 | 0.54-0.99 | 0.044297589 |
| ST7L_AT_4205 | 1.36 | 1.01-1.84 | 0.044336461 |
| ST7L_AT_4207 | 0.74 | 0.54-0.99 | 0.044336461 |
| PCNXL2_AP_10320 | 0.74 | 0.54-0.99 | 0.044337203 |
| MRPL55_RI_10072 | 0.74 | 0.55-0.99 | 0.044374388 |
| SERPINH1_AP_17867 | 0.74 | 0.55-0.99 | 0.044404161 |
| C11orf68_AA_16949 | 1.36 | 1.01-1.84 | 0.044415348 |
| AFMID_ES_43804 | 0.74 | 0.54-0.99 | 0.044452395 |
| TMEM62_ES_30214 | 1.36 | 1.01-1.83 | 0.044460845 |
| MGAT4A_AT_54653 | 1.36 | 1.01-1.84 | 0.044500232 |
| MYO6_ES_76805 | 1.36 | 1.01-1.83 | 0.044541303 |
| RIN1_AP_17013 | 0.74 | 0.55-0.99 | 0.044545971 |
| D2HGDH_AA_58424 | 1.36 | 1.01-1.83 | 0.044578066 |
| MIA3_AP_9888 | 0.74 | 0.54-0.99 | 0.044585452 |
| MRPL55_RI_10081 | 0.74 | 0.55-0.99 | 0.044587535 |
| REEP5_ES_72992 | 0.74 | 0.55-0.99 | 0.044623418 |
| SLC9B2_AP_70168 | 1.40 | 1.02-1.93 | 0.044647007 |
| ZNF3_AP_80874 | 0.74 | 0.54-0.99 | 0.044663315 |
| RNF19A_AP_84704 | 0.74 | 0.55-0.99 | 0.044690543 |
| LYSMD1_AD_7566 | 0.74 | 0.54-0.99 | 0.044756008 |
| EIF3G_ES_47465 | 0.74 | 0.55-0.99 | 0.044769504 |
| SEC16A_AP_88169 | 1.36 | 1.01-1.83 | 0.044811922 |
| SEC16A_AP_88170 | 0.74 | 0.55-0.99 | 0.044811922 |
| PIGB_ES_30721 | 1.36 | 1.01-1.83 | 0.044836186 |
| GINS4_AT_83515 | 1.36 | 1.01-1.83 | 0.04488068 |
| GINS4_AT_83516 | 0.74 | 0.55-0.99 | 0.04488068 |
| ZNF706_AP_84735 | 1.36 | 1.01-1.84 | 0.04488398 |
| RAD51C_AD_42719 | 1.36 | 1.00-1.84 | 0.044906067 |
| CLINT1_AA_74394 | 0.74 | 0.54-0.99 | 0.044928284 |
| SERPINF1_AA_38372 | 0.74 | 0.54-0.99 | 0.044968938 |
| PQLC1_ES_46257 | 1.36 | 1.01-1.84 | 0.045030439 |
| MFSD9_AA_54806 | 0.74 | 0.55-0.99 | 0.0450709 |
| DCLRE1C_AP_10836 | 1.36 | 1.01-1.83 | 0.045093532 |
| PSTPIP1_AT_31964 | 0.74 | 0.55-0.99 | 0.045097946 |
| C19orf60_AA_48492 | 0.74 | 0.55-0.99 | 0.045106153 |
| ABHD17A_AD_46558 | 0.74 | 0.55-0.99 | 0.045121345 |
| TDP2_AD_75528 | 0.74 | 0.55-0.99 | 0.045148573 |
| C16orf13_ES_32917 | 0.73 | 0.54-1.00 | 0.045187901 |
| ACYP2_AP_53563 | 1.36 | 1.01-1.83 | 0.045204869 |
| C15orf27_AT_31921 | 1.36 | 1.01-1.83 | 0.045243117 |
| C15orf27_AT_31922 | 0.74 | 0.55-0.99 | 0.045243117 |
| NEIL2_ES_82635 | 0.74 | 0.55-0.99 | 0.04532329 |
| MBD4_AT_66717 | 1.36 | 1.01-1.83 | 0.045324039 |
| MBD4_AT_66718 | 0.74 | 0.55-0.99 | 0.045324039 |
| JKAMP_AA_27751 | 0.73 | 0.54-1.00 | 0.045361354 |
| ACAA1_ES_64022 | 1.36 | 1.00-1.83 | 0.045380497 |
| CCDC74A_RI_55387 | 0.74 | 0.55-1.00 | 0.045393265 |
| AFMID_ES_43805 | 0.74 | 0.55-1.00 | 0.045412992 |
| DCTD_ES_71248 | 0.74 | 0.55-0.99 | 0.045425343 |
| ZNF430_AT_48742 | 0.74 | 0.55-1.00 | 0.04553451 |
| ZNF276_AP_38137 | 0.74 | 0.54-1.00 | 0.04554322 |
| ZNF763_AP_47763 | 1.36 | 1.00-1.84 | 0.045659571 |
| BTN2A2_AA_75643 | 0.74 | 0.55-0.99 | 0.045675313 |
| NPIPB4_RI_35515 | 1.36 | 1.00-1.83 | 0.045890002 |
| ATP9B_AT_46230 | 1.36 | 1.00-1.83 | 0.045969079 |
| ATP9B_AT_46232 | 0.74 | 0.55-1.00 | 0.045969079 |
| C1orf63_AA_1146 | 0.74 | 0.54-1.00 | 0.045980219 |
| CDIP1_AP_33755 | 1.36 | 1.00-1.83 | 0.046007395 |
| ZNF195_ES_13981 | 0.74 | 0.55-1.00 | 0.046089206 |
| ITPRIP_AP_13057 | 1.36 | 1.01-1.84 | 0.046119142 |
| TFAP2A_AP_75292 | 0.74 | 0.55-1.00 | 0.046160079 |
| ZNF841_AP_51405 | 0.74 | 0.55-1.00 | 0.046225635 |
| HERPUD2_RI_79234 | 1.36 | 1.00-1.83 | 0.046376423 |
| IQCH_AT_31311 | 0.74 | 0.55-1.00 | 0.046378358 |
| SERPINB8_ES_45739 | 1.36 | 1.00-1.83 | 0.046425153 |
| ATP2C1_AD_66762 | 1.36 | 1.00-1.83 | 0.046438038 |
| RAD1_AP_71734 | 1.35 | 1.00-1.83 | 0.04644433 |
| SPHK2_AP_50779 | 0.74 | 0.55-1.00 | 0.046453484 |
| SLC25A16_ES_11952 | 0.74 | 0.55-1.00 | 0.046497626 |
| ATE1_AP_13320 | 1.36 | 1.00-1.83 | 0.046498555 |
| DMPK_AA_50521 | 1.36 | 1.00-1.83 | 0.046509341 |
| ZNF333_AT_48017 | 1.36 | 1.00-1.83 | 0.046516209 |
| MKNK1_ES_2809 | 0.74 | 0.55-1.00 | 0.046552093 |
| ENOSF1_ES_44466 | 1.35 | 1.00-1.83 | 0.04656049 |
| HAUS8_AP_48222 | 0.74 | 0.55-1.00 | 0.04656108 |
| WDR26_AA_9962 | 0.74 | 0.55-1.00 | 0.046570526 |
| CCDC28B_AT_1560 | 0.74 | 0.55-1.00 | 0.046572379 |
| CCDC28B_AT_1561 | 1.36 | 1.00-1.83 | 0.046572379 |
| MCF2L_AP_26318 | 0.74 | 0.55-1.00 | 0.046579287 |
| GSTA4_AP_76478 | 1.35 | 1.00-1.83 | 0.046614294 |
| UBA1_AP_88907 | 1.36 | 1.00-1.83 | 0.046652278 |
| HAUS1_ES_45389 | 0.74 | 0.55-1.00 | 0.046769982 |
| GCOM1_AT_30799 | 1.35 | 1.00-1.83 | 0.046786922 |
| HNRNPLL_AT_53259 | 1.35 | 1.00-1.83 | 0.046799731 |
| C7orf43_AP_80904 | 1.35 | 1.00-1.83 | 0.046826984 |
| C7orf43_AP_80905 | 0.74 | 0.55-1.00 | 0.046826984 |
| SH3TC2_AP_74001 | 1.35 | 1.00-1.83 | 0.046947586 |
| SSH3_AA_17161 | 1.35 | 1.00-1.83 | 0.046987071 |
| TARDBP_ES_635 | 0.74 | 0.55-1.00 | 0.046998046 |
| MYL6_RI_22385 | 0.74 | 0.55-1.00 | 0.047010084 |
| SPG21_AP_31149 | 0.74 | 0.55-1.00 | 0.047034688 |
| TMEM150A_ES_54304 | 0.74 | 0.55-1.00 | 0.047041776 |
| CRELD1_RI_63293 | 0.74 | 0.55-1.00 | 0.047172448 |
| DDT_AP_61357 | 1.35 | 1.00-1.83 | 0.047186794 |
| DTX3_ES_22662 | 0.74 | 0.55-1.00 | 0.047249335 |
| CCDC57_AP_44294 | 1.35 | 1.00-1.83 | 0.047259608 |
| FCHSD1_ES_73817 | 0.74 | 0.55-1.00 | 0.047347906 |
| ITFG2_AD_19717 | 0.74 | 0.55-1.00 | 0.047396747 |
| RANGRF_RI_39168 | 0.74 | 0.55-1.00 | 0.047455153 |
| CDADC1_ES_25877 | 0.74 | 0.55-1.00 | 0.047460036 |
| MAP7D1_RI_1760 | 1.35 | 1.00-1.83 | 0.047495013 |
| GJB6_AP_25422 | 0.74 | 0.55-1.00 | 0.047497492 |
| AKAP1_ES_42609 | 0.74 | 0.55-1.00 | 0.047515038 |
| MEIS2_AP_29906 | 1.35 | 1.00-1.83 | 0.047572574 |
| C1orf63_AP_1140 | 0.74 | 0.55-1.00 | 0.047584003 |
| C1orf63_AP_1141 | 1.35 | 1.00-1.83 | 0.047584003 |
| HYAL1_AP_64993 | 0.74 | 0.55-1.00 | 0.047585467 |
| TMEM185A_AT_90319 | 1.35 | 1.00-1.83 | 0.047595383 |
| FAM45A_AA_13256 | 0.74 | 0.55-1.00 | 0.047630912 |
| TADA2B_AP_68732 | 1.35 | 1.00-1.83 | 0.047710676 |
| TMUB2_AD_41816 | 1.35 | 1.00-1.83 | 0.047724005 |
| TNFRSF1A_AT_19827 | 1.35 | 1.00-1.83 | 0.047779072 |
| TNFRSF1A_AT_19829 | 0.74 | 0.55-1.00 | 0.047779072 |
| SMARCC2_ES_22393 | 0.74 | 0.55-1.00 | 0.047805729 |
| GSTM4_RI_4056 | 0.74 | 0.55-1.00 | 0.047888497 |
| S100A1_AA_7741 | 0.74 | 0.55-1.00 | 0.047920254 |
| ABCD4_AA_28379 | 0.74 | 0.55-1.00 | 0.047925907 |
| ZNF707_ES_85489 | 0.74 | 0.54-1.00 | 0.048005942 |
| CALM2_ES_53491 | 0.74 | 0.55-1.00 | 0.048024473 |
| TRPC4AP_AA_59060 | 1.35 | 1.00-1.82 | 0.04802823 |
| PLA2G16_AP_16517 | 1.35 | 1.00-1.83 | 0.048133328 |
| PDE4D_AP_72135 | 0.74 | 0.55-1.00 | 0.048165174 |
| POLR2H_ES_67947 | 0.74 | 0.55-1.00 | 0.0481864 |
| FHAD1_AT_749 | 0.74 | 0.55-1.00 | 0.048204374 |
| CPSF6_ES_23307 | 0.74 | 0.55-1.00 | 0.048237276 |
| IFT88_ES_25428 | 0.74 | 0.55-1.00 | 0.048260175 |
| TRPM7_AA_30600 | 1.35 | 1.00-1.83 | 0.048267447 |
| IL32_RI_33426 | 0.74 | 0.55-1.00 | 0.048343898 |
| ARMC5_RI_36259 | 0.74 | 0.55-1.00 | 0.048406054 |
| ABCE1_ES_70753 | 0.74 | 0.55-1.00 | 0.048408148 |
| FUBP3_ES_87903 | 0.74 | 0.55-1.00 | 0.048431584 |
| MID1_AP_88464 | 0.74 | 0.55-1.00 | 0.048578164 |
| FASTK_RI_82333 | 0.74 | 0.55-1.00 | 0.048626128 |
| SUPT6H_ES_39943 | 0.74 | 0.55-1.00 | 0.048727323 |
| RPS21_AD_60076 | 0.74 | 0.55-1.00 | 0.048730673 |
| BMP1_AA_82995 | 0.74 | 0.55-1.00 | 0.048736978 |
| MEST_AP_81804 | 1.35 | 1.00-1.82 | 0.048815987 |
| ZMYM6_AT_1710 | 0.74 | 0.55-1.00 | 0.048823173 |
| AMZ2_ES_43134 | 1.35 | 1.00-1.82 | 0.048924886 |
| C19orf82_ES_47378 | 0.74 | 0.55-1.00 | 0.048941354 |
| ZFYVE28_AT_68560 | 1.35 | 1.00-1.82 | 0.048941476 |
| CKMT1A_AA_30308 | 0.74 | 0.55-1.00 | 0.04895882 |
| ARAP1_ES_17641 | 0.74 | 0.55-1.00 | 0.049011782 |
| WBP2_ES_43522 | 0.74 | 0.55-1.00 | 0.049128483 |
| ABR_AP_38285 | 0.74 | 0.55-1.00 | 0.049132193 |
| HAUS8_AP_48223 | 1.35 | 1.00-1.82 | 0.049139181 |
| FAR1_AP_14454 | 0.74 | 0.55-1.00 | 0.049139504 |
| CAPRIN2_ES_20950 | 1.35 | 1.00-1.82 | 0.049164696 |
| FAM13B_ES_73499 | 1.35 | 1.00-1.82 | 0.049195819 |
| METTL22_ES_33897 | 1.35 | 1.00-1.83 | 0.049222871 |
| TEX30_ES_26216 | 1.35 | 1.00-1.82 | 0.049282933 |
| CCDC36_AT_64847 | 0.74 | 0.55-1.00 | 0.049320042 |
| CCDC36_AT_64848 | 1.35 | 1.00-1.83 | 0.049320042 |
| ZNF768_AP_36146 | 0.74 | 0.55-1.00 | 0.049366855 |
| DDX3X_AP_88849 | 1.35 | 1.00-1.82 | 0.049408186 |
| DDX3X_AP_88850 | 0.74 | 0.55-1.00 | 0.049408186 |
| CEP95_AD_43070 | 0.74 | 0.55-1.00 | 0.049409354 |
| PCDH1_AT_73831 | 1.35 | 1.00-1.82 | 0.049437695 |
| PCDH1_AT_73832 | 0.74 | 0.55-1.00 | 0.049437695 |
| IRF3_ES_51012 | 0.74 | 0.55-1.00 | 0.04945384 |
| SNX19_AP_19503 | 1.35 | 1.00-1.82 | 0.049475292 |
| ZNF33A_AA_11310 | 0.74 | 0.54-1.00 | 0.049508584 |
| CDV3_AA_66839 | 1.35 | 1.00-1.82 | 0.049519217 |
| FXR1_ES_67746 | 0.74 | 0.55-1.00 | 0.049534272 |
| DCAF11_ES_26846 | 0.74 | 0.55-1.00 | 0.049534707 |
| RAPH1_AT_57074 | 1.35 | 1.00-1.82 | 0.049566876 |
| MARVELD3_AT_37467 | 1.35 | 1.00-1.82 | 0.049584494 |
| C3orf67_AT_65475 | 0.74 | 0.55-1.00 | 0.049645373 |
| GCAT_ES_62158 | 1.35 | 1.00-1.82 | 0.049778461 |
| RNF213_AT_44047 | 1.35 | 1.00-1.83 | 0.049856632 |
| PTK2B_RI_83157 | 0.74 | 0.55-1.00 | 0.049912845 |
| WRAP53_AP_39042 | 0.74 | 0.55-1.00 | 0.049976811 |

| **Supplementary Table 2: GO and KEGG pathway analysis of prognosis-related AS events by DAVID (https://david.ncifcrf.gov/) website.** This table shows the detailed information of functional enrichment results on prognosis-associated AS genes. In each column, GO/KEGG pathway represents the pathways are from GO or KEGG database; GO/KEGG ID represents the detailed Pathway ID in the GO/KEGG database; Pathway Description represents the detailed Pathway names; Gene Ratio represents the ratio between enriched genes in the pathway versus gene list; Background Ratio represents the ratio between total genes in each pathway versus background; P value represents probability calculated from the modified Fisher Exact test. | | | | | |
| --- | --- | --- | --- | --- | --- |
| GO/KEGG pathway | GO/KEGG ID | Pathway Description | Gene Ratio | Background Ratio | P-value |
| GO BP | GO:0008380 | RNA splicing | 73/1500 | 432/17653 | 9.33E-09 |
| GO BP | GO:0006353 | DNA-templated transcription, termination | 29/1500 | 109/17653 | 1.92E-08 |
| GO BP | GO:0006397 | mRNA processing | 79/1500 | 495/17653 | 3.10E-08 |
| GO BP | GO:0000377 | RNA splicing, via transesterification reactions with bulged adenosine as nucleophile | 55/1500 | 326/17653 | 6.56E-07 |
| GO BP | GO:0000398 | mRNA splicing, via spliceosome | 55/1500 | 326/17653 | 6.56E-07 |
| GO BP | GO:0000375 | RNA splicing, via transesterification reactions | 55/1500 | 329/17653 | 8.83E-07 |
| GO BP | GO:0043484 | regulation of RNA splicing | 27/1500 | 120/17653 | 2.20E-06 |
| GO BP | GO:0006289 | nucleotide-excision repair | 26/1500 | 115/17653 | 3.06E-06 |
| GO BP | GO:0046578 | regulation of Ras protein signal transduction | 43/1500 | 244/17653 | 3.38E-06 |
| GO BP | GO:2001252 | positive regulation of chromosome organization | 32/1500 | 159/17653 | 3.49E-06 |
| GO BP | GO:1903146 | regulation of autophagy of mitochondrion | 15/1500 | 47/17653 | 4.47E-06 |
| GO BP | GO:0006914 | autophagy | 70/1500 | 482/17653 | 6.24E-06 |
| GO BP | GO:0061919 | process utilizing autophagic mechanism | 70/1500 | 482/17653 | 6.24E-06 |
| GO BP | GO:0015931 | nucleobase-containing compound transport | 41/1500 | 240/17653 | 1.24E-05 |
| GO BP | GO:0006369 | termination of RNA polymerase II transcription | 18/1500 | 69/17653 | 1.25E-05 |
| GO BP | GO:0006403 | RNA localization | 40/1500 | 233/17653 | 1.39E-05 |
| GO BP | GO:0006405 | RNA export from nucleus | 29/1500 | 147/17653 | 1.47E-05 |
| GO BP | GO:0051056 | regulation of small GTPase mediated signal transduction | 52/1500 | 339/17653 | 2.16E-05 |
| GO BP | GO:0051169 | nuclear transport | 53/1500 | 351/17653 | 2.81E-05 |
| GO BP | GO:0006283 | transcription-coupled nucleotide-excision repair | 18/1500 | 73/17653 | 2.87E-05 |
| GO BP | GO:0071426 | ribonucleoprotein complex export from nucleus | 26/1500 | 130/17653 | 3.12E-05 |
| GO BP | GO:0000075 | cell cycle checkpoint | 38/1500 | 225/17653 | 3.29E-05 |
| GO BP | GO:0071166 | ribonucleoprotein complex localization | 26/1500 | 131/17653 | 3.59E-05 |
| GO BP | GO:0006913 | nucleocytoplasmic transport | 52/1500 | 347/17653 | 4.09E-05 |
| GO BP | GO:1901990 | regulation of mitotic cell cycle phase transition | 60/1500 | 419/17653 | 4.28E-05 |
| GO BP | GO:0006406 | mRNA export from nucleus | 23/1500 | 111/17653 | 4.92E-05 |
| GO BP | GO:0071427 | mRNA-containing ribonucleoprotein complex export from nucleus | 23/1500 | 111/17653 | 4.92E-05 |
| GO BP | GO:0050657 | nucleic acid transport | 34/1500 | 197/17653 | 5.35E-05 |
| GO BP | GO:0050658 | RNA transport | 34/1500 | 197/17653 | 5.35E-05 |
| GO BP | GO:0000380 | alternative mRNA splicing, via spliceosome | 15/1500 | 57/17653 | 5.83E-05 |
| GO BP | GO:1903008 | organelle disassembly | 22/1500 | 105/17653 | 5.97E-05 |
| GO BP | GO:0006376 | mRNA splice site selection | 10/1500 | 28/17653 | 6.00E-05 |
| GO BP | GO:0000422 | autophagy of mitochondrion | 19/1500 | 84/17653 | 6.29E-05 |
| GO BP | GO:0061726 | mitochondrion disassembly | 19/1500 | 84/17653 | 6.29E-05 |
| GO BP | GO:0000077 | DNA damage checkpoint | 29/1500 | 159/17653 | 6.71E-05 |
| GO BP | GO:1901987 | regulation of cell cycle phase transition | 63/1500 | 454/17653 | 7.18E-05 |
| GO BP | GO:0051236 | establishment of RNA localization | 34/1500 | 200/17653 | 7.31E-05 |
| GO BP | GO:0031570 | DNA integrity checkpoint | 30/1500 | 169/17653 | 8.50E-05 |
| GO BP | GO:0048024 | regulation of mRNA splicing, via spliceosome | 18/1500 | 79/17653 | 8.79E-05 |
| GO BP | GO:0031503 | protein-containing complex localization | 39/1500 | 245/17653 | 9.93E-05 |
| GO BP | GO:0050684 | regulation of mRNA processing | 23/1500 | 117/17653 | 0.000115275 |
| GO BP | GO:0051028 | mRNA transport | 27/1500 | 149/17653 | 0.000131903 |
| GO BP | GO:0010506 | regulation of autophagy | 46/1500 | 310/17653 | 0.000140412 |
| GO BP | GO:0007093 | mitotic cell cycle checkpoint | 29/1500 | 166/17653 | 0.00014788 |
| GO BP | GO:0006363 | termination of RNA polymerase I transcription | 10/1500 | 31/17653 | 0.000160113 |
| GO BP | GO:0031124 | mRNA 3'-end processing | 19/1500 | 90/17653 | 0.00016734 |
| GO BP | GO:0006284 | base-excision repair | 12/1500 | 43/17653 | 0.000172525 |
| GO BP | GO:0006986 | response to unfolded protein | 30/1500 | 179/17653 | 0.00024444 |
| GO BP | GO:0000245 | spliceosomal complex assembly | 14/1500 | 58/17653 | 0.000275709 |
| GO BP | GO:0032204 | regulation of telomere maintenance | 17/1500 | 79/17653 | 0.000285038 |
| GO BP | GO:0016236 | macroautophagy | 43/1500 | 293/17653 | 0.000290939 |
| GO BP | GO:0033044 | regulation of chromosome organization | 45/1500 | 311/17653 | 0.00029354 |
| GO BP | GO:0035966 | response to topologically incorrect protein | 32/1500 | 198/17653 | 0.000304776 |
| GO BP | GO:1902749 | regulation of cell cycle G2/M phase transition | 34/1500 | 216/17653 | 0.000333423 |
| GO BP | GO:0090110 | cargo loading into COPII-coated vesicle | 6/1500 | 13/17653 | 0.000378715 |
| GO BP | GO:1903311 | regulation of mRNA metabolic process | 42/1500 | 289/17653 | 0.000418903 |
| GO BP | GO:0042594 | response to starvation | 29/1500 | 177/17653 | 0.000453424 |
| GO BP | GO:0051168 | nuclear export | 32/1500 | 203/17653 | 0.000478883 |
| GO BP | GO:0044774 | mitotic DNA integrity checkpoint | 21/1500 | 113/17653 | 0.000498236 |
| GO BP | GO:0031058 | positive regulation of histone modification | 17/1500 | 83/17653 | 0.000525434 |
| GO BP | GO:0140056 | organelle localization by membrane tethering | 28/1500 | 171/17653 | 0.000569085 |
| GO BP | GO:0030968 | endoplasmic reticulum unfolded protein response | 23/1500 | 130/17653 | 0.000571587 |
| GO BP | GO:0006360 | transcription by RNA polymerase I | 14/1500 | 62/17653 | 0.000573419 |
| GO BP | GO:0044839 | cell cycle G2/M phase transition | 39/1500 | 267/17653 | 0.000597445 |
| GO BP | GO:0000722 | telomere maintenance via recombination | 6/1500 | 14/17653 | 0.000615214 |
| GO BP | GO:0030207 | chondroitin sulfate catabolic process | 6/1500 | 14/17653 | 0.000615214 |
| GO BP | GO:0006361 | transcription initiation from RNA polymerase I promoter | 10/1500 | 36/17653 | 0.000619832 |
| GO BP | GO:0032205 | negative regulation of telomere maintenance | 10/1500 | 36/17653 | 0.000619832 |
| GO BP | GO:0016573 | histone acetylation | 25/1500 | 147/17653 | 0.000621006 |
| GO BP | GO:0044773 | mitotic DNA damage checkpoint | 20/1500 | 107/17653 | 0.000621394 |
| GO BP | GO:0006362 | transcription elongation from RNA polymerase I promoter | 9/1500 | 30/17653 | 0.000622878 |
| GO BP | GO:0010498 | proteasomal protein catabolic process | 59/1500 | 453/17653 | 0.000640157 |
| GO BP | GO:0010389 | regulation of G2/M transition of mitotic cell cycle | 31/1500 | 198/17653 | 0.000654711 |
| GO BP | GO:0007265 | Ras protein signal transduction | 60/1500 | 463/17653 | 0.000657275 |
| GO BP | GO:0006611 | protein export from nucleus | 29/1500 | 181/17653 | 0.000659014 |
| GO BP | GO:0043967 | histone H4 acetylation | 14/1500 | 63/17653 | 0.000680486 |
| GO BP | GO:0022613 | ribonucleoprotein complex biogenesis | 61/1500 | 475/17653 | 0.000749856 |
| GO BP | GO:0006312 | mitotic recombination | 8/1500 | 25/17653 | 0.000774274 |
| GO BP | GO:0009225 | nucleotide-sugar metabolic process | 10/1500 | 37/17653 | 0.000785467 |
| GO BP | GO:0042149 | cellular response to glucose starvation | 10/1500 | 37/17653 | 0.000785467 |
| GO BP | GO:0016570 | histone modification | 58/1500 | 450/17653 | 0.000914661 |
| GO BP | GO:0043161 | proteasome-mediated ubiquitin-dependent protein catabolic process | 53/1500 | 403/17653 | 0.000942504 |
| GO BP | GO:0048569 | post-embryonic animal organ development | 6/1500 | 15/17653 | 0.000951943 |
| GO BP | GO:0006302 | double-strand break repair | 33/1500 | 220/17653 | 0.000959486 |
| GO BP | GO:0031123 | RNA 3'-end processing | 23/1500 | 135/17653 | 0.000980255 |
| GO BP | GO:0018393 | internal peptidyl-lysine acetylation | 25/1500 | 152/17653 | 0.001024302 |
| GO BP | GO:0070911 | global genome nucleotide-excision repair | 9/1500 | 32/17653 | 0.001045763 |
| GO BP | GO:0000381 | regulation of alternative mRNA splicing, via spliceosome | 11/1500 | 45/17653 | 0.001089805 |
| GO BP | GO:0061640 | cytoskeleton-dependent cytokinesis | 16/1500 | 81/17653 | 0.001142578 |
| GO BP | GO:0006888 | ER to Golgi vesicle-mediated transport | 31/1500 | 205/17653 | 0.001177157 |
| GO BP | GO:0031572 | G2 DNA damage checkpoint | 10/1500 | 39/17653 | 0.001226162 |
| GO BP | GO:0006293 | nucleotide-excision repair, preincision complex stabilization | 7/1500 | 21/17653 | 0.001262594 |
| GO BP | GO:0006295 | nucleotide-excision repair, DNA incision, 3'-to lesion | 7/1500 | 21/17653 | 0.001262594 |
| GO BP | GO:0022406 | membrane docking | 28/1500 | 180/17653 | 0.001288018 |
| GO BP | GO:0000281 | mitotic cytokinesis | 14/1500 | 67/17653 | 0.001293402 |
| GO BP | GO:1904356 | regulation of telomere maintenance via telomere lengthening | 13/1500 | 60/17653 | 0.001341405 |
| GO BP | GO:0000910 | cytokinesis | 27/1500 | 172/17653 | 0.00135737 |
| GO BP | GO:1904357 | negative regulation of telomere maintenance via telomere lengthening | 8/1500 | 27/17653 | 0.001363561 |
| GO BP | GO:1905269 | positive regulation of chromatin organization | 17/1500 | 90/17653 | 0.001367716 |
| GO BP | GO:0010821 | regulation of mitochondrion organization | 33/1500 | 226/17653 | 0.001521756 |
| GO BP | GO:0045930 | negative regulation of mitotic cell cycle | 43/1500 | 319/17653 | 0.001693268 |
| GO BP | GO:0035967 | cellular response to topologically incorrect protein | 26/1500 | 166/17653 | 0.001703265 |
| GO BP | GO:0034620 | cellular response to unfolded protein | 24/1500 | 149/17653 | 0.001712199 |
| GO BP | GO:0006475 | internal protein amino acid acetylation | 25/1500 | 158/17653 | 0.001791458 |
| GO BP | GO:0010212 | response to ionizing radiation | 24/1500 | 150/17653 | 0.001877992 |
| GO BP | GO:0016569 | covalent chromatin modification | 58/1500 | 464/17653 | 0.001885454 |
| GO BP | GO:0000723 | telomere maintenance | 25/1500 | 159/17653 | 0.001958248 |
| GO BP | GO:0018394 | peptidyl-lysine acetylation | 25/1500 | 159/17653 | 0.001958248 |
| GO BP | GO:2000278 | regulation of DNA biosynthetic process | 18/1500 | 101/17653 | 0.001994355 |
| GO BP | GO:0010948 | negative regulation of cell cycle process | 46/1500 | 350/17653 | 0.002000773 |
| GO BP | GO:0031065 | positive regulation of histone deacetylation | 6/1500 | 17/17653 | 0.002029742 |
| GO BP | GO:0006643 | membrane lipid metabolic process | 32/1500 | 221/17653 | 0.002038259 |
| GO BP | GO:0051053 | negative regulation of DNA metabolic process | 22/1500 | 134/17653 | 0.002038879 |
| GO BP | GO:0048193 | Golgi vesicle transport | 47/1500 | 360/17653 | 0.002061451 |
| GO BP | GO:0071168 | protein localization to chromatin | 7/1500 | 23/17653 | 0.002289536 |
| GO BP | GO:0000086 | G2/M transition of mitotic cell cycle | 35/1500 | 250/17653 | 0.00231843 |
| GO BP | GO:2000279 | negative regulation of DNA biosynthetic process | 9/1500 | 36/17653 | 0.002576894 |
| GO BP | GO:0009896 | positive regulation of catabolic process | 50/1500 | 393/17653 | 0.002606429 |
| GO BP | GO:0006310 | DNA recombination | 36/1500 | 261/17653 | 0.002616978 |
| GO BP | GO:1905037 | autophagosome organization | 17/1500 | 96/17653 | 0.0028119 |
| GO BP | GO:0032185 | septin cytoskeleton organization | 6/1500 | 18/17653 | 0.002827819 |
| GO BP | GO:0048384 | retinoic acid receptor signaling pathway | 8/1500 | 30/17653 | 0.00285898 |
| GO BP | GO:0031669 | cellular response to nutrient levels | 30/1500 | 208/17653 | 0.002930077 |
| GO BP | GO:0071897 | DNA biosynthetic process | 30/1500 | 209/17653 | 0.003150439 |
| GO BP | GO:0032754 | positive regulation of interleukin-5 production | 5/1500 | 13/17653 | 0.003169252 |
| GO BP | GO:0034063 | stress granule assembly | 5/1500 | 13/17653 | 0.003169252 |
| GO BP | GO:0050872 | white fat cell differentiation | 5/1500 | 13/17653 | 0.003169252 |
| GO BP | GO:0071391 | cellular response to estrogen stimulus | 5/1500 | 13/17653 | 0.003169252 |
| GO BP | GO:0009267 | cellular response to starvation | 22/1500 | 139/17653 | 0.003254732 |
| GO BP | GO:0034976 | response to endoplasmic reticulum stress | 37/1500 | 274/17653 | 0.003317193 |
| GO BP | GO:0043984 | histone H4-K16 acetylation | 6/1500 | 19/17653 | 0.003839233 |
| GO BP | GO:0048025 | negative regulation of mRNA splicing, via spliceosome | 6/1500 | 19/17653 | 0.003839233 |
| GO BP | GO:0090343 | positive regulation of cell aging | 6/1500 | 19/17653 | 0.003839233 |
| GO BP | GO:1903599 | positive regulation of autophagy of mitochondrion | 6/1500 | 19/17653 | 0.003839233 |
| GO BP | GO:0003148 | outflow tract septum morphogenesis | 7/1500 | 25/17653 | 0.003863056 |
| GO BP | GO:0007033 | vacuole organization | 24/1500 | 159/17653 | 0.004090236 |
| GO BP | GO:0035023 | regulation of Rho protein signal transduction | 21/1500 | 133/17653 | 0.004097615 |
| GO BP | GO:0008637 | apoptotic mitochondrial changes | 20/1500 | 125/17653 | 0.004302302 |
| GO BP | GO:0035065 | regulation of histone acetylation | 11/1500 | 53/17653 | 0.004370609 |
| GO BP | GO:0071826 | ribonucleoprotein complex subunit organization | 31/1500 | 223/17653 | 0.004428886 |
| GO BP | GO:0042770 | signal transduction in response to DNA damage | 21/1500 | 134/17653 | 0.004480212 |
| GO BP | GO:0000209 | protein polyubiquitination | 39/1500 | 298/17653 | 0.004517933 |
| GO BP | GO:0051972 | regulation of telomerase activity | 10/1500 | 46/17653 | 0.004560847 |
| GO BP | GO:0045064 | T-helper 2 cell differentiation | 5/1500 | 14/17653 | 0.004588162 |
| GO BP | GO:0006664 | glycolipid metabolic process | 20/1500 | 126/17653 | 0.004716225 |
| GO BP | GO:1903509 | liposaccharide metabolic process | 20/1500 | 126/17653 | 0.004716225 |
| GO BP | GO:0006839 | mitochondrial transport | 42/1500 | 328/17653 | 0.004881425 |
| GO BP | GO:0007020 | microtubule nucleation | 7/1500 | 26/17653 | 0.004904648 |
| GO BP | GO:0031063 | regulation of histone deacetylation | 7/1500 | 26/17653 | 0.004904648 |
| GO BP | GO:0051984 | positive regulation of chromosome segregation | 7/1500 | 26/17653 | 0.004904648 |
| GO BP | GO:1904353 | regulation of telomere capping | 7/1500 | 26/17653 | 0.004904648 |
| GO BP | GO:2001021 | negative regulation of response to DNA damage stimulus | 13/1500 | 69/17653 | 0.00491666 |
| GO BP | GO:0000045 | autophagosome assembly | 16/1500 | 93/17653 | 0.004942012 |
| GO BP | GO:0016575 | histone deacetylation | 15/1500 | 85/17653 | 0.005007649 |
| GO BP | GO:0032392 | DNA geometric change | 15/1500 | 85/17653 | 0.005007649 |
| GO BP | GO:0032211 | negative regulation of telomere maintenance via telomerase | 6/1500 | 20/17653 | 0.005095573 |
| GO BP | GO:1902115 | regulation of organelle assembly | 24/1500 | 162/17653 | 0.005196611 |
| GO BP | GO:0031056 | regulation of histone modification | 21/1500 | 136/17653 | 0.005334517 |
| GO BP | GO:1902275 | regulation of chromatin organization | 24/1500 | 163/17653 | 0.005616531 |
| GO BP | GO:0032200 | telomere organization | 25/1500 | 172/17653 | 0.005646693 |
| GO BP | GO:0008630 | intrinsic apoptotic signaling pathway in response to DNA damage | 17/1500 | 103/17653 | 0.005907902 |
| GO BP | GO:0016241 | regulation of macroautophagy | 24/1500 | 164/17653 | 0.006064178 |
| GO BP | GO:0035459 | cargo loading into vesicle | 7/1500 | 27/17653 | 0.006144029 |
| GO BP | GO:0010833 | telomere maintenance via telomere lengthening | 14/1500 | 79/17653 | 0.006327644 |
| GO BP | GO:0000921 | septin ring assembly | 5/1500 | 15/17653 | 0.006406272 |
| GO BP | GO:0003417 | growth plate cartilage development | 5/1500 | 15/17653 | 0.006406272 |
| GO BP | GO:0032048 | cardiolipin metabolic process | 5/1500 | 15/17653 | 0.006406272 |
| GO BP | GO:0043153 | entrainment of circadian clock by photoperiod | 6/1500 | 21/17653 | 0.006628765 |
| GO BP | GO:0090312 | positive regulation of protein deacetylation | 6/1500 | 21/17653 | 0.006628765 |
| GO BP | GO:1904380 | endoplasmic reticulum mannose trimming | 6/1500 | 21/17653 | 0.006628765 |
| GO CC | GO:0005938 | cell cortex | 49/1579 | 296/18698 | 3.88E-06 |
| GO CC | GO:0016607 | nuclear speck | 56/1579 | 381/18698 | 3.13E-05 |
| GO CC | GO:0005813 | centrosome | 66/1579 | 490/18698 | 0.000101488 |
| GO CC | GO:0005681 | spliceosomal complex | 32/1579 | 190/18698 | 0.000126166 |
| GO CC | GO:0005819 | spindle | 47/1579 | 324/18698 | 0.000179502 |
| GO CC | GO:0005635 | nuclear envelope | 61/1579 | 455/18698 | 0.000207999 |
| GO CC | GO:0044448 | cell cortex part | 28/1579 | 166/18698 | 0.000315913 |
| GO CC | GO:0005665 | DNA-directed RNA polymerase II, core complex | 7/1579 | 18/18698 | 0.000417637 |
| GO CC | GO:0015629 | actin cytoskeleton | 63/1579 | 487/18698 | 0.000439615 |
| GO CC | GO:0030426 | growth cone | 26/1579 | 154/18698 | 0.000500847 |
| GO CC | GO:0032592 | integral component of mitochondrial membrane | 15/1579 | 70/18698 | 0.000627008 |
| GO CC | GO:0005874 | microtubule | 54/1579 | 408/18698 | 0.000637722 |
| GO CC | GO:0098573 | intrinsic component of mitochondrial membrane | 15/1579 | 71/18698 | 0.00073389 |
| GO CC | GO:0030427 | site of polarized growth | 26/1579 | 159/18698 | 0.000822223 |
| GO CC | GO:0099568 | cytoplasmic region | 59/1579 | 468/18698 | 0.00123633 |
| GO CC | GO:0030117 | membrane coat | 18/1579 | 98/18698 | 0.001309485 |
| GO CC | GO:0048475 | coated membrane | 18/1579 | 98/18698 | 0.001309485 |
| GO CC | GO:0034399 | nuclear periphery | 22/1579 | 131/18698 | 0.001402351 |
| GO CC | GO:0005875 | microtubule associated complex | 24/1579 | 148/18698 | 0.001438161 |
| GO CC | GO:0035770 | ribonucleoprotein granule | 30/1579 | 200/18698 | 0.001453213 |
| GO CC | GO:0030496 | midbody | 26/1579 | 166/18698 | 0.001565649 |
| GO CC | GO:0000776 | kinetochore | 22/1579 | 133/18698 | 0.001714139 |
| GO CC | GO:0071013 | catalytic step 2 spliceosome | 18/1579 | 101/18698 | 0.001865788 |
| GO CC | GO:0031307 | integral component of mitochondrial outer membrane | 7/1579 | 23/18698 | 0.002210387 |
| GO CC | GO:0044452 | nucleolar part | 27/1579 | 179/18698 | 0.002251147 |
| GO CC | GO:0032155 | cell division site part | 13/1579 | 64/18698 | 0.00235318 |
| GO CC | GO:0098687 | chromosomal region | 45/1579 | 347/18698 | 0.002577776 |
| GO CC | GO:0071004 | U2-type prespliceosome | 6/1579 | 18/18698 | 0.002741132 |
| GO CC | GO:0043209 | myelin sheath | 25/1579 | 164/18698 | 0.002776425 |
| GO CC | GO:0036464 | cytoplasmic ribonucleoprotein granule | 28/1579 | 191/18698 | 0.002880808 |
| GO CC | GO:0031306 | intrinsic component of mitochondrial outer membrane | 7/1579 | 24/18698 | 0.002895868 |
| GO CC | GO:0032153 | cell division site | 15/1579 | 81/18698 | 0.00294534 |
| GO CC | GO:0036038 | MKS complex | 5/1579 | 13/18698 | 0.003084764 |
| GO CC | GO:0016591 | DNA-directed RNA polymerase II, holoenzyme | 20/1579 | 123/18698 | 0.003328686 |
| GO CC | GO:0005925 | focal adhesion | 49/1579 | 393/18698 | 0.003807556 |
| GO CC | GO:0005924 | cell-substrate adherens junction | 49/1579 | 396/18698 | 0.004416816 |
| GO CC | GO:0000922 | spindle pole | 23/1579 | 153/18698 | 0.004770143 |
| GO CC | GO:0000145 | exocyst | 6/1579 | 20/18698 | 0.004943794 |
| GO CC | GO:0030119 | AP-type membrane coat adaptor complex | 10/1579 | 47/18698 | 0.005136627 |
| GO CC | GO:0030055 | cell-substrate junction | 49/1579 | 401/18698 | 0.00561777 |
| GO CC | GO:0061695 | transferase complex, transferring phosphorus-containing groups | 36/1579 | 277/18698 | 0.006300397 |
| GO CC | GO:0005685 | U1 snRNP | 6/1579 | 21/18698 | 0.00643418 |
| GO CC | GO:0000428 | DNA-directed RNA polymerase complex | 22/1579 | 148/18698 | 0.006503188 |
| GO CC | GO:0090575 | RNA polymerase II transcription factor complex | 23/1579 | 157/18698 | 0.006547028 |
| GO CC | GO:0016234 | inclusion body | 14/1579 | 80/18698 | 0.006725743 |
| GO CC | GO:0031301 | integral component of organelle membrane | 25/1579 | 177/18698 | 0.007543461 |
| GO CC | GO:0044798 | nuclear transcription factor complex | 26/1579 | 187/18698 | 0.007992824 |
| GO CC | GO:0005741 | mitochondrial outer membrane | 25/1579 | 178/18698 | 0.008092703 |
| GO CC | GO:0001650 | fibrillar center | 20/1579 | 133/18698 | 0.00809404 |
| GO CC | GO:0030880 | RNA polymerase complex | 22/1579 | 151/18698 | 0.008216866 |
| GO CC | GO:0042641 | actomyosin | 12/1579 | 66/18698 | 0.008592914 |
| GO CC | GO:0044322 | endoplasmic reticulum quality control compartment | 7/1579 | 29/18698 | 0.009004386 |
| GO CC | GO:0000793 | condensed chromosome | 29/1579 | 217/18698 | 0.009146794 |
| GO CC | GO:0000123 | histone acetyltransferase complex | 14/1579 | 83/18698 | 0.009315657 |
| GO CC | GO:0044292 | dendrite terminus | 4/1579 | 11/18698 | 0.010309068 |
| GO CC | GO:0071010 | prespliceosome | 6/1579 | 23/18698 | 0.010348566 |
| GO CC | GO:0031968 | organelle outer membrane | 27/1579 | 201/18698 | 0.010871322 |
| GO CC | GO:0030131 | clathrin adaptor complex | 7/1579 | 30/18698 | 0.010907387 |
| GO CC | GO:0032432 | actin filament bundle | 11/1579 | 60/18698 | 0.010914208 |
| GO CC | GO:0031248 | protein acetyltransferase complex | 15/1579 | 93/18698 | 0.010929949 |
| GO CC | GO:1902493 | acetyltransferase complex | 15/1579 | 93/18698 | 0.010929949 |
| GO CC | GO:0010494 | cytoplasmic stress granule | 9/1579 | 45/18698 | 0.011717781 |
| GO CC | GO:0055029 | nuclear DNA-directed RNA polymerase complex | 21/1579 | 147/18698 | 0.011970806 |
| GO CC | GO:0019867 | outer membrane | 27/1579 | 203/18698 | 0.01230541 |
| GO CC | GO:0001725 | stress fiber | 10/1579 | 54/18698 | 0.013880106 |
| GO CC | GO:0032154 | cleavage furrow | 10/1579 | 54/18698 | 0.013880106 |
| GO CC | GO:0097517 | contractile actin filament bundle | 10/1579 | 54/18698 | 0.013880106 |
| GO CC | GO:0000781 | chromosome, telomeric region | 22/1579 | 159/18698 | 0.014632303 |
| GO CC | GO:0099023 | tethering complex | 12/1579 | 71/18698 | 0.015160733 |
| GO CC | GO:0031965 | nuclear membrane | 37/1579 | 304/18698 | 0.015491864 |
| GO CC | GO:0000790 | nuclear chromatin | 42/1579 | 354/18698 | 0.015680141 |
| GO CC | GO:0032806 | carboxy-terminal domain protein kinase complex | 6/1579 | 25/18698 | 0.01570645 |
| GO CC | GO:0016363 | nuclear matrix | 16/1579 | 106/18698 | 0.016119199 |
| GO CC | GO:0005790 | smooth endoplasmic reticulum | 7/1579 | 33/18698 | 0.01834786 |
| GO CC | GO:0016605 | PML body | 15/1579 | 99/18698 | 0.018796466 |
| GO CC | GO:0000803 | sex chromosome | 6/1579 | 26/18698 | 0.018995922 |
| GO CC | GO:0000153 | cytoplasmic ubiquitin ligase complex | 4/1579 | 13/18698 | 0.019492559 |
| GO CC | GO:0000346 | transcription export complex | 4/1579 | 13/18698 | 0.019492559 |
| GO CC | GO:0005675 | holo TFIIH complex | 4/1579 | 13/18698 | 0.019492559 |
| GO CC | GO:0005876 | spindle microtubule | 10/1579 | 57/18698 | 0.01992698 |
| GO CC | GO:0001726 | ruffle | 22/1579 | 164/18698 | 0.020328043 |
| GO CC | GO:0031300 | intrinsic component of organelle membrane | 25/1579 | 193/18698 | 0.021013871 |
| GO CC | GO:0000775 | chromosome, centromeric region | 25/1579 | 194/18698 | 0.02225538 |
| GO CC | GO:0031594 | neuromuscular junction | 10/1579 | 58/18698 | 0.022315481 |
| GO CC | GO:0030864 | cortical actin cytoskeleton | 12/1579 | 75/18698 | 0.022710855 |
| GO CC | GO:0090543 | Flemming body | 6/1579 | 27/18698 | 0.022725503 |
| GO CC | GO:0005905 | clathrin-coated pit | 11/1579 | 67/18698 | 0.023901052 |
| GO CC | GO:0031252 | cell leading edge | 44/1579 | 385/18698 | 0.024488678 |
| GO CC | GO:0005940 | septin ring | 4/1579 | 14/18698 | 0.025502458 |
| GO CC | GO:0030127 | COPII vesicle coat | 4/1579 | 14/18698 | 0.025502458 |
| GO CC | GO:0031105 | septin complex | 4/1579 | 14/18698 | 0.025502458 |
| GO CC | GO:0032160 | septin filament array | 4/1579 | 14/18698 | 0.025502458 |
| GO CC | GO:0030027 | lamellipodium | 24/1579 | 187/18698 | 0.025808911 |
| GO CC | GO:0005777 | peroxisome | 18/1579 | 131/18698 | 0.027088113 |
| GO CC | GO:0042579 | microbody | 18/1579 | 131/18698 | 0.027088113 |
| GO CC | GO:0030863 | cortical cytoskeleton | 15/1579 | 105/18698 | 0.03037498 |
| GO CC | GO:0031235 | intrinsic component of the cytoplasmic side of the plasma membrane | 4/1579 | 15/18698 | 0.032506637 |
| GO CC | GO:0032156 | septin cytoskeleton | 4/1579 | 15/18698 | 0.032506637 |
| GO CC | GO:0012507 | ER to Golgi transport vesicle membrane | 10/1579 | 62/18698 | 0.033956388 |
| GO MF | GO:0045296 | cadherin binding | 52/1513 | 324/17548 | 8.91E-06 |
| GO MF | GO:0140097 | catalytic activity, acting on DNA | 35/1513 | 197/17548 | 3.03E-05 |
| GO MF | GO:0003697 | single-stranded DNA binding | 23/1513 | 107/17548 | 3.36E-05 |
| GO MF | GO:0005089 | Rho guanyl-nucleotide exchange factor activity | 18/1513 | 79/17548 | 0.000105973 |
| GO MF | GO:0050839 | cell adhesion molecule binding | 63/1513 | 489/17548 | 0.000825766 |
| GO MF | GO:0001055 | RNA polymerase II activity | 5/1513 | 11/17548 | 0.001404417 |
| GO MF | GO:0001091 | RNA polymerase II basal transcription factor binding | 6/1513 | 16/17548 | 0.001526405 |
| GO MF | GO:0005096 | GTPase activator activity | 40/1513 | 286/17548 | 0.001581346 |
| GO MF | GO:0003684 | damaged DNA binding | 14/1513 | 68/17548 | 0.001727094 |
| GO MF | GO:0004536 | deoxyribonuclease activity | 14/1513 | 68/17548 | 0.001727094 |
| GO MF | GO:0030695 | GTPase regulator activity | 43/1513 | 316/17548 | 0.001859363 |
| GO MF | GO:0019200 | carbohydrate kinase activity | 7/1513 | 22/17548 | 0.001869568 |
| GO MF | GO:0030676 | Rac guanyl-nucleotide exchange factor activity | 6/1513 | 17/17548 | 0.002188273 |
| GO MF | GO:0000287 | magnesium ion binding | 29/1513 | 196/17548 | 0.002879058 |
| GO MF | GO:0004520 | endodeoxyribonuclease activity | 11/1513 | 50/17548 | 0.003035214 |
| GO MF | GO:0003906 | DNA-(apurinic or apyrimidinic site) endonuclease activity | 5/1513 | 13/17548 | 0.003379496 |
| GO MF | GO:0036002 | pre-mRNA binding | 9/1513 | 37/17548 | 0.003479022 |
| KEGG | hsa04142 | Lysosome | 27/603 | 123/6915 | 5.07E-06 |
| KEGG | hsa03410 | Base excision repair | 10/603 | 33/6915 | 0.000343478 |
| KEGG | hsa00531 | Glycosaminoglycan degradation | 7/603 | 19/6915 | 0.00073444 |
| KEGG | hsa00520 | Amino sugar and nucleotide sugar metabolism | 11/603 | 48/6915 | 0.002299662 |

| **Supplementary Table 3: Prognosis-associated splicing factors.** This table shows the prognosis-associated splicing factors in MIBC with log-rank p value <0.05. In each column, Gene represents detailed prognosis-associated splicing factors; Hazard Ratio(HR) represents the ratio of (risk of outcome in one group)/(risk of outcome in another group), occurring at a given interval of time ; 95%CI represents reliable range of values( 95% confidence interval) in which we expect the true population parameter to be included ; P value measures the statistical significance in log-rank test. | | | |
| --- | --- | --- | --- |
| Gene | Hazard Ratio (HR) | 95%CI | P-value |
| TIA1 | 0.60 | 0.48-0.76 | 1.74E-05 |
| ZRANB2 | 0.63 | 0.44-0.91 | 0.013651659 |
| TRA2A | 0.68 | 0.49-0.93 | 0.015620843 |
| DAZAP1 | 0.63 | 0.43-0.93 | 0.019033936 |
| KHDRBS3 | 1.10 | 1.02-1.19 | 0.020501934 |
| NOVA1 | 1.09 | 1.01-1.17 | 0.02358259 |
| RBM5 | 0.77 | 0.62-0.96 | 0.024219845 |
| HNRNPH3 | 0.66 | 0.45-0.97 | 0.035408936 |
| QKI | 1.25 | 1.01-1.55 | 0.040056552 |
| SFPQ | 0.63 | 0.40-0.98 | 0.041536337 |
